# A Deep‐Red‐Absorbing Osmium(II) Complex as a Photosensitizer for Photodynamic Therapy Inducing Immunogenic Cell Death

**DOI:** 10.1002/anie.8677989

**Published:** 2026-05-11

**Authors:** Yiyi Zhang, Pierre Mesdom, Eduardo Izquierdo‐García, João P. M. António, Ruonan Gao, Bruno Saubaméa, Johanne Seguin, Morgane Moinard, Philippe Arnoux, Céline Frochot, Kevin Cariou, Bich‐Thuy Doan, Gilles Gasser

**Affiliations:** ^1^ Chimie ParisTech, PSL University, CNRS, Institute of Chemistry for Life and Health Sciences Laboratory For Inorganic Chemical Biology Paris France; ^2^ Departament de Química Inorgànica i Orgànica, Secció de Química Orgànica Universitat De Barcelona (UB) Barcelona Spain; ^3^ Research Institute For Medicines (Imed.ULisboa), Faculty of Pharmacy Universidade De Lisboa Lisboa Portugal; ^4^ Cellular and Molecular Imaging Platform (PICMO), US 25 Inserm, UAR 3612 CNRS, Faculté de Pharmacie de Paris Université Paris Cité Paris France; ^5^ Université Paris Cité, CNRS, Inserm Unité de Technologies Chimiques et Biologiques pour la Santé (UTCBS) Paris France; ^6^ Université De Lorraine, CNRS, LRGP Nancy France; ^7^ Chimie ParisTech, PSL University, CNRS, Institute of Chemistry for Life and Health Sciences, Laboratory of Synthesis, Electrochemistry Imaging and Analytical Systems For Diagnosis Paris France

**Keywords:** adaptive immunity, immunogenic cell death, metals in medicine, osmium, photodynamic therapy

## Abstract

Immunogenic cell death (ICD), which converts tumor cells into their own vaccine, plays a pivotal role in the development of novel anticancer therapies. Here, a small series of osmium(II) polypyridyl complexes was synthesized, and their biological activity in the dark and upon light irradiation against various cancer cell lines was studied. The compound **Os2** (bearing two 4,7‐diphenyl‐1,10‐phenanthrolines and one substituted bipyridine ligand) was discovered to be the most effective photosensitizer (PS) for photodynamic therapy (PDT) of this series through the photogeneration of ^1^O_2_ and •OH. In addition, **Os2** was found to exhibit promising toxicity upon deep‐red irradiation under both normoxia and hypoxia. These observations indicate that this PS is working through a mixture of Type‐I and Type‐II mechanisms. More interestingly, upon 740 nm irradiation, **Os2** can stimulate a strong ICD response on CT26 and MCA205 cells both in vitro and in vivo. A comprehensive immune analysis showed that mice vaccinated with **Os2**‐treated CT26‐luc cells boosted the systemic specific adaptive immune responses, including the activation of CD8^+^ T cells and reprogramming of macrophages, leading to effective inhibition of tumor growth. **Os2** is, to the best of our knowledge, the first photoactive osmium‐based complex inducing ICD.

## Graphical abstract

A deep‐red‐excited osmium(II) photosensitizer (**Os2**) induces immunogenic cell death (ICD) under 740 nm irradiation and triggers robust anti‐tumor immune responses both in vitro and in vivo.

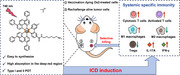

## 1 Introduction

Immunogenic cell death (ICD) is a type of regulated cell death (RCD) that is effective in stimulating adaptive immune responses, inducing systemic antitumor immunity, and maintaining long‐term surveillance [1, 2, 3]. The release of damage‐associated molecular patterns (DAMPs) from dying tumor cells is a crucial event in the ICD process. These signals are either released or exposed to the tumor cell membrane, enhancing the immunogenicity of tumor cells and promoting the maturation and antigen presentation of dendritic cells (DCs), which further stimulates a series of tumor‐specific immune responses [4, 5]. In the future, ICD‐based therapies are expected to use dying tumor cells from cancer patients as a vaccine to stimulate the specific immune response [6].

As research on ICD has progressed, scientists have discovered a variety of methods to induce ICD, including microbial [7, 8], chemical [9, 10], and physical approaches [11, 12]. Photodynamic therapy (PDT) is an approved medical treatment modality with excellent spatiotemporal selectivity and noninvasiveness [13, 14, 15]. In recent years, PDT has also shown great promise in inducing ICD. Some well‐known photosensitizers (PSs) such as hypericin, 5‐aminolevulinic acid (**5‐ALA**)—the precursor of Protoporphyrin IX (**PpIX**)—and chlorin e6 (**Ce6**) have demonstrated excellent ability to induce ICD [16, 17, 18].

Metal‐based PSs are of great interest as PDT‐mediated ICD inducers due to their photostability and ease of structural modification [19, 20, 21]. In 2020, McFarland et al. [22] prepared nine different compounds based on a Ru(II) tris‐heteroleptic scaffold [Ru(NNN)(NN)(L)]Cln and confirmed that one of these compounds could induce ICD both in vitro and in vivo. Two years later, the same group reported two Ru(II)‐based PSs, namely, **ML19B01** and **ML19B02**, that were able to induce ICD in melanoma cells [23]. Sadler, Brabec et al. [24] demonstrated that a photoactivated platinum(IV) prodrug, *trans,trans,trans*‐[Pt(N_3_)_2_(OH)_2_(py)_2_], could induce type II ICD in vitro. The Brabec and Ruiz groups discovered that octahedral Ir(III) complexes could target malignant cancer stem cells (CSCs) and cause ICD in melanoma cells [25]. In 2022, Kodanko et al. [26] showed that a ruthenium complex that can polarize macrophages and promote the cell surface exposure of calreticulin, a known biomarker of ICD. Recently, Chao et al. [27] reported a novel two‐photon absorbing Ir(III)‐based PS, which was very effective in triggering ICD in B16F10‐bearing C57/6J mice. In addition, some examples in the literature demonstrate that zinc complexes can also induce ICD, but all these cases required the use of nanodrug delivery systems [28, 29].

**FIGURE 1:**
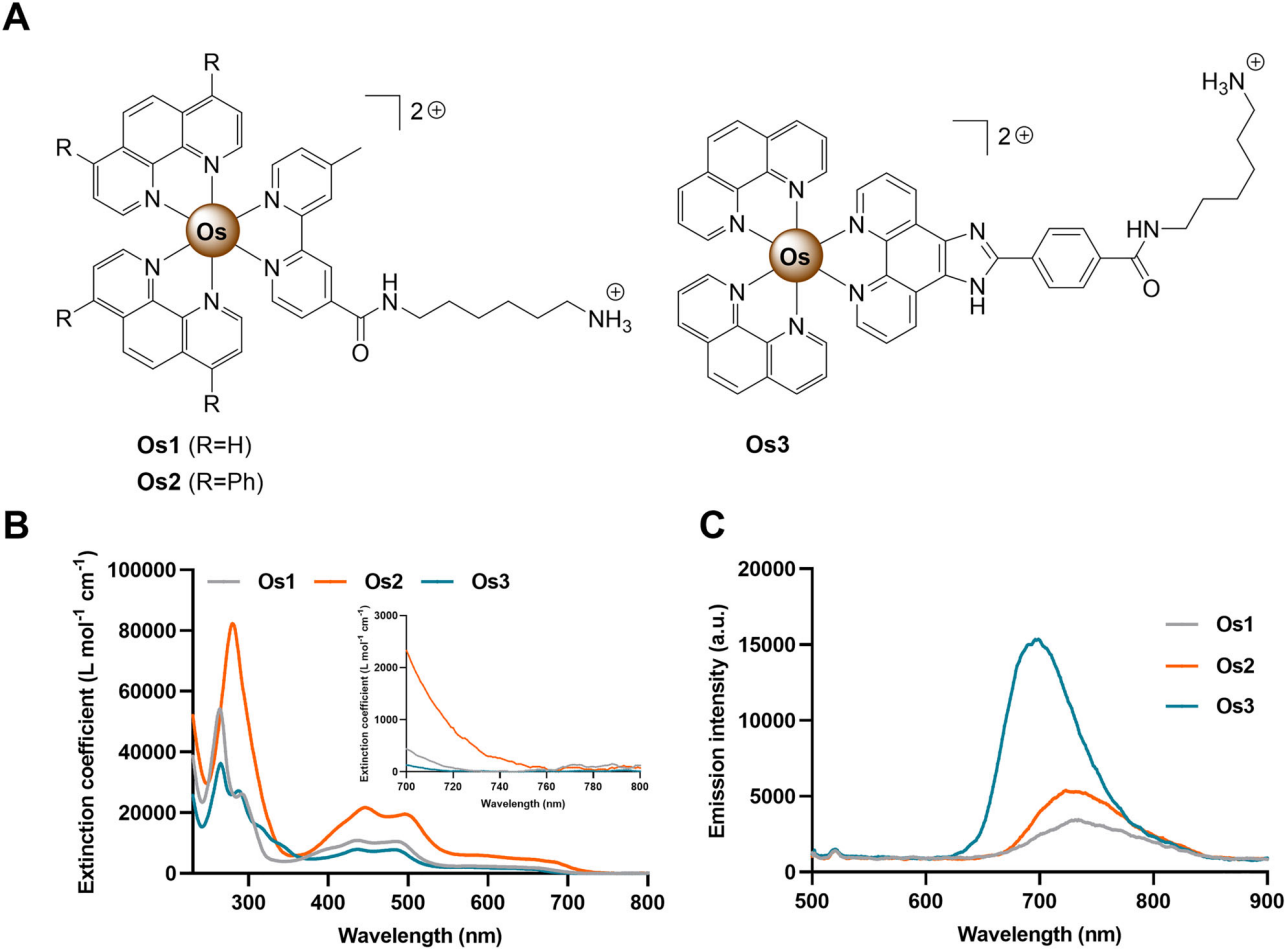
Chemical structures (A), UV/vis absorption spectrum (B), and luminescence emission spectra (λex = 450 nm) (C) in acetonitrile of complexes **Os1‐3**.

**SCHEME 1:**
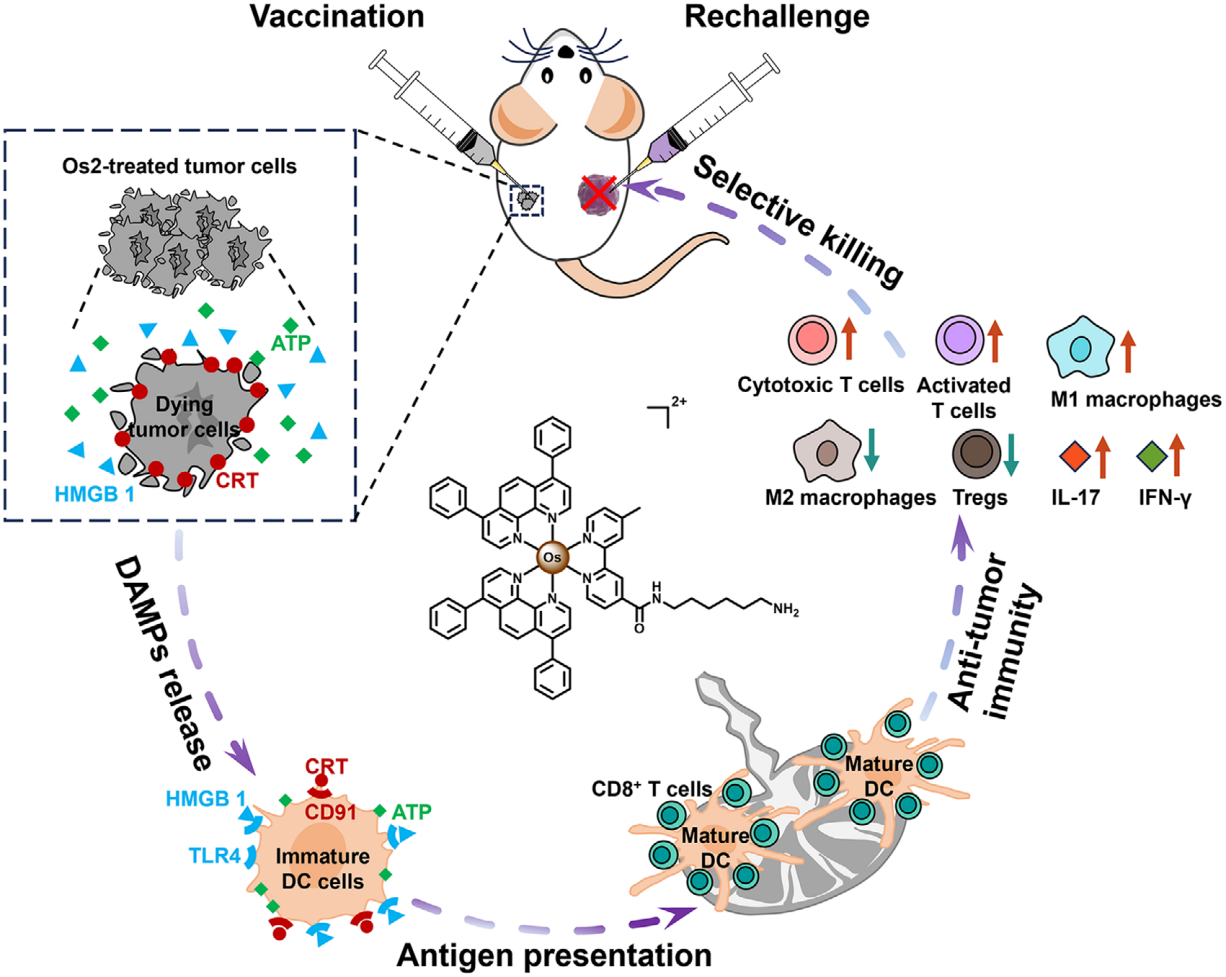
Representation of **Os2**‐induced ICD.

Osmium(II) polypyridyl‐based PSs have distinct advantages over other metal compounds, such as significant absorption at long wavelengths and high stability [30, 31, 32, 33]. The strong spin–orbit coupling (SOC) of the Os(II) center promotes efficient intersystem crossing (ISC), leading to high triplet‐state yields [34, 35]. To the best of our knowledge, no osmium compounds have been reported to induce ICD upon light irradiation to date. To fill this gap, in this work, we synthesized and characterized three osmium‐based PSs **Os1‐3** (Figure 1A). **Os1** and **Os3** are new compounds, while **Os2** has previously been reported as an oxygen sensor [36]. Strikingly, in vitro studies and in vivo vaccination experiments showed that **Os2** induced a strong ICD effect (Scheme 1). It should also be noted that many studies on novel ICD inducers show clear limitations, including incomplete in vitro characterization, lack of in vivo vaccination tests, and the absence of positive controls and non‐ICD chemo‐inducer groups in in vivo studies. Consequently, this work also hopes to provide a reference for standardizing ICD inducer characterization.

## 2 Results and Discussion

### 2.1 Synthesis and Characterization

**SCHEME 2:**
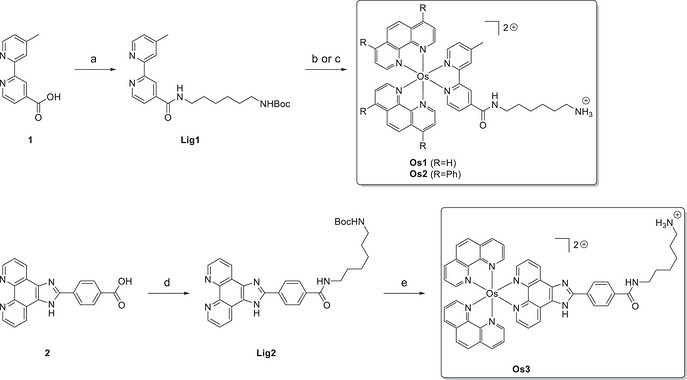
Synthesis of Os(II) polypyridyl complexes **Os1‐3**. Reagents and conditions: (a) *N*‐Boc‐1,6‐hexanediamine, HATU, DMAP, TEA, CH_2_Cl_2_, overnight, rt, 78% yield; (b) 1. Os(phen)_2_Cl_2_, ethylene glycol, 24 h, 90°C, 25%; 2. TFA, CH_2_Cl_2_, 1 h, rt, *quant*.;(c) 1. Os(bphen)_2_Cl_2_, ethylene glycol, 24 h, 110°C; 2. TFA, CH_2_Cl_2_, 2 h, rt, 59% (over two steps) (d) *N*‐Boc‐1,6‐hexanediamine, HATU, DIPEA, DMF, overnight, rt, 51%; (e) 1. Os(phen)_2_Cl_2_, EtOH‐H_2_O (1:1), 24 h, reflux; 2. TFA, CH_2_Cl_2_, 2 h, rt, 23% (over two steps).

The synthetic strategy used for the preparation of the water‐soluble amino‐derivatized Os(II) polypyridyl complexes **Os1‐3** is depicted in Scheme 2. The osmium dichloride precursors **Os(phen)_2_)Cl_2_** and **Os(bphen)_2_)Cl_2_** were obtained by heating ammonium hexachloroosmate with 2 equiv. of either phenantroline (phen) or bathophenanthroline (bphen) in ethylene glycol, according to previously reported protocols [37]. The ligands **Lig1** and **Lig2** could easily be prepared through a HATU‐mediated amide coupling between the corresponding carboxylic acid and *N‐*Boc‐1,6‐hexanediamine. Subsequently, the reaction between the appropriate osmium dichloride synthon OsL_2_Cl_2_ and ligand in either ethylene glycol or a 1:1 mixture of EtOH:H_2_O at high temperature gave the corresponding [OsL'L_2_]^2+^ complex, as a mixture of the *N‐*Boc protected amine and the thermally deprotected free amine. Finally, complete removal of the Boc protecting group was achieved by treatment with trifluoroacetic acid in dichloromethane. The structure of the final amino‐derivatized Os(II) polypyridyl complexes **Os1‐3** (Figure 1A) was determined by ^1^H and ^13^C NMR spectroscopy (Figures S1–S8) and high‐resolution mass spectrometry, and their purity was confirmed by HPLC analysis (Figure S9).

### 2.2 Photophysical Properties

The UV/Vis absorption spectra of the complexes **Os1‐3** were measured in acetonitrile at room temperature. Each showed a profile with three well‐defined absorption bands (Figure 1B). The more intense and sharper peaks between 260–300 nm were assigned to intraligand (IL) ππ* transitions of the phen or bphen ligands, the broad peaks between 350–550 nm (with maxima near 440 and 500 nm) were allocated to the Metal to Ligand Charge Transfer (MLCT) Os(dπ)‐ligand(π*). The weak absorption bands encompassing the region 650–740 nm were attributed to the spin‐forbidden MLCT transitions [38, 39, 40]. The red‐shifted absorption and higher molar extinction coefficient of complex **Os2**, compared to **Os1** and **Os3**, are probably due to the π‐extended structure of the bphen ligands present in **Os2**. Notably, all three compounds are greatly absorbed in the PDT “therapeutic window” (600–800 nm), making them well‐suited for treating large or deep‐seated tumours [35, 41, 42].

Osmium polypyridyl complexes typically present long wavelength emission and a rapid rate of radiationless decay [43]. As shown in Figure 1C, **Os1‐3** are luminescent and show broad emission bands in the red/near‐infrared regions of the spectrum corresponding to [3] MLCT‐based emission [44], with the peaks at 734, 723, and 699 nm, respectively. All compounds show low emission quantum yields around 0.01. The fluorescence lifetimes for **Os1**, **Os2**, and **Os3** are 42.6 ns, 46.1 ns, and 59.0 ns, respectively. Singlet oxygen quantum yields of **Os1**, **Os2**, and **Os3** are 0.11, 0.16, and 0.69, respectively (Table S1).

### 2.3 (Photo)Cytotoxicity In Vitro

To study the phototoxicity of compounds **Os1‐3**, excitation at 620 nm (1.88 mW/cm^2^, 60 min, 6.75 J/cm^2^) was chosen based on their photophysical properties. Given that most ICD studies focus on breast, non‐small cell lung carcinoma, and colon cancer [45, 46], and since ICD was first discovered in mouse colon cancer cells (CT26) [47], we initially tested our compounds on CT26 cells. Cells were incubated with nine different concentrations (mainly from 0.01 to 100 µM) of the compounds for 4 h in the dark. Then, the diluted solutions containing the compounds were replaced by fresh medium, and the cells were either illuminated or kept in the dark for 1 h more. After 2 days, IC_50_ values (concentration of the agent inhibiting cell growth by 50%) were determined with the resazurin assay, using chlorin e6 (**Ce6**) and protoporphyrin IX (**PpIX**) for comparison (Figure S10). The clinically approved chlorin mixture Radachlorin contains ∼80% **Ce6**, while 5‐aminolevulinic acid (Ameluz and Levulan) and methyl 5‐aminolevulinate (Metvix and Metvixia) serve as precursors of **PpIX**. Therefore, **Ce6** and **PpIX** were used for comparison purposes.

The results (Table 1 and Figures S11 and S12) showed that **Os1** exhibited no phototoxicity under illumination, while **Os3** was moderately phototoxic (IC_50_ = 12.11 ± 0.08 µM). Gratifyingly, **Os2** was found to be highly phototoxic (IC_50_ = 0.67 ± 0.04 µM), more than **Ce6** (IC_50_ = 1.98 ± 0.11 µM) and **PpIX** (IC_50_ = 0.91 ± 0.10 µM). Since **Os2** has a very broad absorption spectrum, we next performed a chromatic screening and tested its phototoxicity under eight different wavelengths between 480 and 770 nm (Table 2 and Figures S13 and S14). **Os2** demonstrated superior phototoxicity than **Ce6** across all tested wavelengths. Specifically, **Os2** showed excellent phototoxicity under 510, 540, 595, 645, 670, and 740 nm excitation, with IC_50_ values upon light irradiation consistently ranging from 0.5–1.0 µM, whereas the IC_50_ values of **Ce6** were consistently at least twice higher. It is noteworthy that **Os2** had an IC_50_ value of 0.76 ± 0.05 µM under deep‐red illumination at 740 nm, which is seven times lower than that of **Ce6**. **Os2** retained its phototoxicity even under illumination at 770 nm (IC_50_ = 6.12 ± 0.06 µM), whereas **Ce6** was almost non‐phototoxic under the same conditions.

**TABLE 1:** IC_50_ values in the dark and upon excitation at 620 nm (1.88 mW/cm^2^, 60 min, 6.75 J/cm^2^) for **Os1‐3** compared to **PpIX** and **Ce6** against mouse colon adenocarcinoma (CT26) cells. Data are presented as the mean ± SEM of three independent experiments. PI: Phototoxicity index, IC_50_^dark^/IC_50_^light^. n.d.: Not determinable.

CT26	Dark	620 nm (1 h, 6.75 J/cm^2^)	PI
**Os1**	>100 µM	>100 µM	n.d.
**Os2**	51.60 ± 0.36 µM	0.67 ± 0.04 µM	77
**Os3**	>100 µM	12.11 ± 0.08 µM	>8
**PpIX**	>100 µM	0.91 ± 0.10 µM	>110
**Ce6**	>100 µM	1.98 ± 0.11 µM	>51

**TABLE 2:** IC_50_ values in the dark and upon illumination at 480, 510, 540, 595, 645, 670, 740, and 770 nm for **Os2** and **Ce6** against mouse colon adenocarcinoma (CT26) cells. Data are presented as the mean ± SEM of three independent experiments. PI: Phototoxicity index, IC_50_^dark^/IC_50_^light^. FWHM: Full width at half maximum.

Wavelength (nm)	FWHM (nm)	Dose (J/cm^2^)	Os2		Ce6	
			IC_50_ (µM)	PI	IC_50_ (µM)	PI
480	10	3.38	1.84 ± 0.35	28	2.64 ± 0.42	>38
510	15	2.44	0.64 ± 0.10	81	1.43 ± 0.10	>70
540	15	9.00	0.76 ± 0.02	68	3.33 ± 0.44	>30
595	10	3.38	0.51 ± 0.05	101	5.10 ± 1.44	>20
645	10	9.00	0.67 ± 0.03	77	3.93 ± 0.67	>25
670	10	13.50	0.60 ± 0.06	86	1.97 ± 0.42	>51
740	10	12.60	0.76 ± 0.05	68	5.27 ± 0.46	>19
770	12.5	15.30	6.12 ± 0.06	8	25.37 ± 3.72	>4

Encouraged by the promising results obtained against CT26 cells, we next tested the (photo)toxicity of the compounds on both noncancerous and human cancer cell lines. Similar results to CT26 were obtained with human glioblastoma cells (U‐87 MG). **Os2** exhibited high toxicity under deep‐red illumination at 740 or 770 nm, with significantly lower toxicity in the dark (Table S2 and Figure S15). Notably, the IC_50_ of **Os2** was approximately ten times lower than that of **Ce6** at 740 nm and about five times lower at 770 nm. Selectivity of PSs for cancer cells over noncancerous cells can reduce side effects during treatment. Therefore, we also tested the toxicity of both compounds on mouse fibroblast cells (NIH/3T3) and human fibroblast cells (MRC‐5). The IC_50_ values of **Os2** and **Ce6** on both cell lines were higher than 100 µM, indicating a great selectivity between cancer and noncancerous cells (Table S3 and Figure S16). This selectivity may be attributed to the higher sensitivity of cancer cells to ROS, as cancer cells typically exhibit enhanced metabolic activity and elevated basal ROS levels, rendering them more sensitive to further oxidative stress [48, 49]. Notably, considering the hypoxia microenvironment of solid tumors [50, 51, 52], we further tested the (photo)toxicity of the compounds under hypoxia conditions using a hypoxia cell incubator and a hypoxia chamber (Figure S17). As shown in Table S4 and Figure S18, the IC_50_ results of **Os2** and **Ce6** under hypoxia were similar to those obtained in normoxia. This is extremely important and strengthens our recent results on osmium(II) polypyridyl complexes that showed a similar trend [32]. In summary, **Os2** exhibited outstanding tumor phototoxicity and selectivity, as confirmed by comprehensive cytotoxicity assays.

### 2.4 (Photo)‐Stability Studies

Photostability is a crucial parameter for PSs, as it has a significant impact on their phototoxicity. Based on the results of our in vitro phototoxicity studies, and in view of future in vivo experiments, we decided to investigate the stability of **Os2** and **Ce6** under deep‐red illumination. Therefore, we monitored the UV–vis absorption spectra of **Os2** and **Ce6** in biological media (10% FBS in PBS, phosphate‐buffered saline) using a 740 nm excitation wavelength for one hour and a 770 nm excitation wavelength for 2 h, with the duration of light exposure kept consistent with in vitro phototoxicity tests. As shown in Figure S19, the absorption spectrum of **Os2** remained almost unchanged under excitation at 740 and 770 nm, demonstrating its high photostability. Conversely, when **Ce6** was exposed to light at 740 nm, its absorption spectrum began to weaken noticeably after 10 min and decreased by nearly half after 50 min. After excitation at 770 nm, the UV spectrum of **Ce6** did not change, which could be due to the low power of the 770 nm light or its minimal absorption at this wavelength. Compared to the organic PS **Ce6**, **Os2** exhibits superior photostability, which is one of the general advantages of most metal‐based PSs [53, 54, 55].

### 2.5 Distribution Behavior Studies

The solubility and dispersibility of drug candidates in aqueous solutions or biological media are important properties. We previously observed that Ru(II) and Os(II) polypyridyl complexes could form aggregates in isotonic aqueous or PBS solutions while well‐dispersed in the presence of proteins [32, 56, 57]. Here, dynamic light scattering (DLS) was used to investigate the distribution behavior of **Os2** (Cl^−^ counterion). As shown in Figure S20A, the 10% FBS in PBS solution showed particles with a diameter around 7 nm, which corresponds to the size of bovine serum albumin (BSA), the main component of FBS [58, 59]. The addition of **Os2** hardly changed the size distribution of this solution, indicating its good solubility in this biological medium (Figure S20B). **Os2** was very stable in this biological medium for at least 48 h (Figure S20C–F). In 100% PBS, while no visible **Os2** precipitates were detected, the particle sizes of all tested samples were primarily around 600 nm, with a small peak appearing around 5500 nm for one sample (Figure S20G). This indicates that the solubility of **Os2** in 100% PBS is reduced, resulting in the formation of small or large aggregates. This was also indicated by the polydispersity index (PDI) values of the different conditions. As shown in Figure S20, the PDI values of 10% FBS in PBS solution and **Os2** in this solution both showed < 0.7, indicating the good dispersibility (International Standards Organizations, ISO 22412:2017). However, the PDI value of **Os2** in 100% PBS was higher than 0.7, meaning that **Os2** aggregates. Overall, **Os2** dissolves well in the presence of serum albumin, providing a basis for its favorable biodistribution in vivo.

### 2.6 Subcellular Localization Studies

**FIGURE 2:**
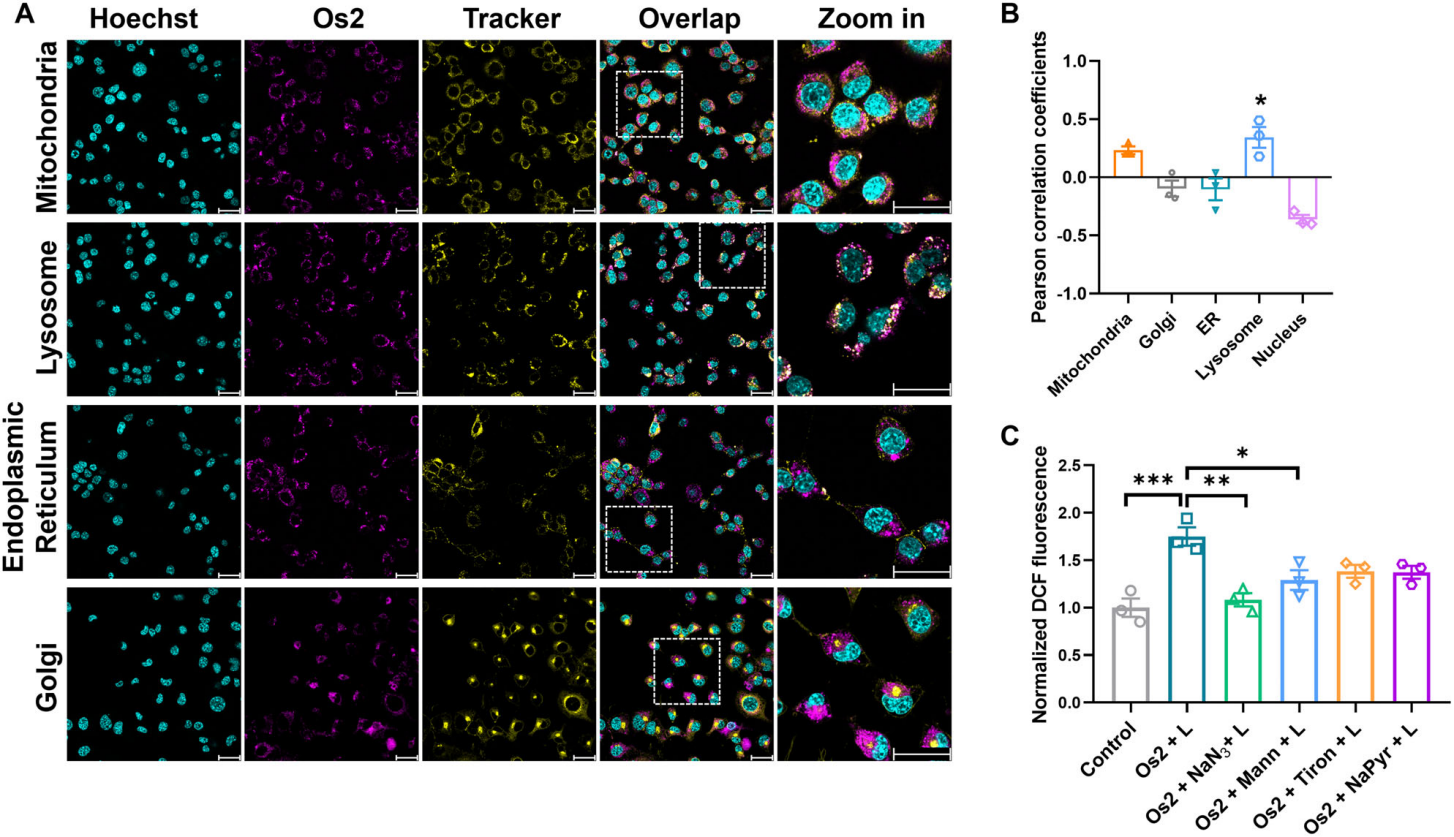
Characterization of **Os2** intracellular localization. (A) Co‐localization of **Os2** (magenta) with organelle‐specific trackers (yellow) in CT26 cells as assessed by confocal microscopy. Cells were incubated for 4 h with 20 µM **Os2**, then with the indicated trackers and finally with Hoechst DNA stain (cyan). Scale bar: 30 µm (B) Pearson correlation coefficients of complex **Os2** and the indicated trackers. Significant difference comparisons are with zero. (C) ROS levels in CT26 cells as assessed by flow cytometry. Cells were incubated with 0.76 µM of compound **Os2** in the presence of the indicated ROS scavengers (sodium azide, D‐mannitol, sodium 4,5‐dihydroxybenzene‐1,3‐disulfonate, sodium pyruvate) for 1 h. After irradiation at 740 nm for 1 h (L), they were incubated with 10 µM DCFH‐DA for 30 min at room temperature in the dark, collected, and analyzed by flow cytometry. All data are shown as mean ± SEM, *n* = 3 independent fields or samples. *p < 0.05, **p < 0.01, and ***p < 0.001 (one‐way ANOVA followed by Tukey's HSD post‐hoc test).

The subcellular localization of **Os2** in CT26 cells was studied using confocal laser scanning microscopy. We first selected the appropriate excitation/emission settings for **Os2** imaging based on its luminescence spectra and ensured that there was no detectable crosstalk between the **Os2** and trackers’ channels (Figure S21). Confocal images suggested that **Os2** was primarily localized in lysosomes and mitochondria (Figure 2A), as confirmed by Pearson's correlation coefficient (PCC) (Figure 2B).

### 2.7 Generation of ROS

We next focused on identifying the specific ROS generated during the PDT treatment with **Os2** by flow cytometry. Based on the in vitro toxicity results, irradiation at 740 nm was selected as the wavelength for these experiments. CT26 cells were previously treated with the measured IC_50_ (0.76 µM) of compound **Os2** in the presence of several selective ROS scavengers, including sodium azide (NaN_3_) for singlet oxygen (^1^O_2_), D‐mannitol (Mann) for hydroxyl radical (•OH), sodium 4,5‐dihydroxybenzene‐1,3‐disulfonate (tiron) for superoxide radical (O_2_^•−^) and sodium pyruvate (NaPyr) for hydrogen peroxide (H_2_O_2_) [60, 61]. As shown in Figures 2C and S22, after irradiation with 740 nm light, a powerful fluorescence signal was detected from the ROS probe DCFH‐DA in **Os2**‐treated cells. NaN_3_ and Mann, but not Tiron or NaPyr, significantly prevented the intracellular production of ROS upon irradiation, suggesting that the main ROS generated were ^1^O_2_ and •OH. While ^1^O_2_ is generated through an energy transfer (type II PDT) process, •OH is produced by electron transfer (type I PDT) photoreactions, suggesting that **Os2** might operate through both type I and type II PDT mechanisms.

To further investigate the generation behavior of ^1^O_2_ and •OH, time‐dependent detection experiments were performed using 9,10‐anthracenediyl‐bis(methylene)dimalonic acid (ABDA) and hydroxyphenyl fluorescein (HPF) as probes for ^1^O_2_ and •OH, respectively [62, 63]. As shown in Figure S23, upon irradiation at 740 nm, a gradual decrease in ABDA absorbance and a corresponding increase in HPF fluorescence were observed over time, indicating the continuous generation of both ^1^O_2_ and •OH. Furthermore, based on the identification of ^1^O_2_ and •OH as the primary ROS generated by **Os2**, we further evaluated their respective contributions to the phototoxicity. As shown in Figure S24A, at lower concentrations of **Os2** (0.5 and 0.75 µM), both scavengers partially reduced the phototoxicity upon illumination, with NaN_3_ showing a more pronounced effect, indicating that ^1^O_2_ was the major contributor to the phototoxicity of **Os2**. In contrast, neither scavenger affected cell viability in the dark, suggesting that ROS were primarily responsible for the light‐induced cytotoxicity (Figure S24B). Collectively, these results confirmed that **Os2** generated both ^1^O_2_ and •OH upon 740 nm irradiation through a combination of type I and type II PDT. Cellular scavenger rescue experiments further demonstrated that ^1^O_2_ played a dominant role in the phototoxicity.

### 2.8 Hallmarks of ICD in Cells

Calreticulin (CALR, also called CRT) translocation, adenosine triphosphate (ATP) release, and high mobility group box 1 (HMGB‐1) protein release are the three main biochemical hallmarks of ICD [64, 65, 66]. During ICD induction, the plasma membrane loses integrity, releasing nucleocytoplasmic HMGB‐1, which further promotes the maturation of DCs [67, 68]. In our study, we used the well‐known ICD‐inducer **Ce6** as a positive control to assess the effects of **Os2**.

**FIGURE 3:**
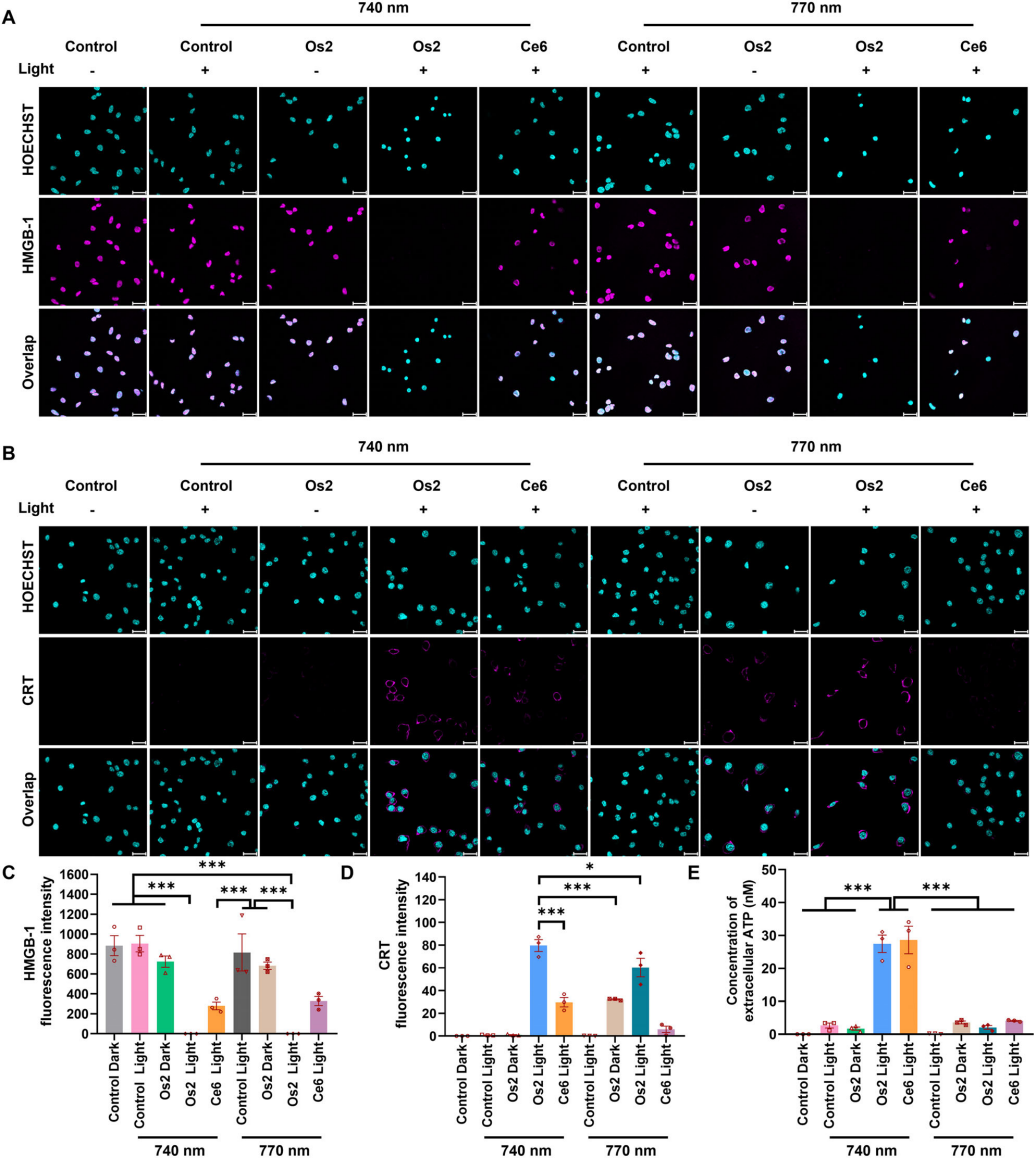
Detection of ICD in vitro. CT26 cells were treated and irradiated as indicated, then immunostained against HMGB‐1 (A) and cell surface CRT (B) (magenta). Nuclear staining with Hoechst 33342 (cyan). (C) Mean fluorescence intensity of corresponding HMGB‐1 fluorescence in A. (D) Mean fluorescence intensity of corresponding CRT fluorescence in B. (E) Release of ATP in cell culture supernatants. All data are shown as mean ± SEM, *n* = 3. *p < 0.05, **p < 0.01, and ***p < 0.001 (one‐way ANOVA followed by Tukey's HSD post‐hoc test). Scale bar: 30 µm.

As shown in Figure 3A,C, HMGB‐1 release was minimal in the absence of light, and remained negligible following illumination at 740 and 770 nm without any compound, indicating that light alone had little impact on the cells. Cells treated with 0.76 and 6.12 µM **Os2** (IC_50_ for 740 and 770 nm light excitation, respectively) showed no significant release of HMGB‐1 in the absence of light. This is consistent with the cytotoxicity results, which show that **Os2** exhibited almost no dark toxicity at these two concentrations. Following illumination at 740 and 770 nm, the release of HMGB‐1 was observed in a minority of **Ce6**‐treated cells. Surprisingly, the HMGB‐1 was released from nearly all **Os2**‐treated cells under both 740 and 770 nm excitation, indicating that **Os2** was an excellent inducer of HMGB‐1 release during PDT.

Next, translocation of ER‐resident calreticulin (endo‐CRT) to the cell surface (ecto‐CRT) was measured by confocal microscopy following treatments with **Ce6** or **Os2** exposed to 740 or 770 nm illumination (Figure 3B,D). In this experiment, cells were fixed but not permeabilized before CRT staining to label only the ecto‐CRT, which is present at the cell surface. A strong signal of ecto‐CRT was detected in **Os2**‐treated cells following 740 or 770 nm illumination, while a weaker, but clearly visible, ecto‐CRT signal was observed in **Ce6‐**treated cells under 740 nm light and in cells treated with 6.12 µM **Os2** in the dark. No surface expression of CRT was observed in the other groups.

We finally measured ATP release, another important event in ICD, using a bioluminescence detection kit (Figure 3E). Cells treated with **Ce6** or **Os2** irradiated at 740 nm showed significantly enhanced ATP release as compared to other conditions, including the **Ce6** or **Os2** irradiated at 770 nm.

In summary, no ICD was observed in the untreated cells, 0.76 µM **Os2**–treated cells without light, and irradiated cells in the absence of compounds. A weak ICD was present in cells treated with 6.12 µM **Os2** in the dark (limited translocation of CRT) and **Ce6**‐treated cells under 770 nm light (partial release of HMGB‐1). **Os2**‐treated cells exposed to 770 nm light showed limited ICD (CRT translocation and HMGB‐1 release, but no significant ATP release). Upon illumination at 740 nm, both **Os2**‐treated and **Ce6**‐treated cells showed strong ICD, with a significant increase observed in all three ICD hallmarks. In comparison, although both displayed similar levels of ATP release, **Os2**‐treated cells exhibited stronger ecto‐CRT expression and greater HMGB‐1 release than **Ce6**‐treated cells. Altogether, our findings indicate that **Os2** can trigger the most potent ICD response following 740 nm light excitation.

### 2.9 Screening of Optimized Doses for In Vivo Vaccination

To comprehensively evaluate the ICD effect of **Os2**, we conducted in vivo experiments with **Ce6** and **Cisplatin** as controls. As previously stated, **Ce6** is a well‐established ICD inducer, making it a suitable positive control group to validate our experimental methods and enable a more accurate assessment of the ICD induced by **Os2**. For negative controls, in addition to the untreated group, we also included a group with an antitumor effect but lacking immunogenic properties to help us more precisely evaluate the impact of ICD. Since there are no well‐known non‐ICD PSs, we used the platinum‐based chemotherapeutic drug **Cisplatin** as a non‐ICD inducer in this study [69, 70].

In typical in vivo ICD experiments, animals are injected at the vaccination site with tumor cells pretreated with a specific drug at an optimal concentration to ensure enough dying cells to trigger an immune response through DAMPs release, while minimizing the number of viable cells to prevent tumor growth at the injection site. Since we had three different drugs, including two PSs and a chemotherapeutic agent, we selected the concentration of each drug corresponding to about 5% of live cells (IC_95_) as the in vivo dose.

**FIGURE 4:**
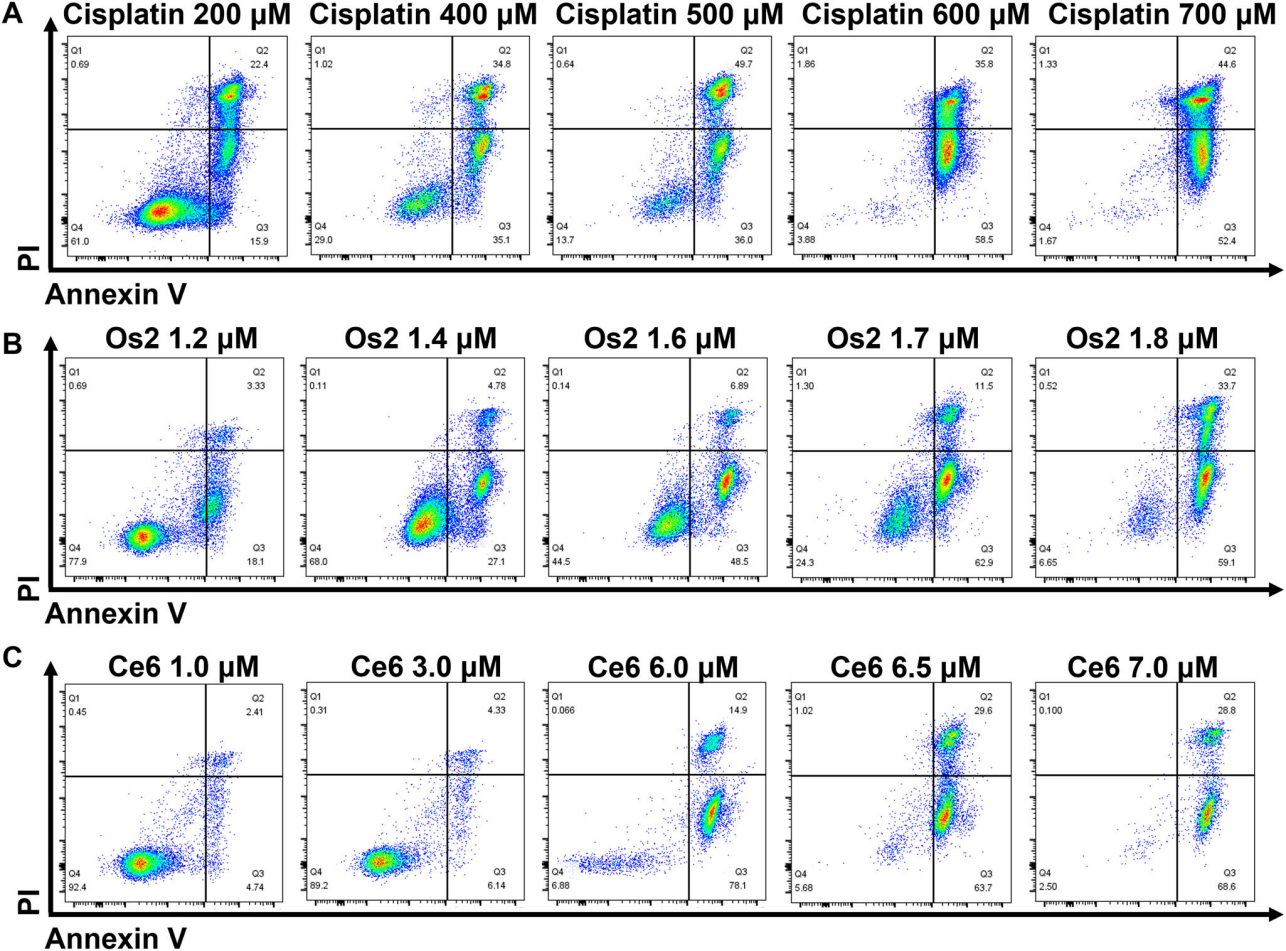
Annexin V‐FITC/propidium iodide (AV/PI) cell viability assay in CT26‐luc cells of **Cisplatin** (A), **Os2** (B), and **Ce6** (C).

Following the gold standard assessment method [70], the Annexin V‐FITC/propidium iodide (AV/PI) dual staining assay was conducted to screen the suitable concentrations of each drug (Figure S25). In addition, to better monitor tumor growth in vivo, luciferase‐labelled tumor cells (CT26‐luc) were used for in vivo experiments by bioluminescence imaging. As shown in Figure 4, treatment with 200 µM **Cisplatin** in the dark, 1.2 µM **Os2** or 1.0 µM **Ce6** upon 740 nm LED light irradiation (60 min, 12.6 J/cm^2^), resulted in most cells remaining alive (Q4, Annexin V^−^/PI^−^). As the concentration of each drug was increased, the proportion of live cells decreased, while the percentage of dying cells (Q3, Annexin V^+^/PI^−^) and dead cells (Q2, Annexin V^+^/PI^+^ and Q1, Annexin V^−^/PI^+^) increased. The concentrations at which approximately 5% of the cells remained alive (IC_95_) were 600 µM for **Cisplatin** in the dark, 1.8 µM for **Os2,** and 6.5 µM for **Ce6** upon 740 nm LED light irradiation (60 min, 12.6 J cm^−2^). These concentrations were selected for the subsequent in vivo vaccination studies.

### 2.10 In Vivo Vaccination and Rechallenge Assays

**FIGURE 5:**
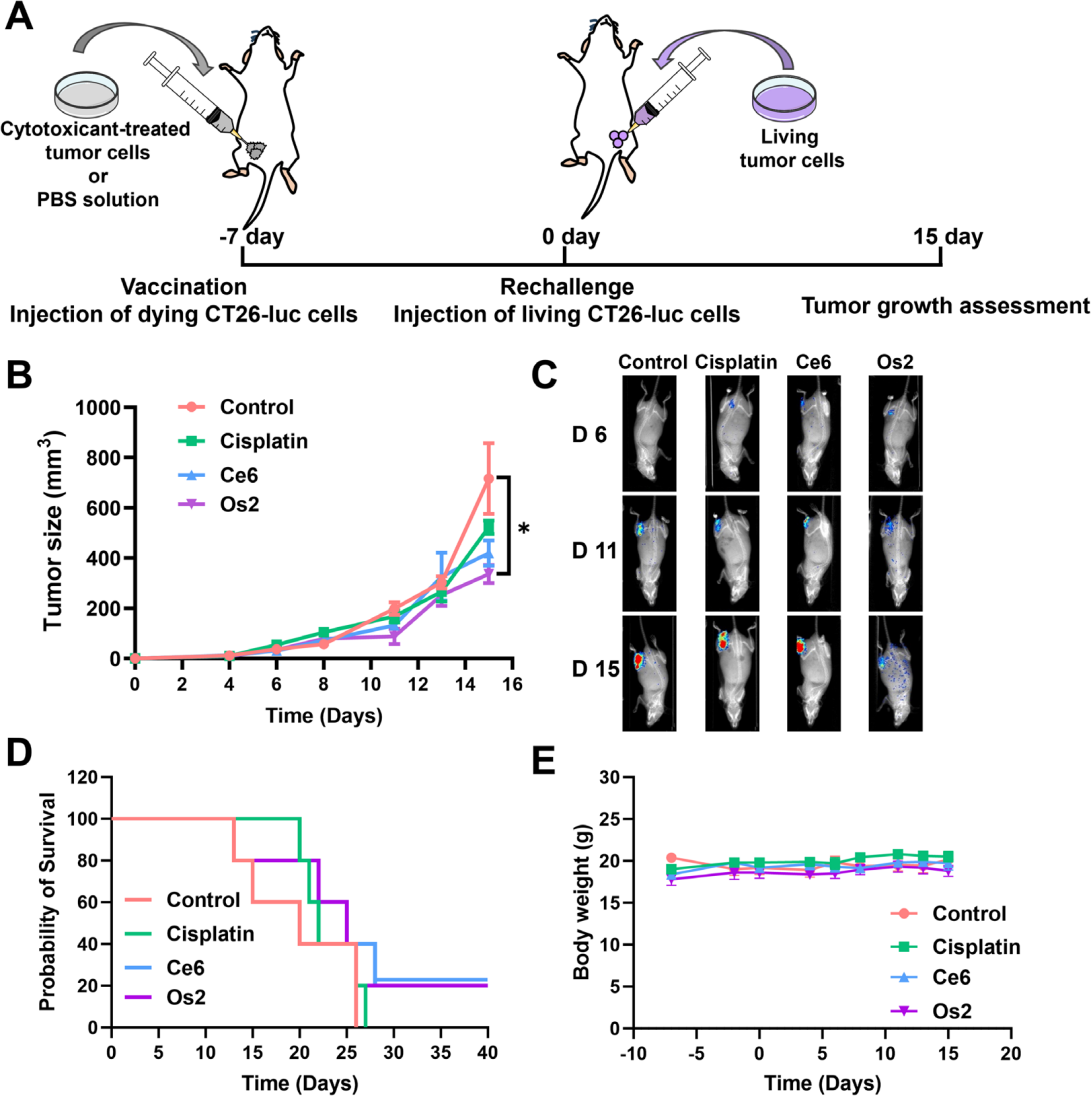
In vivo vaccination and rechallenge studies. (A) Schematic illustration of animal experimental design. (B) Average tumor growth curves of mice in different groups. (C) In vivo bioluminescence imaging of one representative mouse in each treatment group. (D) The survival percentages corresponding to the tumor volumes. (E) The body weight change curve of in different groups. Data are shown as mean ± SEM (*n* = 5 mice per group). *p < 0.05, **p < 0.01 and ***p < 0.001. (one‐way ANOVA followed by Tukey's HSD post‐hoc test).

The ability to stimulate ICD in vivo was evaluated using a CT26‐luc vaccination and rechallenge model, following the gold‐standard method (Figure 5A) [70]. The mice were randomly assigned to groups and injected subcutaneously on the left flank with PBS solution, CT26‐luc cells pre‐treated with either 600 µM **Cisplatin** in the dark, 6.5 µM **Ce6,** or 1.8 µM **Os2** upon 740 nm LED light irradiation (60 min, 12.6 J cm^−2^). After 7 days, all the mice were rechallenged by subcutaneous injection of untreated live CT26‐luc cells on the right flank. Tumor growth was monitored over the following 15 days, and the survival rate was observed for up to 40 days after rechallenge. As shown in Figure 5B, the tumor growth was slow in all mice during the first 13 days. However, by day 15, tumors in the PBS‐treated (control) group grew rapidly, reaching an average tumor volume of 717 ± 190 mm^3^.

The mice in the “**Cisplatin**” group exhibited similar tumor growth to those in the “control” group, showing that **Cisplatin**‐treated dying/dead cells had no ICD effect capable of inhibiting tumor growth, which is consistent with the established knowledge that **Cisplatin** is not an ICD inducer. In comparison, tumor growth in the “**Ce6**” group was partially suppressed and was 1.7 times smaller than in the “control” group on day 15, suggesting that **Ce6** stimulated an effective ICD response. These findings align with the literature, which presents **Ce6** as an ICD inducer [18, 71, 72]. When the **Ce6** group was compared with the control group, the *p‐value* was 0.0657, which is very close to the significance threshold (*p* = 0.05). The high *p‐value* may be explained by the important variation of one of the mouse's growth curves as compared to the other curves in the “**Ce6**” group (Figure S26). Interestingly, tumor growth was significantly inhibited in the “**Os2**” group, with a tumor volume 2.13 times smaller than that of the control group, demonstrating that **Os2** is an excellent ICD inducer and potentially even more effective than **Ce6**.

In parallel, we also recorded bioluminescence signals to monitor the tumor growth, and the mice exhibiting the highest signals in each group are shown as representatives in Figure 5C. On days 6 and 11, there were no significant differences in bioluminescence among the groups. However, on day 15, the “**Os2**” group exhibited considerably weaker bioluminescence signals, indicating reduced functional tumor growth, which is consistent with the tumor measurement results.

In addition, the mice in the “**Os2**” and “**Ce6**” groups exhibited prolonged survival periods compared to those in the other groups (Figure 5D). To our surprise, after day 15, one mouse from the “**Ce6**” group and one from the “**Os2**” group showed a gradual reduction in tumor size, and by day 40, the tumors had been completely ablated in both mice.

Moreover, the body weight of all mice showed negligible differences among all groups throughout the whole period (Figure 5E). Hematoxylin and eosin (H&E) staining of the main organs (heart, liver, lung, spleen, and kidney) revealed no evident histological alterations or signs of inflammation in any of the groups (Figure S27). All these data support the idea that **Os2** possesses a good biosafety profile for in vivo applications.

### 2.11 In Vivo Immune Response Test

**FIGURE 6:**
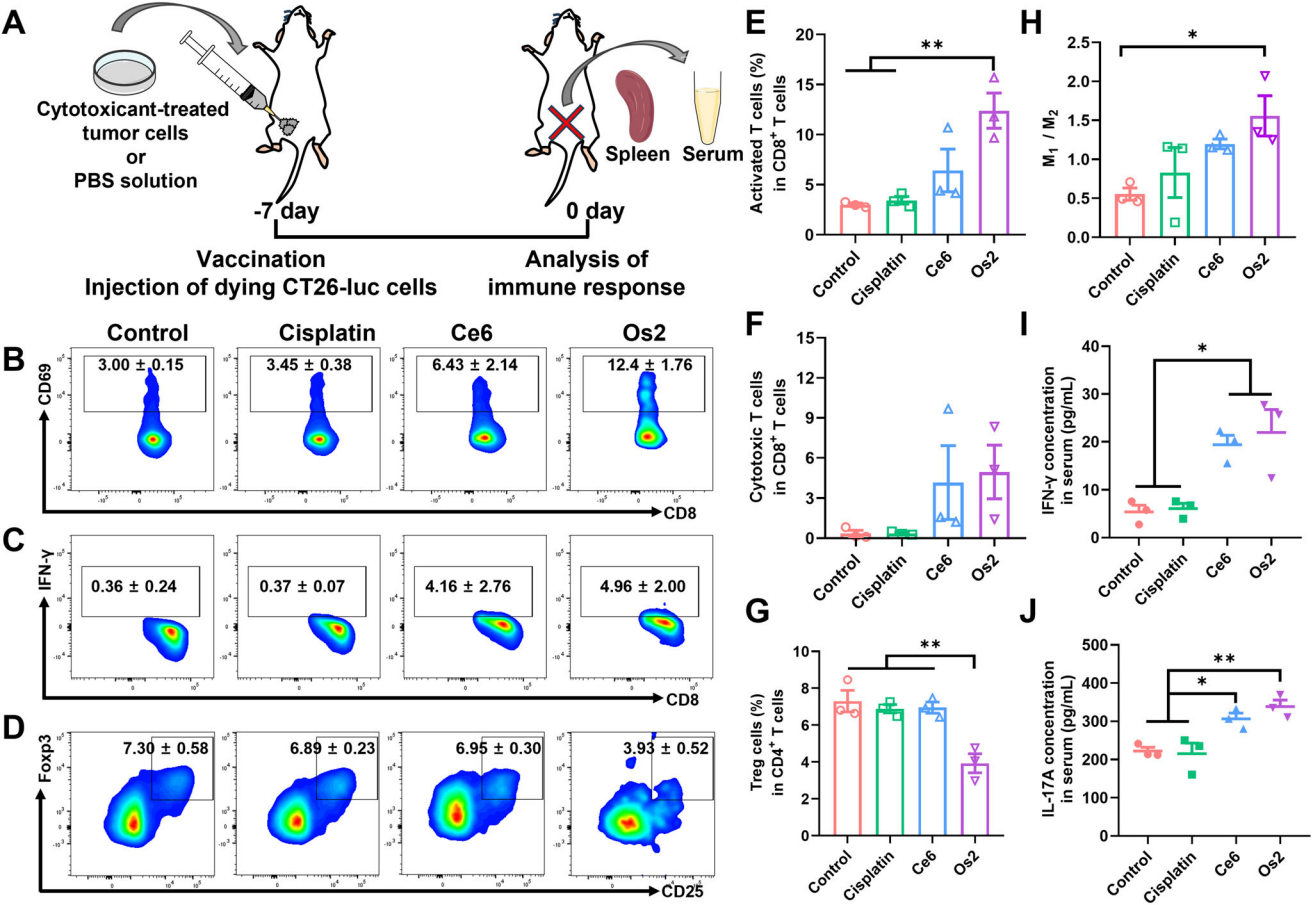
In vivo antitumor immune response studies. (A) Schematic illustration of the animal treatment design. (B–D) Representative flow cytometry analysis of activated CD8^+^ T cells (B), cytotoxic CD8^+^ T cells (C), and Treg cells (D). (E–G) The quantification of activated CD8^+^ T cells (E), cytotoxic CD8^+^ T cells (F), and Treg cells (G). (H) The ratio of M1/M2 in different groups. (I–J) The contents of IFN‐γ (I) and IL‐17A (J) isolated from the serum of the mice in different treatment groups. Data are shown as mean ± SEM (*n* = 3 mice per group). *p < 0.05, **p < 0.01 and ***p < 0.001. (one‐way ANOVA followed by Tukey's HSD post‐hoc test).

Encouraged by the excellent performance of **Os2** in inducing ICD both in vitro and in vivo, we decided to further evaluate the antitumor immune response following vaccination. A key feature of ICD is its ability to initiate antigen‐specific adaptive immune responses [3, 73]. To assess this, we measured the immune cell response in the spleens and the cytokine levels in the serum of mice 7 days after vaccination (Figures 6A and S28).

Activated CD8^+^ T cells (CD45^+^CD3^+^CD8^+^CD69^+^) and cytotoxic CD8^+^ T cells (CD45^+^CD3^+^CD8^+^IFN‐γ^+^) were the most crucial and powerful effectors in the antitumor immune response [74, 75]. Flow cytometry analysis (Figure 6B,E) showed that the percentages of activated CD8^+^ T cells in the “**Ce6**” group were around twice higher than that in the “Control” and “**Cisplatin**” groups. Notably, this value in the “**Os2**” group increased to about 4 times that of the “Control” and “**Cisplatin**” groups, and was twice that of the “**Ce6**” group. For cytotoxic CD8^+^ T cells, the percentages in both “**Ce6**” and “**Os2**” groups were more than 10 times higher than those in the “Control” and “**Cisplatin**” groups (Figure 6C,F). All these results show that **Os2** can effectively enhance the proliferation and activation of CD8^+^ T cells.

We next investigated the levels of tumor regulatory T cells (Tregs, CD45^+^CD3^+^CD25^+^Foxp3^+^), a type of immunosuppressive cell that can suppress effector lymphocytes and promote tumor progression [76, 77]. As shown in Figure 6D,G, **Os2** treatment significantly decreased the number of Tregs, suggesting a reduction in tumor‐associated immunosuppression.

Macrophage polarization and reprogramming have received much attention in recent years in tumor immunotherapy. Tumor‐associated macrophages (TAMs) with an M2‐like phenotype (M2‐like TAMs) are responsible for promoting an immunosuppressive microenvironment, metastasis, and tumor immune escape, while M1‐like TAMs play crucial roles in both innate and adaptive immunity by presenting antigens and inducing antitumor immunity [78, 79]. As shown in Figure 6H and S29, the **Os2**‐treated group exhibited the strongest ability to downregulate M2‐like TAMs (CD11b^+^F4/80^+^CD206^+^) and upregulate M1‐like TAMs (CD11b^+^F4/80^+^CD80^+^). The M1/M2 ratio in this group showed a significant increase compared to the “Control” group.

In addition, the typical inflammation cytokines interferon‐γ (IFN‐γ) and interleukin 17A (IL‐17A) in serum were also detected. IFN‐γ is produced by various immune cells, including effector T cells, natural killer (NK) cells, γδ T cells, and B cells, and is essential for inducing tumor cell apoptosis, enhancing antigen presentation, and promoting T cell priming and activation [80, 81]. IL‐17A is mainly produced by CD4^+^ T helper 17 (Th17) cells and plays a crucial role in connecting the mobilization and activation of neutrophils with T cell activation [82, 83]. Compared to the “Control” and “**Cisplatin**” groups, both the “**Ce6**” and “**Os2**” groups showed a significant increase in the levels of IFN‐γ and IL‐17A, contributing to the activation of the systemic immune response.

Collectively, these results demonstrate that **Ce6‐** and **Os2‐**treated cells can trigger a robust tumor‐specific immune response to prevent or combat tumor relapse. Together with the previous findings, we can conclude that either **Ce6‐** or **Os2**‐treated cells can produce a powerful ICD effect, and when these cells are used for vaccination in mice, they can further induce strong systemic tumor‐specific immunity. As a result, when the mice were rechallenged with live tumor cells, the activated immune system effectively inhibited tumor growth. In addition, the **Os2** group displayed a slower tumor growth and a higher level of immune response compared to the **Ce6** group, suggesting that **Os2** may be a superior ICD inducer than **Ce6**.

### 2.12 In Vivo Validation of Os2‐Induced ICD on MCA205 Cells

To further demonstrate the effectiveness and broad applicability of the **Os2**‐induced ICD, the MCA205 mouse fibrosarcoma cell line on C57BL/6 mice, an excellent model to study the immune response in vivo, was employed [84, 85, 86]. Here, to achieve a more accurate comparison of the **Os2**‐induced ICD effects, we first compared the ICD induction abilities of **Ce6**, **PpIX,** and **Hypercin**, all of which are commonly used PSs as ICD inducers as mentioned above. The one with the strongest ICD effect will be used as the positive control for in vivo vaccination and rechallenge experiments on MCA205 cells.

As shown in Table S5, consistent with the results on CT26 cells, **Os2** demonstrated superior phototoxicity (IC_50_ = 0.47 ± 0.02 µM) than the three other PSs upon 740 nm illumination. **Ce6** (IC_50_ = 1.77 ± 0.38 µM) and **Hypericin** (IC_50_ = 1.66 ± 0.18 µM) also exhibited strong phototoxic effects on MCA205 cells, while **PpIX** (IC_50_ = 4.41 ± 0.09 µM) was weaker in comparison. We then used the corresponding IC_50_ values of each PS to evaluate their in vitro ICD induction on MCA205 cells. Following illumination at 740 nm, HMGB‐1 was released from almost all **Os2**‐treated cells (Figure S30), aligning with the results observed on CT26 cells, reinforcing that **Os2** is a highly effective inducer of HMGB‐1 release during PDT. Among the three positive controls tested, **Ce6** significantly promoted HMGB‐1 release compared to **PpIX** and **Hypericin**, indicating a stronger ICD effect. As a result, we chose **Ce6** as the positive control for our subsequent in vivo tests on MCA205 cells.

Regarding the optimal dose of each drug for in vivo vaccination, we continued to follow the same standards as with CT26‐luc cells, using the concentration of each drug that results in approximately 5% of live cells as the in vivo dose. The testing method remained the same, using the AV/PI dual staining assay. Negative controls still included the untreated group (direct injection of PBS solution) and the non‐ICD inducer **Cisplatin**. Based on the in vitro HMGB‐1 release results, **Ce6** was maintained as the positive control. For a more comprehensive comparison, we also added the platinum‐based chemotherapeutic agent **Oxaliplatin** as a non‐PS ICD inducer [70], serving as an additional positive control.

**FIGURE 7:**
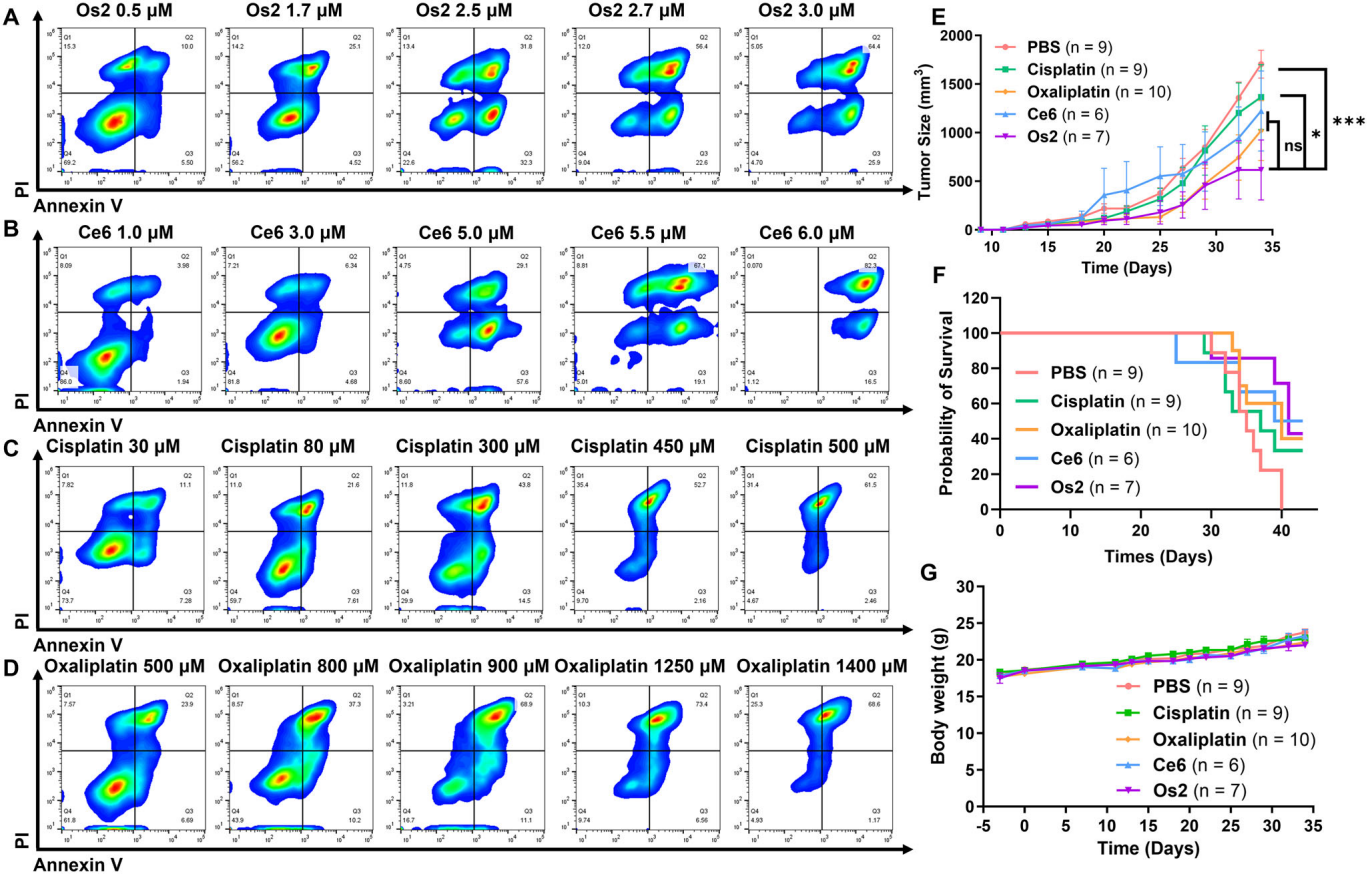
In vivo vaccination and rechallenge studies on MCA205 cells. (A–D) In vitro Annexin V‐FITC/propidium iodide (AV/PI) cell viability assay in MCA205 cells of **Os2** (A), **Ce6** (B), **Cisplatin** (C), and **Oxaliplatin** (D). (E) Average tumor growth curves of mice in different groups. (F) The survival percentages corresponding to the tumor volumes. (G) The body weight change curve in different groups. Data are shown as mean ± SEM (*n* = 6–10 mice per group). *p < 0.05, **p < 0.01 and ***p < 0.001. (two‐way ANOVA followed by Tukey's HSD post‐hoc test).

As shown in Figure 7A–D, exposure to 30 µM **Cisplatin** or 500 µM **Oxaliplatin** in the dark, 0.5 µM **Os2**, or 1.0 µM **Ce6** with 740 nm LED light irradiation (60 min, 12.6 J cm^−2^) resulted in the survival of most MCA205 cells (Q4, Annexin V^−^/PI^−^). As the drug concentration increased, the cells gradually shifted to dying (Q3, Annexin V^+^/PI^−^) or dead (Q2, Annexin V^+^/PI^+^ and Q1, Annexin V^−^/PI^+^) state. For MCA205 cells, the concentrations corresponding to approximately 5% cell survival for each drug were 500 µM **Cisplatin** and 1400 µM **Oxaliplatin** in the dark, 5.5 µM **Ce6** and 3 µM **Os2** under 740 nm LED light illumination.

Next, we used these concentrations in the MCA205 vaccination and rechallenge model on C57BL/6 mice to test the in vivo ICD effect of **Os2**, following the same experimental procedure as that used for CT26‐luc cells in Figure 5A. As shown in Figure 7E, the progression of tumors showed a similar pattern to that observed in the CT26‐luc model. Almost no inhibition was observed in the “**Cisplatin**” group, whereas treatment with “**Ce6**” or “**Oxaliplatin**” resulted in partial suppression. In contrast, tumors in the “**Os2**” group grew markedly slower. By day 34, tumors in the “**Os2**” group were less than half the average volume of tumors in the “PBS” and “**Cisplatin**” groups, and even more than 1.5 times smaller than those in the **Ce6** and **Oxaliplatin** treatment groups, further highlighting the excellent ICD‐inducing capability of **Os2**. In addition, consistent with the in vivo observations in the CT26‐luc model, mice in the “**Os2**” group exhibited significantly prolonged survival compared with the other groups. Body weights remained stable across all groups throughout the study, further supporting the good in vivo biosafety of **Os2**.

In summary, the evaluation of in vivo ICD effects on MCA205 cells demonstrated even stronger efficacy than that observed in the CT26‐luc model. This not only further confirms the excellent in vivo ICD‐inducing capability of **Os2** but also highlights its potential to induce ICD across multiple tumor models.

## 3 Conclusion

In summary, we designed three Os(II) polypyridyl‐based PSs, **Os1**, **Os2,** and **Os3**. Through in vitro toxicity experiments, we showed that **Os2** was the most effective compound in our hands. Taking advantage of the broad absorption spectra characteristic of osmium polypyridyl compounds, we evaluated the phototoxicity of **Os2** under eight different excitation wavelengths, ranging from blue light to deep red. **Os2** was found to be highly toxic upon illumination at 740 nm, even under hypoxic conditions, and retained a certain level of phototoxicity upon 770 nm excitation, which could be particularly beneficial for the treatment of large or deep‐seated tumors. Additionally, **Os2** demonstrated excellent (photo‐)stability, reduced dark toxicity, and high phototoxicity indices. **Os2** is primarily localized in the mitochondria and lysosomes and was shown to operate simultaneously through type I and type II PDT mechanisms. In vitro characterization of the main ICD biomarkers, along with in vivo vaccination and rechallenge assays and in vivo immune response tests, demonstrated that **Os2** was not only able to induce strong ICD effects but also had the potential to surpass the well‐known ICD inducer **Ce6**. Overall, this article demonstrates that **Os2** can effectively induce ICD both in vitro and in vivo, fills the gap in the use of osmium complexes as ICD inducers, and paves the way for the development of metal compounds in immunotherapy and personalized cancer treatment.

## Author Contributions

**Yiyi Zhang**: conceptualization, investigation, writing – original draft, writing – review and editing, data curation, visualization, validation, and methodology. **Pierre Mesdom**: conceptualization, investigation, writing – original draft, data curation, writing – review and editing, visualization, validation, and methodology. **Eduardo Izquierdo‐García**: writing – original draft, investigation, methodology, validation, visualization, writing – review and editing, and data curation. **João P. M. António**: data curation, methodology, validation, visualization, writing – review and editing, writing – original draft, investigation, and conceptualization. **Ruonan Gao**: conceptualization, investigation, writing – original draft, writing – review and editing, visualization, validation, methodology, and data curation. **Bruno Saubaméa**: conceptualization, investigation, writing – original draft, methodology, validation, visualization, writing – review and editing, and data curation. **Johanne Seguin**: investigation, writing – original draft, methodology, validation, visualization, writing – review and editing, and data curation. **Morgane Moinard**: data curation, methodology, validation, visualization, writing – review and editing, writing – original draft, investigation, and conceptualization. **Philippe Arnoux**: conceptualization, investigation, writing – original draft, methodology, validation, visualization, writing – review and editing, and data curation. **Céline Frochot**: conceptualization, funding acquisition, writing – original draft, writing – review and editing, supervision, resources, and project administration. **Kevin Cariou**: funding acquisition, conceptualization, writing – original draft, writing – review and editing, project administration, supervision, and resources. **Bich‐Thuy Doan**: supervision, resources, project administration, writing – review and editing, writing – original draft, funding acquisition, and conceptualization. **Gilles Gasser**: conceptualization, funding acquisition, writing – original draft, writing – review and editing, project administration, supervision, and resources.

## Conflicts of Interest

The authors declare no conflicts of interest.

## Supporting information




**Supporting File 1**: anie72558‐sup‐0001‐SuppMat.pdf.

## Data Availability

The data that support the findings of this study are available from the corresponding author upon reasonable request.

## References

[1] L. Galluzzi , I. Vitale , S. Warren , et al., “Consensus Guidelines for the Definition, Detection and Interpretation of Immunogenic Cell Death,” Journal for ImmunoTherapy of Cancer 8 (2020): e000337, 10.1136/jitc-2019-000337. 32209603 PMC7064135

[2] Z. Deng , H. Li , S. Chen , et al., “Near‐Infrared‐Activated Anticancer Platinum(IV) Complexes Directly Photooxidize Biomolecules in an Oxygen‐Independent Manner,” Nature Chemistry 15 (2023): 930–939, 10.1038/s41557-023-01242-w. 37353602

[3] Y. Zhang , B. T. Doan , and G. Gasser , “Metal‐Based Photosensitizers as Inducers of Regulated Cell Death Mechanisms,” Chemical Reviews 123 (2023): 10135–10155, 10.1021/acs.chemrev.3c00161. 37534710

[4] L. Zhang , N. Montesdeoca , J. Karges , and H. Xiao , “Immunogenic Cell Death Inducing Metal Complexes for Cancer Therapy,” Angewandte Chemie, International Edition in English 62 (2023): e202300662, 10.1002/anie.202300662. 36807420

[5] T. Feng , Z. Tang , J. Karges , et al., “An Iridium(III)‐Based Photosensitizer Disrupting the Mitochondrial Respiratory Chain Induces Ferritinophagy‐Mediated Immunogenic Cell Death,” Chemical Science 15 (2024): 6752–6762, 10.1039/d4sc01214c. 38725496 PMC11077511

[6] S. Sen , M. Won , M. S. Levine , et al., “Metal‐Based Anticancer Agents as Immunogenic Cell Death Inducers: The Past, Present, and Future,” Chemical Society Reviews 51 (2022): 1212–1233, 10.1039/d1cs00417d. 35099487 PMC9398513

[7] O. G. Donnelly , F. Errington‐Mais , L. Steele , et al., “Measles Virus Causes Immunogenic Cell Death in Human Melanoma,” Gene Therapy 20 (2013): 7–15, 10.1038/gt.2011.205. 22170342 PMC3378495

[8] P. K. Bommareddy , M. Shettigar , and H. L. Kaufman , “Integrating Oncolytic Viruses in Combination Cancer Immunotherapy,” Nature Reviews Immunology 18 (2018): 498–513, 10.1038/s41577-018-0014-6. 29743717

[9] J. Pol , E. Vacchelli , F. Aranda , et al., “Trial Watch: Immunogenic Cell Death Inducers for Anticancer Chemotherapy,” Oncoimmunology 4 (2015): e1008866, 10.1080/2162402X.2015.1008866. 26137404 PMC4485780

[10] D. Y. Wong , W. W. Ong , and W. H. Ang , “Induction of Immunogenic Cell Death by Chemotherapeutic Platinum Complexes,” Angewandte Chemie, International Edition in English 54 (2015): 6483–6487, 10.1002/anie.201500934. 25873535

[11] J. Krombach , R. Hennel , N. Brix , et al., “Priming Anti‐Tumor Immunity by Radiotherapy: Dying Tumor Cell‐Derived DAMPs Trigger Endothelial Cell Activation and Recruitment of Myeloid Cells,” Oncoimmunology 8 (2019): e1523097, 10.1080/2162402X.2018.1523097. 30546963 PMC6287777

[12] M. E. Rodriguez‐Ruiz , I. Vitale , K. J. Harrington , I. Melero , and L. Galluzzi , “Immunological Impact of Cell Death Signaling Driven by Radiation on the Tumor Microenvironment,” Nature Immunology 21 (2020): 120–134, 10.1038/s41590-019-0561-4. 31873291

[13] F. Ai , T. Sun , Z. Xu , et al., “An Upconversion Nanoplatform for Simultaneous Photodynamic Therapy and Pt Chemotherapy to Combat Cisplatin Resistance,” Dalton Transactions 45 (2016): 13052–13060, 10.1039/c6dt01404f. 27430044

[14] J. Karges , S. Kuang , F. Maschietto , et al., “Rationally Designed Ruthenium Complexes for 1‐ and 2‐Photon Photodynamic Therapy,” Nature Communications 11 (2020): 3262, 10.1038/s41467-020-16993-0. PMC7320011 32591538

[15] J. Karges , F. Heinemann , M. Jakubaszek , et al., “Rationally Designed Long‐Wavelength Absorbing Ru(II) Polypyridyl Complexes as Photosensitizers for Photodynamic Therapy,” Journal of the American Chemical Society 142 (2020): 6578–6587, 10.1021/jacs.9b13620. 32172564

[16] A. D. Garg , D. V. Krysko , P. Vandenabeele , and P. Agostinis , “Hypericin‐Based Photodynamic Therapy Induces Surface Exposure of Damage‐Associated Molecular Patterns like HSP70 and Calreticulin,” Cancer Immunology, Immunotherapy 61 (2012): 215–221, 10.1007/s00262-011-1184-2. 22193987 PMC11029694

[17] X. Wang , J. Ji , H. Zhang , et al., “Stimulation of Dendritic Cells by DAMPs in ALA‐PDT Treated SCC Tumor Cells,” Oncotarget 6 (2015): 44688–44702, 10.18632/oncotarget.5975. 26625309 PMC4792585

[18] R. Alzeibak , T. A. Mishchenko , N. Y. Shilyagina , I. V. Balalaeva , M. V. Vedunova , and D. V. Krysko , “Targeting Immunogenic Cancer Cell Death by Photodynamic Therapy: Past, Present and Future,” Journal for ImmunoTherapy of Cancer 9 (2021): e001926, 10.1136/jitc-2020-001926. 33431631 PMC7802670

[19] B. S. Howerton , D. K. Heidary , and E. C. Glazer , “Strained Ruthenium Complexes Are Potent Light‐Activated Anticancer Agents,” Journal of the American Chemical Society 134 (2012): 8324–8327, 10.1021/ja3009677. 22553960

[20] J. Karges , “Clinical Development of Metal Complexes as Photosensitizers for Photodynamic Therapy of Cancer,” Angewandte Chemie, International Edition in English 61 (2022): e202112236, 10.1002/anie.202112236. 34748690

[21] S. A. McFarland , A. Mandel , R. Dumoulin‐White , and G. Gasser , “Metal‐Based Photosensitizers for Photodynamic Therapy: the Future of Multimodal Oncology?,” Current Opinion in Chemical Biology 56 (2020): 23–27, 10.1016/j.cbpa.2019.10.004. 31759225 PMC7237330

[22] L. M. Lifshits , J. A. Roque Iii , P. Konda , et al., “Near‐infrared Absorbing Ru(II) Complexes Act as Immunoprotective Photodynamic Therapy (PDT) Agents against Aggressive Melanoma,” Chemical Science 11 (2020): 11740–11762, 10.1039/d0sc03875j. 33976756 PMC8108386

[23] P. Konda , J. A. Roque Iii , L. M. Lifshits , et al., “Photodynamic Therapy of Melanoma With New, Structurally Similar, NIR‐absorbing Ruthenium (II) Complexes Promotes Tumor Growth Control via Distinct Hallmarks of Immunogenic Cell Death,” American Journal of Cancer Research 12 (2022): 210–228. 35141014 PMC8822289

[24] V. Novohradsky , J. Pracharova , J. Kasparkova , et al., “Induction of Immunogenic Cell Death in Cancer Cells by a Photoactivated Platinum(IV) Prodrug,” Inorganic Chemistry Frontiers 7 (2020): 4150–4159, 10.1039/d0qi00991a. 34540235 PMC7611682

[25] G. Vigueras , L. Markova , V. Novohradsky , et al., “A Photoactivated Ir(III) Complex Targets Cancer Stem Cells and Induces Secretion of Damage‐Associated Molecular Patterns in Melanoma Cells Characteristic of Immunogenic Cell Death,” Inorganic Chemistry Frontiers 8 (2021): 4696–4711, 10.1039/d1qi00856k.

[26] N. Toupin , M. K. Herroon , R. P. Thummel , et al., “Metalloimmunotherapy With Rhodium and Ruthenium Complexes: Targeting Tumor‐Associated Macrophages,” Chemistry: A European Journal 28 (2022): e202104430, 10.1002/chem.202104430. 35235227 PMC9541094

[27] L. Wang , J. Karges , F. Wei , et al., “A Mitochondria‐localized Iridium(III) Photosensitizer for Two‐photon Photodynamic Immunotherapy Against Melanoma,” Chemical Science 14 (2023): 1461–1471, 10.1039/d2sc06675k. 36794192 PMC9906708

[28] T. Luo , G. T. Nash , Z. Xu , X. Jiang , J. Liu , and W. Lin , “Nanoscale Metal–Organic Framework Confines Zinc‐Phthalocyanine Photosensitizers for Enhanced Photodynamic Therapy,” Journal of the American Chemical Society 143 (2021): 13519–13524, 10.1021/jacs.1c07379. 34424712 PMC8414475

[29] G. T. Nash , T. Luo , G. Lan , K. Ni , M. Kaufmann , and W. Lin , “Nanoscale Metal–Organic Layer Isolates Phthalocyanines for Efficient Mitochondria‐Targeted Photodynamic Therapy,” Journal of the American Chemical Society 143 (2021): 2194–2199, 10.1021/jacs.0c12330. 33528255 PMC8272603

[30] Y. Sun , L. E. Joyce , N. M. Dickson , and C. Turro , “DNA Photocleavage by an Osmium(ii) Complex in the PDT Window,” Chemical Communications 46 (2010): 6759, 10.1039/c0cc02571b. 20717583

[31] E. C. Glazer , “Panchromatic Osmium Complexes for Photodynamic Therapy: Solutions to Existing Problems and New Questions,” Photochemistry and Photobiology 93 (2017): 1326–1328, 10.1111/php.12796. 28543667 PMC5603404

[32] A. Mani , T. Feng , A. Gandioso , et al., “Structurally Simple Osmium(II) Polypyridyl Complexes as Photosensitizers for Photodynamic Therapy in the Near Infrared,” Angewandte Chemie, International Edition in English 62 (2023): e202218347, 10.1002/anie.202218347. 36917074

[33] L. Gourdon , K. Cariou , and G. Gasser , “Phototherapeutic Anticancer Strategies with First‐row Transition Metal Complexes: A Critical Review,” Chemical Society Reviews 51 (2022): 1167–1195, 10.1039/d1cs00609f. 35048929

[34] M. Hanif , M. V. Babak , and C. G. Hartinger , “Development of Anticancer Agents: Wizardry With Osmium,” Drug Discovery Today 19 (2014): 1640–1648, 10.1016/j.drudis.2014.06.016. 24955838

[35] J. A. Roque 3rd , P. C. Barrett , H. D. Cole , et al., “Breaking the Barrier: An Osmium Photosensitizer With Unprecedented Hypoxic Phototoxicity for Real World Photodynamic Therapy,” Chemical Science 11 (2020): 9784–9806, 10.1039/d0sc03008b. 33738085 PMC7953430

[36] E. J. McLaurin , A. B. Greytak , M. G. Bawendi , and D. G. Nocera , “Two‐Photon Absorbing Nanocrystal Sensors for Ratiometric Detection of Oxygen,” Journal of the American Chemical Society 131 (2009): 12994–13001, 10.1021/ja902712b. 19697933 PMC3340422

[37] W. Xu , K. A. Kneas , J. N. Demas , and B. A. Degraff , “Oxygen Sensors Based on Luminescence Quenching of Metal Complexes: Osmium Complexes Suitable for Laser Diode Excitation,” Analytical Chemistry 68 (1996): 2605–2609, 10.1021/ac960083v. 21619207

[38] S. Lazic , P. Kaspler , G. Shi , et al., “Novel Osmium‐Based Coordination Complexes as Photosensitizers for Panchromatic Photodynamic Therapy,” Photochemistry and Photobiology 93 (2017): 1248–1258, 10.1111/php.12767. 28370264

[39] M. H. Al‐Afyouni , T. N. Rohrabaugh Jr. , K. F. Al‐Afyouni , and C. Turro , “New Ru(II) Photocages Operative with near‐IR Light: New Platform for Drug Delivery in the PDT Window,” Chemical Science 9 (2018): 6711–6720, 10.1039/c8sc02094a. 30310605 PMC6115629

[40] A. Rovira , E. Ortega‐Forte , C. Hally , et al., “Exploring Structure–Activity Relationships in Photodynamic Therapy Anticancer Agents Based on Ir(III)‐COUPY Conjugates,” Journal of Medicinal Chemistry 66 (2023): 7849–7867, 10.1021/acs.jmedchem.3c00189. 37265008 PMC10291553

[41] E. C. Glazer , “Light‐Activated Metal Complexes That Covalently Modify DNA,” Israel Journal of Chemistry 53 (2013): 391–400, 10.1002/ijch.201300019.

[42] Q. Chen , H. Dan , F. Tang , et al., “Photodynamic Therapy Guidelines for the Management of Oral Leucoplakia,” International Journal of Oral Science 11 (2019): 14, 10.1038/s41368-019-0047-0. 30971683 PMC6458125

[43] J. R. Lakowicz , Principles of Fluorescence Spectroscopy (Springer, 2006), 10.1007/978-0-387-46312-4.

[44] K. L. Smitten , P. A. Scattergood , C. Kiker , J. A. Thomas , and P. I. P. Elliott , “Triazole‐based Osmium(II) Complexes Displaying Red/near‐IR Luminescence: Antimicrobial Activity and Super‐Resolution Imaging,” Chemical Science 11 (2020): 8928–8935, 10.1039/d0sc03563g. 34123147 PMC8163367

[45] A. D. Garg , S. More , N. Rufo , et al., “Trial Watch: Immunogenic Cell Death Induction by Anticancer Chemotherapeutics,” Oncoimmunology 6 (2017): e1386829, 10.1080/2162402X.2017.1386829. 29209573 PMC5706600

[46] L. Wang , R. Guan , L. Xie , et al., “An ER‐Targeting Iridium(III) Complex That Induces Immunogenic Cell Death in Non‐Small‐Cell Lung Cancer,” Angewandte Chemie, International Edition in English 60 (2021): 4657–4665, 10.1002/anie.202013987. 33217194

[47] N. Casares , M. O. Pequignot , A. Tesniere , et al., “Caspase‐Dependent Immunogenicity of Doxorubicin‐Induced Tumor Cell Death,” Journal of Experimental Medicine 202 (2005): 1691–1701, 10.1084/jem.20050915. 16365148 PMC2212968

[48] X. An , W. Yu , J. Liu , D. Tang , L. Yang , and X. Chen , “Oxidative Cell Death in Cancer: Mechanisms and Therapeutic Opportunities,” Cell Death & Disease 15 (2024): 556, 10.1038/s41419-024-06939-5. 39090114 PMC11294602

[49] B. Perillo , M. Di Donato , A. Pezone , et al., “ROS in Cancer Therapy: The Bright Side of the Moon,” Experimental & Molecular Medicine 52 (2020): 192–203, 10.1038/s12276-020-0384-2. 32060354 PMC7062874

[50] Q. Cao , D. J. Zhou , Z. Y. Pan , et al., “CAIXplatins: Highly Potent Platinum(IV) Prodrugs Selective Against Carbonic Anhydrase IX for the Treatment of Hypoxic Tumors,” Angewandte Chemie, International Edition in English 59 (2020): 18556–18562, 10.1002/anie.202005362. 32557982

[51] Y. Zheng , Y. Ling , D. Y. Zhang , et al., “Regulating Tumor N^6^‐Methyladenosine Methylation Landscape Using Hypoxia‐Modulating OsS_x_ Nanoparticles,” Small 17 (2021): e2005086, 10.1002/smll.202005086. 33284508

[52] R. Shi , C. Liao , and Q. Zhang , “Hypoxia‐Driven Effects in Cancer: Characterization, Mechanisms, and Therapeutic Implications,” Cells 10 (2021): 678, 10.3390/cells10030678. 33808542 PMC8003323

[53] J. D. Knoll and C. Turro , “Control and Utilization of Ruthenium and Rhodium Metal Complex Excited States for Photoactivated Cancer Therapy,” Coordination Chemistry Reviews 282–283 (2015): 110–126, 10.1016/j.ccr.2014.05.018. PMC4343038 25729089

[54] T. Feng , Z. Tang , J. Shu , et al., “A Cyclometalated Ruthenium(II) Complex Induces Oncosis for Synergistic Activation of Innate and Adaptive Immunity,” Angewandte Chemie, International Edition in English 63 (2024): e202405679, 10.1002/anie.202405679. 38771671

[55] X. Su , W. J. Wang , Q. Cao , et al., “A Carbonic Anhydrase IX (CAIX)‐Anchored Rhenium(I) Photosensitizer Evokes Pyroptosis for Enhanced Anti‐Tumor Immunity,” Angewandte Chemie, International Edition in English 61 (2022): e202115800, 10.1002/anie.202115800. 34842317

[56] A. Notaro , G. Gasser , and A. Castonguay , “Note of Caution for the Aqueous Behaviour of Metal‐Based Drug Candidates,” Chemmedchem 15 (2020): 345–348, 10.1002/cmdc.201900677. 31840945

[57] R. Vinck , A. Gandioso , P. Burckel , B. Saubamea , K. Cariou , and G. Gasser , “Red‐Absorbing Ru(II) Polypyridyl Complexes With Biotin Targeting Spontaneously Assemble into Nanoparticles in Biological Media,” Inorganic Chemistry 61 (2022): 13576–13585, 10.1021/acs.inorgchem.2c02214. 35960605

[58] B. Jachimska and A. Pajor , “Physico‐Chemical Characterization of Bovine Serum Albumin in Solution and as Deposited on Surfaces,” Bioelectrochemistry 87 (2012): 138–146, 10.1016/j.bioelechem.2011.09.004. 22001727

[59] F. I. Abdullah , L. S. Chua , Z. Rahmat , N. Soontorngun , and P. Somboon , “Trypsin Hydrolysed Protein Fractions as Radical Scavengers and Anti‐Bacterial Agents From Ficus Deltoidea,” International Journal of Peptide Research and Therapeutics 24 (2017): 279–290, 10.1007/s10989-017-9613-5.

[60] V. Novohradsky , A. Rovira , C. Hally , et al., “Towards Novel Photodynamic Anticancer Agents Generating Superoxide Anion Radicals: A Cyclometalated Ir III Complex Conjugated to a Far‐Red Emitting Coumarin,” Angewandte Chemie, International Edition in English 131 (2019): 6377–6381, 10.1002/ange.201901268. 30889300

[61] E. Ortega‐Forte , A. Rovira , A. Gandioso , et al., “COUPY Coumarins as Novel Mitochondria‐Targeted Photodynamic Therapy Anticancer Agents,” Journal of Medicinal Chemistry 64 (2021): 17209–17220, 10.1021/acs.jmedchem.1c01254. 34797672 PMC8667040

[62] H. Zhao , J. Joseph , H. Zhang , H. Karoui , and B. Kalyanaraman , “Synthesis and Biochemical Applications of a Solid Cyclic Nitrone Spin Trap: A Relatively Superior Trap for Detecting Superoxide Anions and Glutathiyl Radicals,” Free Radical Biology and Medicine 31 (2001): 599–606, 10.1016/s0891-5849(01)00619-0. 11522444

[63] Y. L. Zeng , L. Y. Liu , T. Z. Ma , et al., “Iridium(III) Photosensitizers Induce Simultaneous Pyroptosis and Ferroptosis for Multi‐Network Synergistic Tumor Immunotherapy,” Angewandte Chemie, International Edition in English 63 (2024): e202410803, 10.1002/anie.202410803. 39180126

[64] D. V. Krysko , A. D. Garg , A. Kaczmarek , O. Krysko , P. Agostinis , and P. Vandenabeele , “Immunogenic Cell Death and DAMPs in Cancer Therapy,” Nature Reviews Cancer 12 (2012): 860–875, 10.1038/nrc3380. 23151605

[65] Z. Deng , N. Wang , Y. Liu , et al., “A Photocaged, Water‐Oxidizing, and Nucleolus‐Targeted Pt(IV) Complex With a Distinct Anticancer Mechanism,” Journal of the American Chemical Society 142 (2020): 7803–7812, 10.1021/jacs.0c00221. 32216337

[66] G. Liu , Y. Zhang , H. Yao , et al., “An Ultrasound‐Activatable Platinum Prodrug for Sono‐Sensitized Chemotherapy,” Science Advances 9 (2023): eadg5964, 10.1126/sciadv.adg5964. 37343091 PMC10284555

[67] L. Galluzzi , A. Buque , O. Kepp , L. Zitvogel , and G. Kroemer , “Immunogenic Cell Death in Cancer and Infectious Disease,” Nature Reviews Immunology 17 (2017): 97–111, 10.1038/nri.2016.107. 27748397

[68] D. Tang , R. Kang , H. J. Zeh , and M. T. Lotze , “The Multifunctional Protein HMGB1: 50 Years of Discovery,” Nature Reviews Immunology 23 (2023): 824–841, 10.1038/s41577-023-00894-6. 37322174

[69] A. Tesniere , F. Schlemmer , V. Boige , et al., “Immunogenic Death of Colon Cancer Cells Treated With Oxaliplatin,” Oncogene 29 (2010): 482–491, 10.1038/onc.2009.356. 19881547

[70] J. Humeau , S. Levesque , G. Kroemer , and J. G. Pol , “Gold Standard Assessment of Immunogenic Cell Death in Oncological Mouse Models,” Methods in Molecular and Cellular Biology 1884 (2019): 297–315, 10.1007/978-1-4939-8885-3_21. 30465212

[71] W. Qiu , M. Liang , Y. Gao , et al., “Polyamino Acid Calcified Nanohybrids Induce Immunogenic Cell Death for Augmented Chemotherapy and Chemo‐Photodynamic Synergistic Therapy,” Theranostics 11 (2021): 9652–9666, 10.7150/thno.64354. 34646391 PMC8490510

[72] T. T. Yu , J. Hu , Q. R. Li , et al., “Chlorin e6‐induced Photodynamic Effect Facilitates Immunogenic Cell Death of Lung Cancer as a Result of Oxidative Endoplasmic Reticulum Stress and DNA Damage,” International Immunopharmacology 115 (2023): 109661, 10.1016/j.intimp.2022.109661. 36608440

[73] G. Kroemer , C. Galassi , L. Zitvogel , and L. Galluzzi , “Immunogenic Cell Stress and Death,” Nature Immunology 23 (2022): 487–500, 10.1038/s41590-022-01132-2. 35145297

[74] H. Raskov , A. Orhan , J. P. Christensen , and I. Gogenur , “Cytotoxic CD8+ T Cells in Cancer and Cancer Immunotherapy,” British Journal of Cancer 124 (2021): 359–367, 10.1038/s41416-020-01048-4. 32929195 PMC7853123

[75] M. Reina‐Campos , N. E. Scharping , and A. W. Goldrath , “CD8+ T Cell Metabolism in Infection and Cancer,” Nature Reviews Immunology 21 (2021): 718–738, 10.1038/s41577-021-00537-8. PMC8806153 33981085

[76] Y. Tie , F. Tang , Y. Q. Wei , and X. W. Wei , “Immunosuppressive Cells in Cancer: Mechanisms and Potential Therapeutic Targets,” Journal of Hematology & Oncology 15 (2022): 61, 10.1186/s13045-022-01282-8. 35585567 PMC9118588

[77] Y. Togashi , K. Shitara , and H. Nishikawa , “Regulatory T Cells in Cancer Immunosuppression — Implications for Anticancer Therapy,” Nature Reviews Clinical Oncology 16 (2019): 356–371, 10.1038/s41571-019-0175-7. 30705439

[78] S. K. Biswas and A. Mantovani , “Macrophage Plasticity and Interaction With Lymphocyte Subsets: Cancer as a Paradigm,” Nature Immunology 11 (2010): 889–896, 10.1038/ni.1937. 20856220

[79] D. G. DeNardo and B. Ruffell , “Macrophages as Regulators of Tumour Immunity and Immunotherapy,” Nature Reviews Immunology 19 (2019): 369–382, 10.1038/s41577-019-0127-6. PMC7339861 30718830

[80] W. Du , T. L. Frankel , M. Green , and W. Zou , “IFNγ Signaling Integrity in Colorectal Cancer Immunity and Immunotherapy,” Cellular & Molecular Immunology 19 (2022): 23–32, 10.1038/s41423-021-00735-3. 34385592 PMC8752802

[81] A. M. Gocher , C. J. Workman , and D. A. A. Vignali , “Interferon‐γ: Teammate or Opponent in the Tumour Microenvironment?,” Nature Reviews Immunology 22 (2022): 158–172, 10.1038/s41577-021-00566-3. PMC8688586 34155388

[82] C. Rodriguez , C. L. Araujo Furlan , J. Tosello Boari , et al., “Interleukin‐17 Signaling Influences CD8^+^T Cell Immunity and Tumor Progression According to the IL‐17 Receptor Subunit Expression Pattern in Cancer Cells,”Oncoimmunology 12 (2023): 2261326, 10.1080/2162402X.2023.2261326. 37808403 PMC10557545

[83] C. Zenobia and G. Hajishengallis , “Basic Biology and Role of Interleukin‐17 in Immunity and Inflammation,” Periodontology 2000 69 (2015): 142–159, 10.1111/prd.12083. 26252407 PMC4530463

[84] M. Michaud , I. Martins , A. Q. Sukkurwala , et al., “Autophagy‐Dependent Anticancer Immune Responses Induced by Chemotherapeutic Agents in Mice,” Science 334 (2011): 1573–1577, 10.1126/science.1208347. 22174255

[85] P. Liu , L. Zhao , J. Pol , et al., “Crizotinib‐Induced Immunogenic Cell Death in Non‐Small Cell Lung Cancer,” Nature Communications 10 (2019): 1486, 10.1038/s41467-019-09415-3. PMC6445096 30940805

[86] L. Galluzzi , J. M. Bravo‐San Pedro , S. Demaria , S. C. Formenti , and G. Kroemer , “Activating Autophagy to Potentiate Immunogenic Chemotherapy and Radiation Therapy,” Nature Reviews Clinical Oncology 14 (2017): 247–258, 10.1038/nrclinonc.2016.183. 27845767

