# Supplementary material for: A Deep‐Red‐Absorbing Osmium(II) Complex as a Photosensitizer for Photodynamic Therapy Inducing Immunogenic Cell Death
Source: Angew Chem Int Ed Engl. 2026 May 11;65(27):e8677989. doi: 10.1002/anie.8677989 (PMC13327620; doi:10.1002/anie.8677989)
Supplement: Supplementary file 1 — Supporting File 1: anie72558‐sup‐0001‐SuppMat.pdf. [file ANIE-65-e8677989-s001.pdf]

A Deep-Red-Absorbing Osmium(II) Complex as a Photosensitizer for Photodynamic Therapy inducing Immunogenic Cell Death

Yiyi Zhang,[a] Pierre Mesdom,[a] Eduardo Izquierdo-García,[a,b] João P. M. António,[a,c] Ruonan Gao,[a] Bruno Saubaméa,[d] Johanne Seguin,[e] Morgane Moinard,[f] Philippe Arnoux,[f] Céline Frochot,[f] Kevin Cariou,[a] Bich-Thuy Doan[g] and Gilles Gasser[a,]*

a. Chimie ParisTech, PSL University, CNRS, Institute of Chemistry for Life and Health Sciences, Laboratory for Inorganic Chemical Biology, 75005 Paris, France.

b. Departament de Química Inorgànica i Orgànica, Secció de Química Orgànica, Universitat de Barcelona (UB), and Institut de Biomedicina de la Universitat de Barcelona (IBUB), Martí i Franquès 1-11, E-08028 Barcelona, Spain.

c. Research Institute for Medicines (iMed.ULisboa), Faculty of Pharmacy, Universidade de Lisboa, Av. Prof. Gama Pinto, 1649-003, Lisboa, Portugal

d. Cellular and Molecular Imaging Platform (PICMO), US 25 Inserm, UAR 3612 CNRS, Faculté de Pharmacie de Paris, Université Paris Cité, 75006 Paris, France.

e. Université Paris Cité, CNRS, Inserm, Unité de Technologies Chimiques et Biologiques pour la Santé (UTCBS), 75006 Paris, France.

f. Université de Lorraine, CNRS, LRGP, F-54 000 Nancy, France.

g. Chimie ParisTech, PSL University, CNRS, Institute of Chemistry for Life and Health Sciences, Laboratory of Synthesis, Electrochemistry, Imaging and Analytical Systems for Diagnosis, 75005 Paris, France.

Contents

Figure S1. ^{1}H NMR spectrum of precursor **Os(phen)$_2$Cl$_2$** in *d$_6$*-DMSO. 26
Figure S2. ^{1}H NMR spectrum of precursor **Os(bphen)$_2$Cl$_2$** in CD_2Cl_2. 27
Figure S3. ^{1}H NMR spectrum of ligand **Lig1** in $CDCl_3$..28
Figure S4. ^{1}H NMR spectrum of complex **Os1(Boc)** in CD_3CN. 29
Figure S5. ^{1}H NMR (top) and ^{13}C NMR (bottom) spectrum of complex **Os1** in CD_3CN.............30
Figure S6. ^{1}H NMR spectrum of complex **Os2** in CD_3OD. 31
Figure S7. ^{1}H NMR (top) and ^{13}C NMR (bottom) spectra of ligand **Lig2** in CD_3OD...................32
Figure S8. ^{1}H NMR (top) and ^{13}C NMR (bottom) spectra of complex **Os3** in CD_3CN. 33
Figure S9. HPLC chromatograms of ligands **Lig1–2** (top) and Os(II) complexes **Os1–3** (bottom), recorded at 260 nm...34
Figure S10. Chemical structures (A), UV/Vis absorption spectrum (B) and luminescence emission spectra (λex = 420 nm) (C) in ethanol of Protoporphyrin IX (**PpIX**) and Chlorin e6 (**Ce6**). 35
Table S1. Photophysical properties of **Os1-3** excited at 450 nm in acetonitrile, **Ce6** and **PpIX** in ethanol...36
Figure S11. Fluorometric cell viability assay of complex **Os1** (A), **Os2** (B), **Os3** (C), **PpIX** (D) and **Ce6** (E) in normoxia conditions in CT26 cells upon illumination at 620 nm. 37
Figure S12. Fluorometric cell viability assay of complex **Os1** (A), **Os2** (B), **Os3** (C), **PpIX** (D) and **Ce6** (E) in normoxia conditions in CT26 cells in the dark. 38
Figure S13. Fluorometric cell viability assay of complex **Os2** in normoxia conditions in CT26 cells upon excitation at 480 nm (A), 510 nm (B), 540 nm (C), 595 nm (D), 645 nm (E), 670 nm (F), 740 nm (G) and 770 nm (H)...39
Figure S14. Fluorometric cell viability assay of complex **Ce6** in normoxia conditions in CT26 cells upon excitation at 480 nm (A), 510 nm (B), 540 nm (C), 595 nm (D), 645 nm (E), 670 nm (F), 740 nm (G) and 770 nm (H)...40
Table S2. IC_{50} values in the dark and upon excitation at 740 nm (1.12 mW/cm^2, 60 min, 12.60 J/cm^2) and 770 nm (0.68 mW/cm^2, 120 min, 15.30 J/cm^2) for **Os2** and **Ce6** on human glioblastoma cells (U-87 MG)..41
Figure S15. Fluorometric cell viability assay of complex **Os2** and **Ce6** in normoxia conditions in U87-MG cells in the dark (A, D), upon 740 nm excitation (B, E) and 770 nm (C, F) excitation...42
Table S3. IC_{50} values in the dark for **Os2** and **Ce6** on mouse fibroblast cells (NIH/3T3) and human fibroblast cells (MRC-5). 43
Figure S16. Fluorometric cell viability assay of complex **Os2** and **Ce6** in normoxia conditions in NIH/3T3 cells (A-B) and MRC-5 (C-D) in the dark..44
Figure S17. Hypoxia cell incubator (A, Memmert CO_2 incubator) and hypoxia chamber (Plas labs 856-Series hypoxia chamber glove box) containing the irradiation setup (B)..............................45
Table S4. IC_{50} values in the dark and upon excitation at 740 nm (1.12 mW/cm^2, 60 min, 12.60 J/cm^2) for **Os2** and **Ce6** on mouse colon adenocarcinoma (CT-26) cells under hypoxia (1% O_2). 46
Figure S18. Fluorometric cell viability assay of complex **Os2** and **Ce6** in 1% O_2 hypoxia conditions in CT-26 cells upon 740 nm excitation (A-B) and in the dark (C-D)..47
Figure S19. Photostability in PBS solution with 10% FBS..48
Figure S20. Size distribution of **Os2**. 49
Figure S21. Confocal laser scanning microscopy images of trackers without **Os2**. 50

Figure S22. DCF levels in CT26 cells as assessed by flow cytometry..51
Figure S23. Time-dependent photogeneration of $^1O_2$ and •OH, as monitored by ABDA absorbance at 380 nm (A) and HPF fluorescence at 573 nm (B)..52
Figure S24. Cell viability of CT26 cells treated with **Os2** under irradiation or in the dark with or without ROS scavengers. 53
Figure S25. Gating strategies used for Annexin V/PI staining...54
Figure S26. Tumor growth curves of individual mice in the control group (A), **Cisplatin** group (B), **Ce6** group (C) and **Os2** group...55
Figure S27. Histopathological images of the main organs, containing heart, liver, spleen, lung, and kidney obtained from mice after different treatment..56
Figure S28. Gating strategies used for immune cells sorting in flow cytometry analysis.............57
Figure S29. (A-B) Representative flow cytometry analysis of M1-like TAMs (A) and M2-like TAMs (B). (C-D) The quantification of M1-like TAMs (C) and M2-like TAMs (D)....................58
Table S5. IC_{50} values upon excitation at 740 nm (1.12 mW/cm^2, 60 min, 12.60 J/cm^2) for **Os2**, **Ce6**, **PpIX** and **Hypericin** on mouse fibrosarcoma (MCA205) cells. 59
Figure S30. Detection of HMGB-1 release on MCA205 cells..60

Materials

Unless otherwise stated, common chemicals and solvents (HPLC-grade or reagent-grade quality) were purchased from commercial sources and used without further purification. When necessary, solvents were degassed by purging with dry, oxygen-free nitrogen for at least 15 min before use. Precursors 4-(1H-imidazo[4,5-f][1,10]phenanthrolin-2-yl)benzoic acid (compound **2**)[1], **Os(phen)$_2$Cl$_2$**[2] and **Os(bphen)$_2$ Cl$_2$**[2] were prepared according to previously reported procedures. Protoporphyrin IX (Catalog no. P8293) was purchased from Sigma-Aldrich. Oxaliplatin (Catalog no. 15335058) was purchased from Thermo Fisher Scientific. Cisplatin (Catalog no. D3371) was purchased from Tokyo Chemical Industry (TCI). Chlorin e6 (Catalog no. QC-6466) was purchased from Combi-Blocks. Hypericin (Catalog no. HY-N0453) was purchased from Medchem Express.

Instrumentation and methods of synthesis

All the reactions were performed under a nitrogen atmosphere using Schlenk glassware. A hot plate magnetic stirrer with an oil bath was used as the heating source in all reactions requiring heat. Thin layer chromatography (TLC) was performed using aluminum plates coated with a 0.2 mm-thick layer of silica gel 60 F254, and the detection of spots was achieved by exposure to 254 nm UV light. Flash column chromatography was carried out using silica gel 60 Å (70-230 mesh). Eluent mixtures are expressed as volume-to-volume (v/v) ratios. Proton (^{1}H) and proton-decoupled carbon ($^{13}C\{^1H\}$) NMR spectra were recorded at 25 °C on a Bruker Avance III HD 400 MHz or Bruker Avance Neo 500 MHz spectrometer using the signal of the deuterated

solvent as an internal deuterium lock. Chemical shifts (δ) are reported in ppm (parts per million) relative to the residual signal of the deuterated solvent. Coupling constants *J* are given in Hertz (Hz) and the multiplicity is expressed using the following abbreviations: s (singlet), d (doublet), dd (doublet of doublets), t (triplet), m (multiplet). ESI-HRMS experiments were carried out using an LTQ-Orbitrap XL from Thermo Scientific and operated in positive ionization mode, with a spray voltage at 3.6 kV. Sheath and auxiliary gas were set at a flow rate of 5 and 0 arbitrary units (a.u.), respectively. The voltages applied were 40 and 100 V for the ion transfer capillary and the tube lens, respectively. The ion transfer capillary was held at 275°C. Detection was achieved in the Orbitrap with a resolution set to 100,000 (at *m/z* 400) and a m/z range between 200-2000 in profile mode. Spectrum was analyzed using the acquisition software XCalibur 2.1. The automatic gain control (AGC) allowed the accumulation of up to 2.105 ions for FTMS scans. Maximum injection time was set to 300 ms, and 1 µscan was acquired. 5 µL was injected using a Thermo Finnigan Surveyor HPLC system with a continuous infusion of methanol at 100 µL min^{-1}. Reversed-phase high-performance liquid chromatography (HPLC) analyses were conducted with a Waters Acquity Arc HPLC System featuring a quaternary pump solvent delivery module, online degasser, autosampler, and a Waters 2998 photodiode array detector. For HPLC separation, an Acquity UPLC CSH C18 column (3.0 mm × 50 mm, 1.7 µm) from Waters was used. The mobile phase followed a linear gradient from 90:10 (v/v) A/B to 0:100 (v/v) A/B over 4.0 min at a flow rate of 1.0 mL/min (A: 0.1% formic acid in H_2O; B: 0.1% formic acid in CH_3CN). The injection volume was 1 µL. Control of the HPLC

instrument and processing of the chromatogram output, including annotation of retention times, integration of peaks, and calculation of peak areas, were performed using MassLynx V4.1 software.

Synthesis and characterization of the ligands and osmium polypyridyl complexes

Os(phen)$_2$Cl$_2$[2-3]

Ammonium hexachloroosmate (IV) (500 mg, 1.14 mmol) and 1,10-phenanthroline (410 mg, 2.28 mmol) were dissolved in ethylene glycol (5 mL) and purged with N_2 for 10 min. The suspension was heated to 120 ºC and stirred for 2 h under a N_2 atmosphere. After reaction completion, the mixture was cooled to 0 ºC and a 1.0 M aq. solution of NaS_2O_4 (10 mL) was added dropwise. The resulting mixture was further stirred for 1 h at room temperature before it was kept in the fridge for 24 h. The black precipitate was collected by filtration and washed with ice-cold water (2 x 50 mL) and Et_2O (2 x 50 mL) to obtain the desired product that was used without further purification (black powder, 600 mg, 85% yield).

^{1}H NMR (400 MHz, DMSO-d_6) δ 9.28 (dd, J = 4.8, 1.6 Hz, 4H), 8.99 (dd, J = 8.2, 1.6 Hz, 4H), 8.32 (s, 4H), 8.16 (dd, J = 8.2, 4.8 Hz, 4H).

HRMS (ESI+): calcd for $C_{24}H_{16}Cl_2N_4Os$ m/z $[M]^+$ 622.0367; found: 622.0342.

Os(bphen)$_2$Cl$_2$[2, 4]

Ammonium hexachloroosmate (IV) (200 mg, 455 µmol) and 4,7-diphenyl-1,10-phenanthroline (318 mg, 957 µmol) were dissolved in ethylene glycol (10 mL) and purged with N_2 for 10 min. The suspension was heated to 120 ºC and stirred for 2 h under a N_2 atmosphere. After reaction completion, the mixture was cooled to 0 ºC and

a 1.0 M aq. solution of NaS_2O_4 (10 mL) was added dropwise. The resulting mixture was further stirred for 1 h at room temperature before it was kept in the fridge for 48 h. The black precipitate was collected by filtration, thoroughly washed with ice-cold water (2 x 50 mL) and Et_2O (2 x 50 mL) and dried under vacuum. The crude was purified by silica flash column chromatography (0 to 4% MeOH in DCM) to afford the desired product as a dark purple powder (330 mg, 78% yield).

^{1}H NMR (400 MHz, CD_2Cl_2): δ 10.29 – 10.13 (m, 2H), 8.12 (d, J = 9.4 Hz, 2H), 8.01 (d, J = 9.4 Hz, 2H), 7.84 (d, J = 5.6 Hz, 4H), 7.80 – 7.73 (m, 4H), 7.67 (t, J = 7.7 Hz, 4H), 7.59 – 7.33 (m, 12H), 6.94 (d, J = 5.6 Hz, 2H).

HRMS (ESI+): calcd for $C_{48}H_{32}Cl_2N_4Os$ m/z $[M]^+$ 926.1619; found: 926.1599.

Lig1[3]

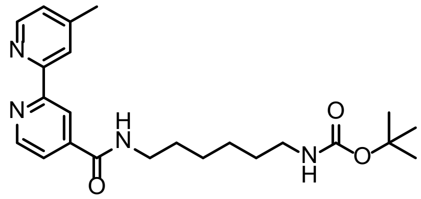

4'-methyl-[2,2'-bipyridine]-4-carboxylic acid (250 mg, 1.17 mmol), HATU (885 mg, 2.33 mmol) and DMAP (1.43 mg, 0.012 mmol) were dissolved in CH_2Cl_2 and stirred for 20 min. Then, *N*-Boc-1,6-hexanediamine (580 mg, 2.68 mmol) and triethylamine (0.5 mL, 3.50 mmol) were added, and the reaction was stirred overnight. The reaction mixture was washed with water (3 x 25 mL), dried with anhydrous Na_2SO_4 and evaporated to dryness. The crude was purified by silica flash column chromatography (50 to 100 % EtOAc in hexanes) to obtain **Lig1** as an off-white solid (320 mg, 78 % yield).

^{1}H NMR (400 MHz, $CDCl_3$) δ 8.80 (dd, J = 5.0, 0.9 Hz, 1H), 8.60 (s, 1H), 8.55 (dd, J = 5.0, 0.8 Hz, 1H), 8.29 – 8.24 (m, 1H), 7.78 (dd, J = 5.0, 1.7 Hz, 1H), 7.18 (ddd, J = 4.9, 1.7, 0.8 Hz, 1H), 6.63 (s, 1H), 4.55 (s, 1H), 3.48 (td, J = 7.1, 5.9 Hz, 2H), 3.19 – 3.07 (m, 2H), 2.46 (s, 3H), 1.70 – 1.58 (m, 2H), 1.56 – 1.46 (m, 2H), 1.46 – 1.27 (m, 13H). Data are in accordance with the literature. [3]

Os1(Boc)

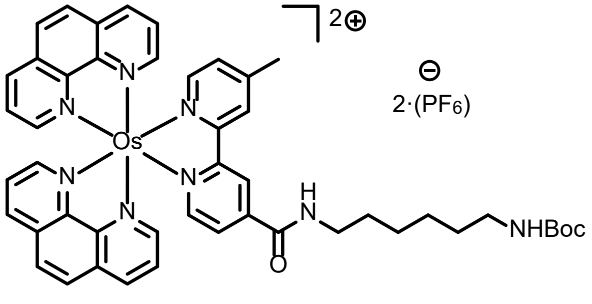

$Os(phen)_2Cl_2$ (450 mg, 724 μmol) and **Lig1** (300 mg, 724 μmol) were combined in degassed ethylene glycol (40 mL) and heated at 90 °C for 24 h under a N_2 atmosphere. The solution was cooled to room temperature, and a saturated aqueous solution of ammonium hexafluorophosphate (20 mL) was added to precipitate a dark solid. The solid was collected by filtration, washed with water (3 x 25 mL) and Et_2O (3 x 25 mL), and dried under vacuum overnight. The crude product was purified by silica flash column chromatography (0 to 20% MeOH in DCM) to yield **Os1(Boc)** as a dark brown solid (223 mg, 25% yield).

^{1}H NMR (400 MHz, CD_3CN) 8.77 (d, J = 2.2 Hz, 1H), 8.49 (s, 1H), 8.50 – 8.40 (m, 2H), 8.35 (d, J = 8.2 Hz, 2H), 8.29 – 8.19 (m, 4H), 8.11 (t, J = 4.9 Hz, 2H), 7.82 – 7.67 (m, 5H), 7.54 – 7.46 (m, 2H), 7.40 (t, J = 5.2 Hz, 2H), 7.08 (d, J = 4.6 Hz, 1H), 5.94 (s,

1H), 5.27 (s, 1H), 3.38 (q, *J* = 6.6 Hz, 2H), 3.01 (q, *J* = 6.5 Hz, 2H), 2.63 (s, 3H), 1.58 (p, *J* = 7.0 Hz, 2H), 1.48 – 1.24 (m, 15H).

HRMS (ESI+): calcd for $C_{47}H_{48}N_8O_3Os$ m/z $[M]^{2+}$ 482.1726, found: 482.1741.

Os1

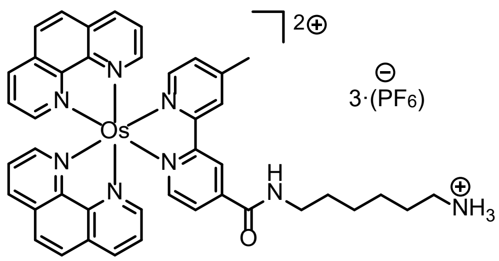

Os1(Boc) was dissolved in 1:1 CH_2Cl_2/TFA (10 mL) and stirred for 1 h at room temperature, followed by removal of the solvent *in vacuo*. The solid residue was taken up in acetone (20 mL) and a saturated aqueous solution of ammonium hexafluorophosphate (20 mL) was added. The resulting precipitate was collected by filtration, washed with water (3 x 25 mL) and Et_2O (3 x 25 mL), and dried under vacuum to yield **Os1** as a dark brown solid (24 mg, 84% yield).

^{1}H NMR (400 MHz, CD_3CN) δ 8.75 (s, 1H), 8.50 (s, 1H), 8.49 – 8.40 (t, *J* = 9.0 Hz, 2H), 8.34 (d, *J* = 8.1 Hz, 2H), 8.31 – 8.18 (m, 4H), 8.11 (t, *J* = 5.1 Hz, 2H), 7.79 (d, *J* = 4.8 Hz, 1H), 7.79 – 7.68 (m, 4H), 7.50 (ddd, *J* = 7.8, 5.4, 1.8 Hz, 2H), 7.49 – 7.41 (m, 1H), 7.41 (d, *J* = 5.9 Hz, 1H), 7.38 (dd, *J* = 6.0, 1.7 Hz, 1H), 7.08 (d, *J* = 6.8 Hz, 1H), 3.38 (q, *J* = 6.7 Hz, 2H), 2.99 – 2.91 (m, 2H), 2.63 (s, 3H), 1.61 (m, 4H), 1.41 – 1.33 (m, 4H).

^{13}C NMR: (101 MHz, CD_3CN) δ 164.1, 161.3, 159.4, 153.3, 153.2, 153.0, 152.9, 152.8,

151.7, 151.2, 151.0, 150.8, 150.4, 142.4, 137.6, 137.6, 132.3, 129.2, 127.2, 127.1, 126.4, 126.0, 122.6, 41.1, 40.6, 29.8, 27.5, 26.7, 26.3, 21.0.

HRMS (ESI+): calcd for $C_{42}H_{40}N_8OOs$ m/z $[M]^{2+}$ 432.1465, found: 432.1462.

Os2[3]

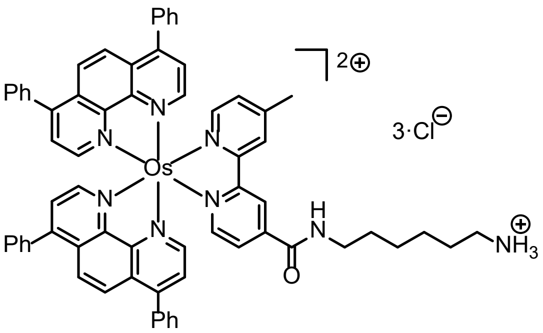

Os(bphen)$_2$Cl$_2$ (449 mg, 485 µmol) and **Lig1** (200 mg, 485 µmol) were combined in degassed ethylene glycol (30 mL) and heated at 110 °C for 24 h under a N_2 atmosphere. The solution was cooled to room temperature, and a saturated aqueous solution of ammonium hexafluorophosphate (20 mL) was added to induce the precipitation of a dark solid that was collected by filtration, washed with water (3 x 25 mL) and Et_2O (3 x 25 mL), and dried under vacuum. The crude was purified by silica flash column chromatography (0 to 10% 0.1 M aq. KNO_3 in CH_3CN) to yield a mixture of the *N*-Boc protected complex **Os2(Boc)** and the corresponding free amine **Os2** (~500 mg). The mixture was dissolved in 1:1 CH_2Cl_2/TFA (10 mL) and stirred for 2 h at room temperature, followed by removal of the solvent *in vacuo*. The solid residue was taken up in acetone (20 mL) and a saturated aqueous solution of ammonium hexafluorophosphate (10 mL) was added. The resulting precipitate was collected by filtration, washed with water (3 x 25 mL) and Et_2O (3 x 25 mL), and dried under

vacuum. The crude product was purified by silica flash column chromatography (0 to 10% 0.1 M aq. KNO_3 in CH_3CN) to yield **Os2** as a dark brown solid (PF_6^-/NO_3^- salt). In order to obtain the Cl^- salt, the previous solid was dissolved in 1:1 MeOH-H_2O (20 mL), mixed with Amberlite IRA-410 chloride form (4 g) and gently stirred overnight at room temperature. Finally, the resin was removed by filtration and the volatiles were concentrated *in vacuo* to obtain the desired product (356 mg, 59% yield).

^{1}H NMR (400 MHz, CD_3OD) δ 9.11 (d, J = 1.9 Hz, 1H), 8.72 (s, 1H), 8.38 – 8.23 (m, 6H), 8.10 (dd, J = 9.1, 5.7 Hz, 2H), 8.02 (d, J = 6.1 Hz, 1H), 7.81 (t, J = 5.5 Hz, 2H), 7.73 – 7.54 (m, 24H), 7.27 (d, J = 5.2 Hz, 1H), 3.46 (t, J = 7.2 Hz, 2H), 2.92 (t, J = 7.1 Hz, 2H), 2.73 (s, 3H), 1.73 – 1.57 (m, 4H), 1.49 – 1.40 (m, 4H).

HRMS (ESI+): calcd for $C_{66}H_{56}N_8OOs$ m/z $[M]^{2+}$ 584.2091; found 584.2098.

Lig2

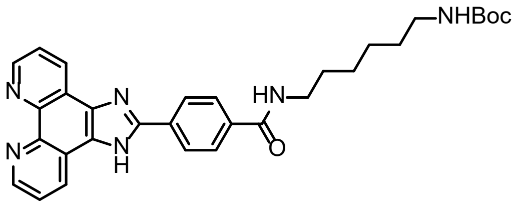

4-(1*H*-imidazo[4,5-f][1,10]phenanthrolin-2-yl)benzoic acid (compound **2**)[1](100 mg, 294 µmol), HATU (134 mg, 353 µmol) and DIPEA (153 µL, 881 µmol) were dissolved in anhydrous DMF (4 mL) under a N_2 atmosphere, and cooled to 0 ºC. After stirring for 15 min, *N*-Boc-1,6-hexanediamine (66 µL, 294 µmol) was added, and the reaction was allowed to proceed overnight at room temperature. The reaction mixture was evaporated to dryness to give the crude, which was purified by alumina (neutral) flash

column chromatography (0 to 20% MeOH in CH_2Cl_2) to obtain **Lig2** as a pale-orange solid (80 mg, 51% yield).

^{1}H NMR (400 MHz, CD_3OD) δ 8.88 (d, *J* = 2.9 Hz, 2H), 8.73 – 8.62 (m, 2H), 8.14 (d, *J* = 8.0 Hz, 2H), 7.96 (d, *J* = 8.0 Hz, 2H), 7.63 (dd, *J* = 8.1, 4.3 Hz, 2H), 3.43 (t, *J* = 7.2 Hz, 2H), 3.06 (t, *J* = 6.9 Hz, 2H), 1.68 (p, *J* = 7.1 Hz, 2H), 1.57 – 1.35 (m, 15H).

^{13}C NMR (101 MHz, CD_3OD) δ 169.1, 158.5, 150.8, 148.4, 143.8, 136.4, 133.1, 130.8, 128.7, 127.2, 124.1, 79.7, 49.0, 41.2, 41.0, 30.9, 30.4, 28.8, 27.8, 27.5.

Os3

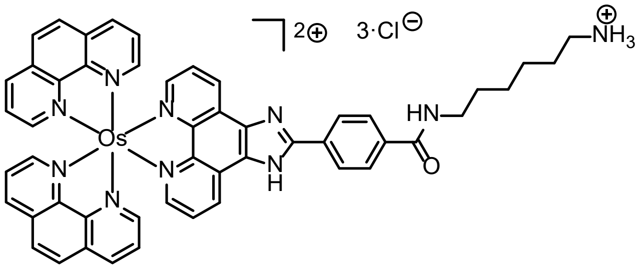

Os(phen)$_2$Cl$_2$ (81 mg, 130 μmol) and **Lig2** (70 mg, 130 μmol) were combined in degassed 1:1 EtOH:H_2O (20 mL) and refluxed for 24 h under a N_2 atmosphere. The solution was cooled to room temperature, and a saturated aqueous solution of ammonium hexafluorophosphate (10 mL) was added to induce the precipitation of a dark solid that was collected by filtration, washed with water (3 x 10 mL) and Et_2O (3 x 10 mL), and dried under vacuum. The solid was dissolved in 1:1 CH_2Cl_2/TFA (5 mL) and stirred for 2 h at room temperature, followed by removal of the solvent *in vacuo*. The solid residue was taken up in acetone (20 mL) and a saturated aqueous solution of ammonium hexafluorophosphate (10 mL) was added. The resulting precipitate was

collected by filtration, washed with water (3 x 10 mL) and Et_2O (3 x 10 mL), and dried under vacuum. The crude product was purified by silica flash column chromatography (0 to 20% 0.1 M aq. KNO_3 in CH_3CN) to yield **Os3** as a dark brown solid (PF_6^-/NO_3^- salt). In order to obtain the Cl^- salt, the previous solid was dissolved in 1:1 MeOH-H_2O (10 mL), mixed with Amberlite IRA-410 chloride form (2 g) and gently stirred overnight at room temperature. Finally, the resin was removed by filtration and the volatiles were concentrated *in vacuo* to obtain the desired product (30 mg, 23% yield).

^{1}H NMR (400 MHz, CD_3CN) δ 8.75 (d, J = 8.2 Hz, 2H), 8.38 (dd, J = 8.3, 1.1 Hz, 4H), 8.36 – 8.33 (m, 2H), 8.25 (s, 4H), 8.02 – 7.96 (m, 4H), 7.90 (ddd, J = 20.8, 5.4, 1.2 Hz, 4H), 7.64 – 7.47 (m, 6H), 7.28 (t, J = 5.8 Hz, 1H), 3.39 (q, J = 6.6 Hz, 2H), 3.00 – 2.93 (m, 2H), 1.73 – 1.50 (m, 4H), 1.50 – 1.35 (m, 4H).

^{13}C NMR (101 MHz, CD_3CN) δ 167.5, 153.5, 153.3, 153.1, 151.2, 151.1, 151.0, 149.4, 137.5, 132.8, 132.3, 131.1, 129.2, 128.9, 127.5, 127.0, 118.3, 40.9, 40.2, 30.0, 27.5, 26.6, 26.1, 1.3.

HRMS (ESI+): calcd for $C_{50}H_{42}N_{10}OOs$ m/z $[M]^{2+}$ 495.1574; found 495.1574.

Instrumentation and methods for photophysical property analysis

The absorption spectra were measured with a Shimadzu UV-3600 double-beam UV-visible spectrophotometer. The fluorescence spectra were measured with a Fluorolog FL3-222 spectrofluorimeter (Horiba Jobin Yvon, Palaiseau, France) equipped with a 450 W xenon arc lamp, with a thermostatically-controlled cell holder compartment (25°C), a UV-visible photomultiplier tube R928 (HAMAMATSU Japan) and an infrared detector InGaAs cooled by liquid nitrogen (DSS-IGA 020L Electro-Optical

System Inc, Phoenixville, PA, USA). The excitation beam is separated by a SPEX dual network monochromator (1200 lines / mm blazed at 330 nm). The fluorescence was measured by the UV-Visible detector via the SPEX dual network emission monochromator (1200 lines / mm blazed at 500 nm). Singlet oxygen production was measured with an infrared detector InGaAs (800 - 1550 nm) via the dual network emission monochromator SPEX (600 lines / mm blazed at 1 μm).

All spectra were measured using 4-sided quartz cells. The absorption values of the references and samples at the excitation wavelength were adjusted to 0.2. All emission spectra were normalized to the same absorbance for the purpose of comparison. $[Ru(bpy)_3]^{2+}$ in acetonitrile was chosen as standard for both fluorescence and singlet oxygen quantum yield determination. Fluorescence quantum yield of $[Ru(bpy)_3]^{2+}$ in acetonitrile is evaluated at 0.077.[5] Singlet oxygen quantum yield of $[Ru(bpy)_3]^{2+}$ in acetonitrile is evaluated at 0.77.[6]

Time-resolved experiments were performed using for excitation: a pulsed laser diode emitting at 407 nm (LDH-P-C-400M, FWHM < 70 ps, 1 MHz) coupled with a driver PDL 800-D (both PicoQuant GmbH, BERLIN, Germany) and for detection: an avalanche photodiode SPCM-AQR-15 (EG & G, VAUDREUIL, Canada) coupled with a 650 nm long-wave pass filter as detection system. The acquisition was performed by a PicoHarp 300 module with a 4 channels router PHR-800 (both PicoQuant GmbH, BERLIN, Germany). The fluorescence decays were recorded using the single photon counting method. Data were collected up to 1000 counts accumulated in the maximum channel and analyzed using Time Correlated Single Photon Counting (TCSPC)

software Fluofit (PicoQuant GmbH, BERLIN, Germany) based on iterative reconvolution using a Levensberg-Marquandt algoritm, enabling the obtention of multi-exponential profiles (mainly one or two exponentials in our cases).

Photostability

Compound **Os2** and **Ce6** were dissolved in biologically relevant media (PBS solution with 10% FBS) at 50 μM from the stock solution (10 mM in DMSO). The UV-Vis spectrum of each complex was recorded from 300 nm to 900 nm at 10- or 20-minutes intervals using BioTek Cytation 5 Cell Imaging Multimode Reader (Agilent Technologies).

DLS Analysis

The hydrodynamic diameter was determined using dynamic light scattering (DLS, Malvern ZetaSizer Nano ZS) at 25 °C. PBS with 10% FBS solutions were filtered through a 0.22 μM membrane before use. **Os2** was sonicated in either PBS with 10% FBS solution (20 μM) or PBS solution (20 μM) for 10 minutes before measurement. Three independent sample replicates were performed for each condition, and both the count rate and the polydisperse index were within the optimal detection range.

Cell culture

The murine colon carcinoma (CT-26) cells, luciferase-expressing CT26 (CT26-luc) cells, human fibroblast cells (MRC-5) human glioblastoma multiforme (U-87 MG) cells were cultured in DMEM medium with 10% fetal bovine serum (FBS), penicillin (100 U ml^{-1}), streptomycin (100 U ml^{-1}), and 1% L-glutamine. The murine fibroblast NIH/3T3 cells were cultured in DMEM medium with 10% calf bovine serum, penicillin

(100 U ml^{-1}), streptomycin (100 U ml^{-1}), and 1% L-glutamine. The murine fibrosarcoma (MCA205) cells were cultured in RPMI 1640 medium with 10% fetal bovine serum (FBS), penicillin (100 U ml^{-1}), streptomycin (100 U ml^{-1}), and 1% L-glutamine. All cells were incubated at 37 °C in a humidified atmosphere with 5 % CO_2.

Cell viability assay

Normoxia. The *in vitro* viability of cells exposed to the complexes was determined using a resazurin assay. Cells were seeded in 96-well plates at densities of 4,000 cells/well for CT26, MCA205 and U-87 MG cells, and 8,000 cells/well for MRC-5 and NIH/3T3 cells. After 24 h of growth at 37 °C with 5% CO_2, the medium was replaced with fresh medium containing various concentrations of the compounds. Following a 4-h incubation in the dark, the medium was refreshed, and the plates were exposed to irradiation using a LUMOS-BIO photoreactor (Atlas Photonics). Non-irradiated plates were similarly refreshed with medium and kept in the dark for the same duration. After a 40-h recovery period, a freshly prepared resazurin solution (0.2 mg/mL) was added to each well, and the plates were left in the dark for 4 h before analysis on a BioTek Cytation 5 Cell Imaging Multimode Reader (Agilent Technologies).

Hypoxia. Hypoxia cells were from well-grown normoxia cells and cultured for at least two weeks before use. The medium used for hypoxia experiments was kept at 37 °C with 5% CO_2 and 1% O_2 for 2 days prior to use. CT-26 cells were seeded in 96-well plates at a density of 3,000 cells/well and grown at 37 °C, 5% CO_2 and 1% O_2 for 48 h. The medium was replaced with fresh medium containing various concentrations of the compounds, and this step was operated within 15 min. Following a 4-h incubation in

the dark, the medium was refreshed, and the plates were kept at 37 °C, 5% CO_2, 1% O_2 for 30 min before illuminated using a LUMOS-BIO photoreactor (Atlas Photonics). Non-irradiated plates were similarly refreshed with medium and kept in the dark for the same duration. After a 40-h recovery period, a freshly prepared resazurin solution (0.2 mg/mL) was added to each well, and the plates were left in the dark for 4 h before analysis on a BioTek Cytation 5 Cell Imaging Multimode Reader (Agilent Technologies).

Cellular colocalization by confocal microscopy

CT26 cells were seeded in confocal dishes (VWR) at a density of 8×10^4 cells per dish and cultured for 24 h at 37 °C with 5% CO_2. Subsequently, cells were treated with a 20 μM solution of complex **Os2** in DMEM and incubated in the dark for 4 h. Mito-Tracker Green (Thermo Fisher Scientific Inc., Catalog no. M7514) was added 10 min before the end of the incubation time at a final concentration of 100 nM. Lyso-Tracker Green DND 26 (Thermo Fisher Scientific Inc., Catalog no. L7526) was added 60 min before the end of the incubation time at a final concentration of 75 nM. ER-Tracker Green (Thermo Fisher Scientific Inc., Catalog no. E34251) was added 30 min before the end of the incubation time at a final concentration of 1 μM. Golgi-Tracker Green (Thermo Fisher Scientific Inc., Catalog no. B22650) was added 60 min before the end of the incubation time at a final concentration of 4 μM, the cells were incubated at 37°C for 30 min and then at 4°C for 30 min. Cells were then washed with warm washing buffer (phenol red-free DMEM with 10 mM HEPES) and incubated with Hoechst 33342 (Biotium, Catalog no. 40046) solution at a final concentration of 10 μg/mL for 10 min.

Cells were then washed with warm washing buffer and imaged using a confocal laser scanning microscope (TCS SP8, Leica Microsystems) equipped with a x63/1.40 plan apochromat oil objective. The excitation/emission wavelengths were 405/420-450 nm (Hoechst), 488/500-550 nm (Trackers) and 488/700-790 nm (**Os2**). Images were analyzed using the Leica Application Suite X (LAS X) software. Pearson correlation coefficients were analyzed using the Fiji software.

ROS determination

ROS were determined using the 2',7'-dichlorofluorescein diacetate (DCFH-DA) reagent (Sigma-Aldrich). CT26 cells were seeded in 24-well plates at a density of 8×10^4 cells per well and cultured for 24 h at 37 °C with 5% CO_2. Cells were then co-treated with the determined IC_{50} of complex **Os2** and selective ROS scavengers for 1 h, including 10 mM sodium azide (NaN_3) for singlet oxygen ($^1O_2$), 100 mM D-mannitol (Mann) for hydroxyl radical (•OH), 10 mM sodium 4,5-dihydroxybenzene-1,3-disulfonate (tiron) for superoxide radical ($O_2^{\bullet-}$) and 10 mM sodium pyruvate (NaPyr) for hydrogen peroxide (H_2O_2). Cells were then exposed to irradiation at 740 nm for 1 h, in the presence of the ROS scavengers. After irradiation, cells were collected and incubated with 10 μM of DCFH-DA for 30 min at room temperature in the dark. Cells were then washed with PBS twice and analyzed by flow cytometry. Data analysis was performed with FlowJo software.

Cell viability assay with ROS scavengers

CT26 cells were seeded in 96-well plates at a density of 4,000 cells per well and cultured for 24 h at 37 °C with 5% CO_2. Cells were then treated with **Os2** at various

concentrations and incubated for 4 h in the dark. Following the 4 h dark incubation with **Os2**, wells containing the compound were replaced with the medium containing the selective ROS scavengers: 5 mM sodium azide (NaN_3) for singlet oxygen ($^1O_2$) or 50 mM D-mannitol for hydroxyl radicals (•OH). Wells without **Os2** were refreshed with fresh culture medium. The plates were then irradiated at 740 nm for 1 h using a LUMOS-BIO photoreactor (Atlas Photonics). After light irradiation, all wells were replaced with fresh culture medium for the subsequent 40-h recovery period. Cell viability was assessed using a 0.2 mg/mL resazurin solution incubated in the dark for 4 h, and fluorescence was measured on a BioTek Cytation 5 Cell Imaging Multimode Reader.

Singlet oxygen and hydroxyl radical generation kinetics of Os2 in solution

The generation of singlet oxygen ($^1O_2$) and hydroxyl radicals (•OH) by **Os2** in solution was evaluated using ABDA (100 μM) and HPF (5 μM) as probes, respectively. **Os2** (10 μM) was mixed with the probes in PBS and irradiated with 740 nm light for different times (0, 5, 10, 15, 20, 30, 40, 50 and 60 min). ABDA absorbance decay was monitored to track $^1O_2$ generation, while HPF fluorescence increase (λex = 490 nm; λem = 500-700 nm) was measured to track •OH formation. Control solutions without **Os2** were measured under the same conditions to account for background signals. The ROS generation kinetics were analyzed by plotting absorbance or fluorescence versus irradiation time.

Immunofluorescence staining of calreticulin

CT26 cells were seeded in Lab-Tek® Chambered Coverglass System (Thermo Fisher

Scientific, Catalog no.155411) at a density of 8,000 cells per well and cultured for 24 h at 37 °C with 5% CO_2. The cells were then treated with a solution of the compounds at a concentration corresponding to the IC_{50} for 4 h. After refreshing the medium, cells were subjected to irradiation and incubated for an additional 12 h. Subsequently, the cells were fixed with 4% paraformaldehyde and immunostained with Anti-Calreticulin antibody (Abcam, Catalog no. ab2907) diluted 1/1000 at 4 °C overnight. Cells were then washed three times with PBS, treated with Goat Anti-Rabbit IgG H&L (Alexa Fluor® 647) (Abcam, Catalog no. ab150079) diluted 1/1000 for 2 h at room temperature in the dark, washed in PBS and treated with 10 μg/mL Hoechst for 15 min. After a final wash, cells were analyzed by confocal microscopy as detailed above.

Immunofluorescence staining of HMGB-1

CT26 or MCA205 cells were seeded in Lab-Tek® Chambered Coverglass System (Thermo Fisher Scientific, Catalog no.155411) at a density of 8,000 cells per well and cultured for 24 h at 37 °C with 5% CO_2. The cells were then treated with a solution of the compounds at a concentration corresponding to the IC_{50} for 4 h. After refreshing the medium, cells were subjected to irradiation and incubated for an additional 12 h. Subsequently, the cells were fixed with 4% paraformaldehyde, permeabilized with 0.2% Triton X-100 and immunostained using anti-HMGB1 antibody [EPR3507] (Abcam, Catalog no. ab79823) diluted 1/1000 and Goat Anti-Rabbit IgG H&L (Alexa Fluor® 647) (Abcam, Catalog no. ab150079) diluted 1/1000.

Detection of extracellular ATP

CT26 cells were seeded in 12-well plates at a density of 1×10^5 cells per well and

incubated for 24 h. Cells were then treated with the determined IC_{50} of complexes and irradiated or kept in the dark. Supernatants were collected 12 h post-treatment, and the extracellular ATP content was measured with Adenosine 5′-triphosphate (ATP) Bioluminescent Assay Kit (Sigma-Aldrich) according to manufacturer's instruction.

Cell viability assay for *in vivo* by flow cytometry

CT26-luc or MCA205 cells were seeded in 6-well plates at a density of 1×10^5 cells per well and cultured for 24 h at 37 °C with 5% CO_2. The medium was replaced by various concentrations of the compound dilutions in fresh medium. For **Ce6** and **Os2**, after 4 h incubation in the dark, the medium was refreshed and the plates were illuminated with a LUMOS-BIO photoreactor (Atlas Photonics), followed by an additional 20-h incubation in the dark. For **Cisplatin** and **Oxaliplatin**, cells were exposed to compound dilutions in the dark for 24 h then stained with annexin V (BD Biosciences, Catalog no. 556419) and PI (BD Biosciences, Catalog no.51-66211E). CT26-luc cells were analyzed by flow cytometry (Beckman Coulter Life Sciences, CytoFLEX LX Flow Cytometer). MCA205 cells were analyzed by flow cytometry (Thermo Fisher Scientific, Attune CytPix Flow Cytometer).

Animals

Female BALB/c mice and C57BL/6 mice (6 weeks old) were purchased from the JANVIER LABS (France). All animals were bred in a pathogen-free facility with a 12 h light/dark cycle at constant temperature and humidity with free access to food and water. All animals were kept 1-2 weeks after receipt before experiments.

***In vivo* vaccination study on CT26 cells**

The BALB/c female mice were randomly divided into 4 groups with each group containing 5 mice, which were named as Control group, **Cisplatin** group, **Ce6** group and **Os2** group. CT26-luc cells were treated *in vitro* with 600 μM **Cisplatin** in the dark, 6.5 μM **Ce6** or 1.8 μM **Os2** upon 740 nm LED light irradiation (60 min, 12.6 J cm^{-2}) following the methods described above. Subsequently, treated CT26-luc cells (3×10^5 cells in 100 μL sterile PBS) or 100 μL sterile PBS were injected subcutaneously into the left flank of each mouse. One week later, mice were rechallenged by injection with untreated CT26-luc cells (1×10^6 cells in 100 μL sterile PBS). The mice were monitored by an *in vivo* imaging system (Biospace Lab, Photon Imager Optima) and the tumor volume was calculated with the following equation: tumor volume = width^2 × length × 0.5.

***In vivo* vaccination study on MCA205 cells**

The C57BL/6 mice were randomly divided into 5 groups with each group containing 10 mice, which were named as Control group, **Cisplatin** group, **Oxaliplatin** group, **Ce6** group and **Os2** group. MCA205 cells were treated *in vitro* with 500 μM **Cisplatin** or 1400 μM **Oxaliplatin** in the dark, 5.5 μM **Ce6** or 3 μM **Os2** upon 740 nm LED light irradiation (60 min, 12.6 J cm^{-2}) following the methods described above. Subsequently, treated MCA205 cells (3×10^5 cells in 100 μL sterile PBS) or 100 μL sterile PBS were injected subcutaneously into the left flank of each mouse. One week later, mice were rechallenged by injection with untreated MCA205 cells (1×10^6 cells in 100 μL sterile PBS). The tumor volume was calculated with the following equation: tumor volume = width^2 × length × 0.5.

Biosafety assay *in vivo*

The BALB/c female mice were randomly divided into 4 groups which were named as Control group, **Cisplatin** group, **Ce6** group and **Os2** group. CT26-luc cells were treated *in vitro* with 600 μM **Cisplatin** in the dark, 6.5 μM **Ce6** or 1.8 μM **Os2** upon 740 nm LED light irradiation (60 min, 12.6 J cm^{-2}) following the methods described above. Subsequently, treated CT26-luc cells (3×10^5 cells in 100 μl sterile PBS) or 100 μl sterile PBS were injected subcutaneously into the left flank of each mouse. One week later, all the major organs, including heart, liver, lung, spleen, and kidney, were collected and examined by H&E staining.

Flow cytometry analysis of immune cells *in vivo*

The BALB/c female mice were randomly divided into 4 groups with each group containing 3 mice, which were named as Control group, **Cisplatin** group, **Ce6** group and **Os2** group. CT26-luc cells were treated *in vitro* with 600 μM **Cisplatin** in the dark, 6.5 μM **Ce6** or 1.8 μM **Os2** upon 740 nm LED light irradiation (60 min, 12.6 J cm^{-2}) following the methods described above. Subsequently, treated CT26-luc cells (3×10^5 cells in 100 μl sterile PBS) or 100 μl sterile PBS were injected subcutaneously into the left flank of each mouse. One week later, the mice were sacrificed, spleens were excised, mashed, and pressed. The cells were collected by filtering through a 70 μm strainer and centrifuged at 600 × g for 5 min at 4 °C. Cells from spleens were then lysed with red cells lysis solution (Thermo Fisher Scientific Inc., Catalog no. 00-4300-54). For surface marker analysis, the cells were stained with fluorescence-labeled anti-CD45-AF532 (Thermo Fisher Scientific Inc., Catalog no. 58-0451-80, clone 30-F11), anti-CD3-

PerCp Cy5.5 (Biolegend, Catalog no.100217, clone 17A2), anti-CD4- Kiravia 520 (Biolegend, Catalog no. 100477, clone GK1.5), anti-CD8a-BUV496 (Thermo Fisher Scientific Inc., Catalog no. 364-0081-80, clone 53-6.7), anti-CD25-BV785 (Biolegend, Catalog no. 102051, clone PC61), anti-CD69-PE/Cy7 (Biolegend, Catalog no. 104511, clone H1.2F3) , anti-CD11b-BUV 395 (Thermo Fisher Scientific Inc., Catalog no. 363-0112-80, clone M1/70), anti-CD80-PE/Fire 640 (Biolegend, Catalog no. 104759, clone 16-10A1), anti-F4/80-BV711 (Biolegend, Catalog no. 123147, clone BM8) and anti-CD206-PE/Dazzle 594 (Biolegend, Catalog no. 141731, clone C068C2). For transcription factor staining, the cells were treated with True-Nuclear™ Transcription Factor Buffer Set (Biolegend, Catalog no. 424401) after surface staining and re-stained with fluorescence-labeled anti-IFNγ-PE (Biolegend, Catalog no. 505807, clone XMG1.2), anti-FoxP3-BV421 (Biolegend, Catalog no.126419, clone MF-14) and Zombie NIR™ Fixable Viability Kit (Biolegend, Catalog no. 423105).

Cytokine assay

Serum samples were obtained from the mice on day 7 after treatment. Cytokine was detected using ELISA kits according to the manufacturer's instructions: IFN-γ (Biolegend, Catalog no. 430807) and IL-17A (Biolegend, Catalog no. 432507).

Statistical analysis

All the analysis data are given as mean ± SEM. Statistical analysis was performed using GraphPad Prism 8.0 software. *$P < 0.05$ was considered significant, **$P < 0.01$ and ***$P < 0.001$ were highly significant compared to corresponding control. For comparison of multiple groups, one-way analysis of variance (ANOVA) was used,

followed by Tukey's honest significant difference post hoc test. Comparison between the two groups was performed using an unpaired two tailed Student's t test.

Ethics statement

This study was performed with the approval of the Experimental Animal Manage Committee of Université Paris Cité, Faculté de Pharmacie (Approval No. 44203-2023062712522391 v5).

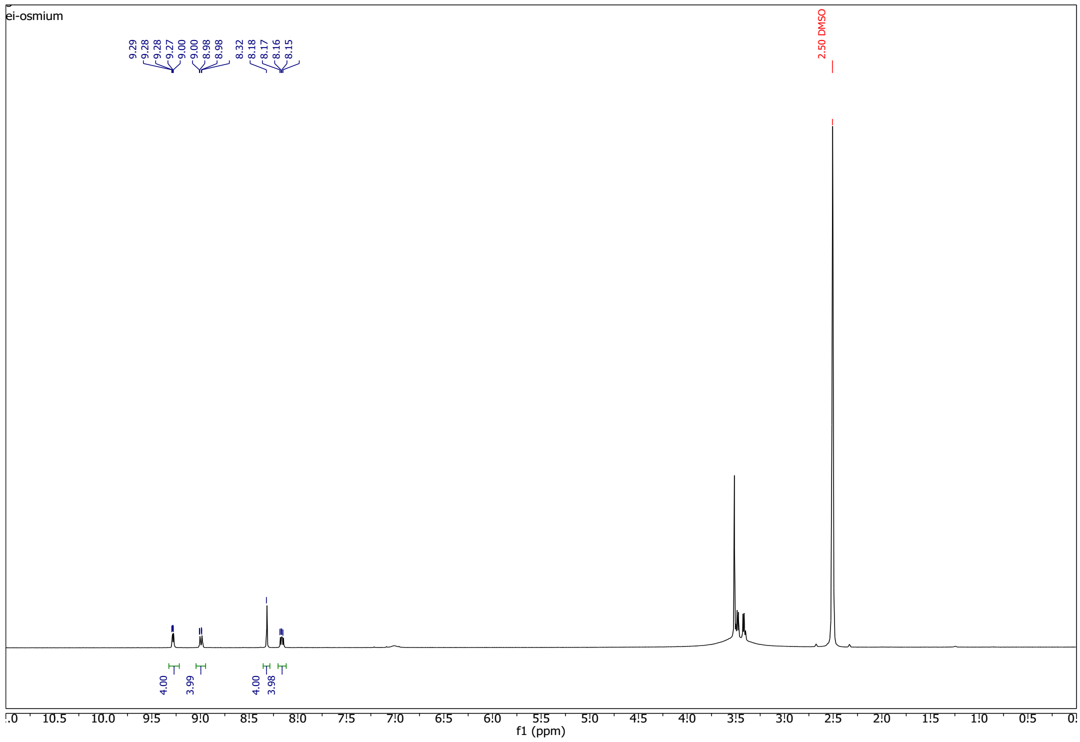

Figure S1. ^{1}H NMR spectrum of precursor **Os(phen)$_2$Cl$_2$** in d_6-DMSO.

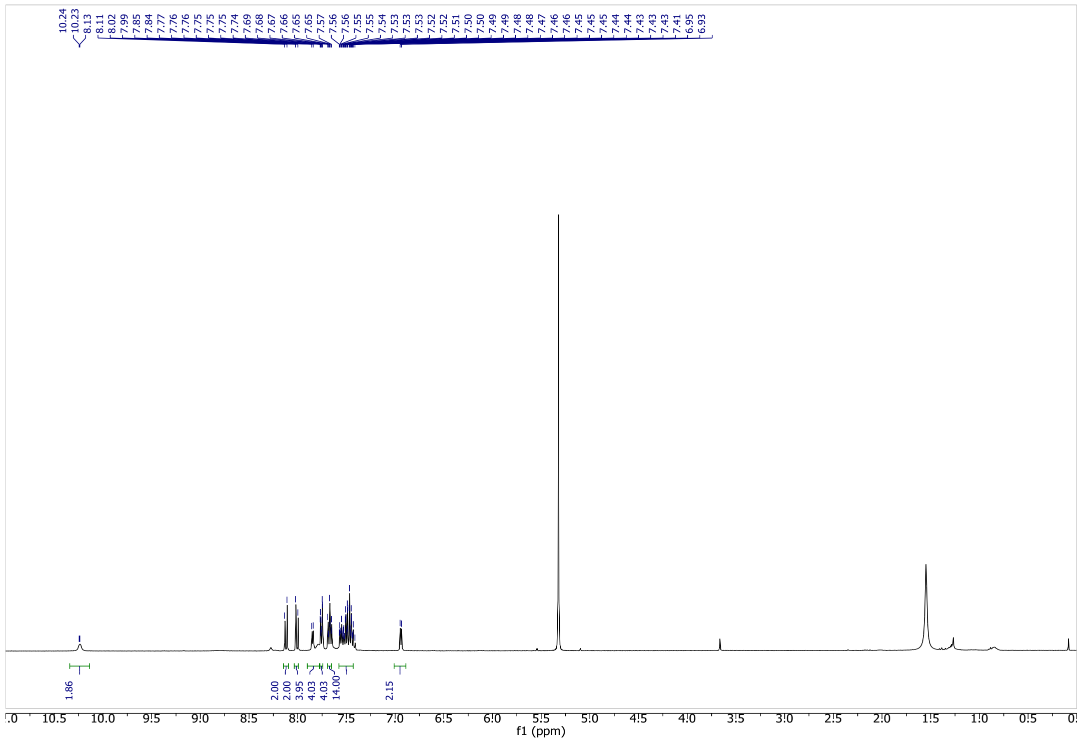

Figure S2. ^{1}H NMR spectrum of precursor **$Os(bphen)_2Cl_2$** in CD_2Cl_2.

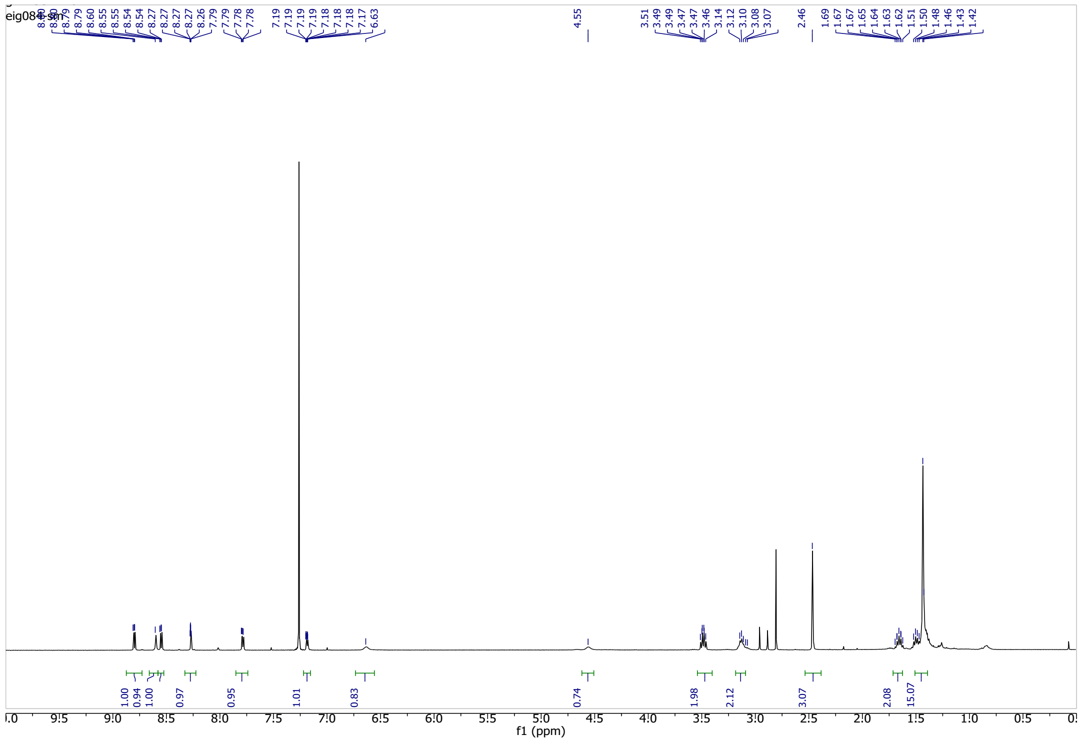

Figure S3. ^{1}H NMR spectrum of ligand **Lig1** in $CDCl_3$.

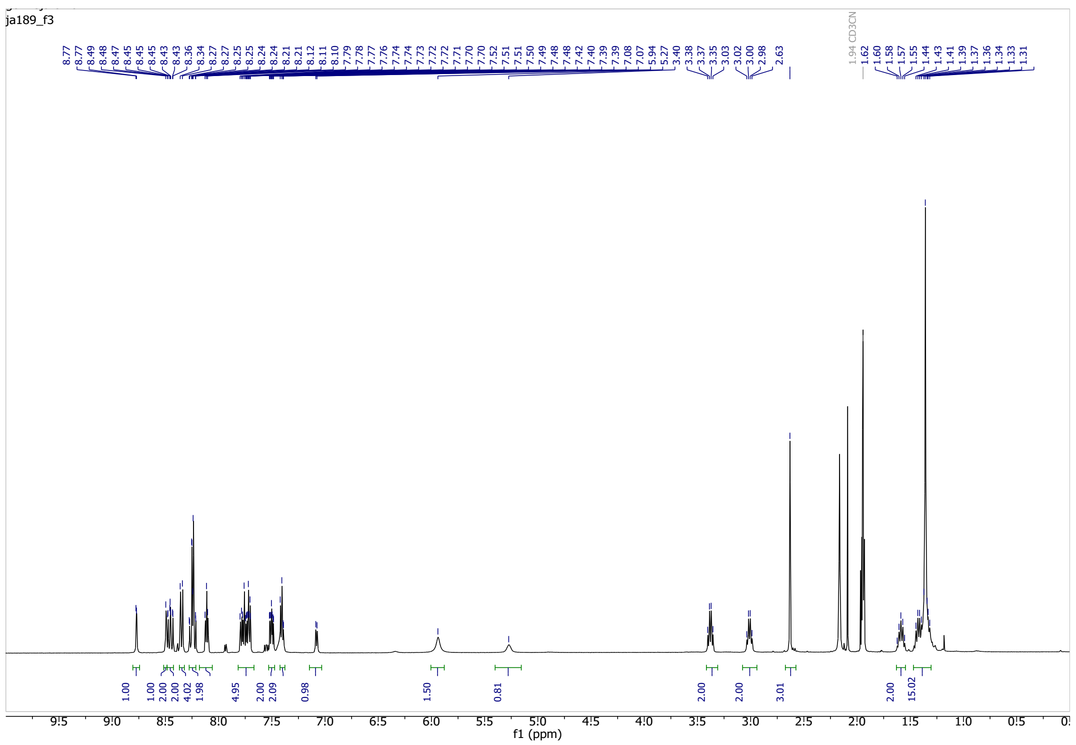

Figure S4. ^1H NMR spectrum of complex **Os1(Boc)** in CD_3CN.

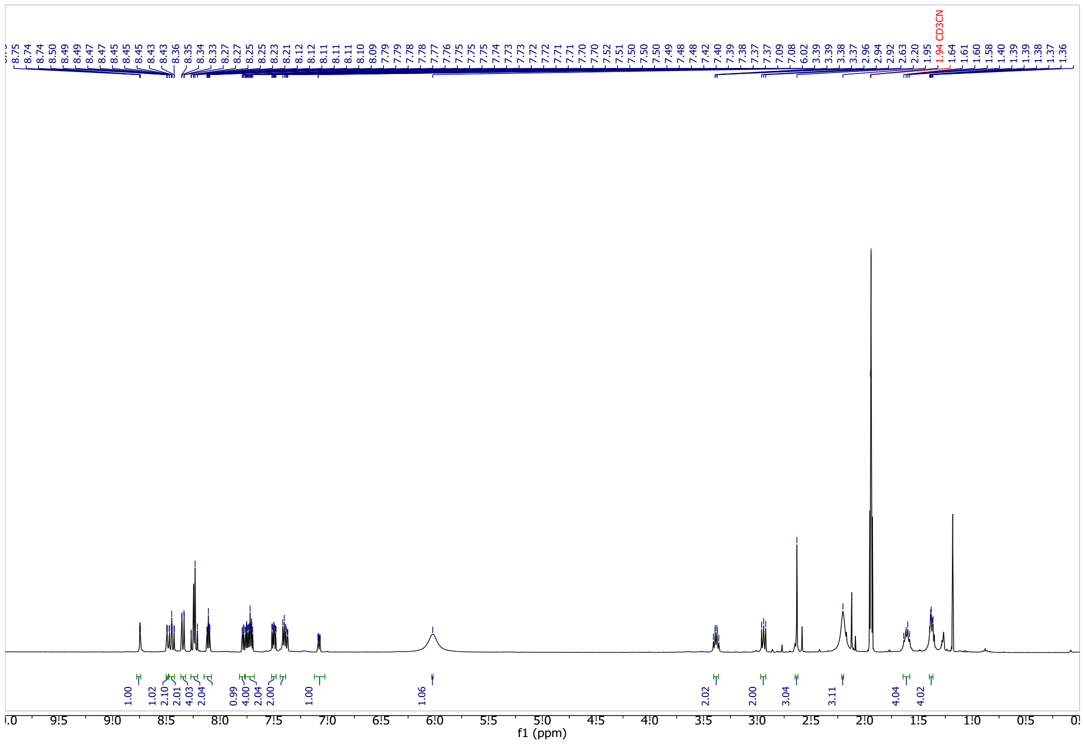

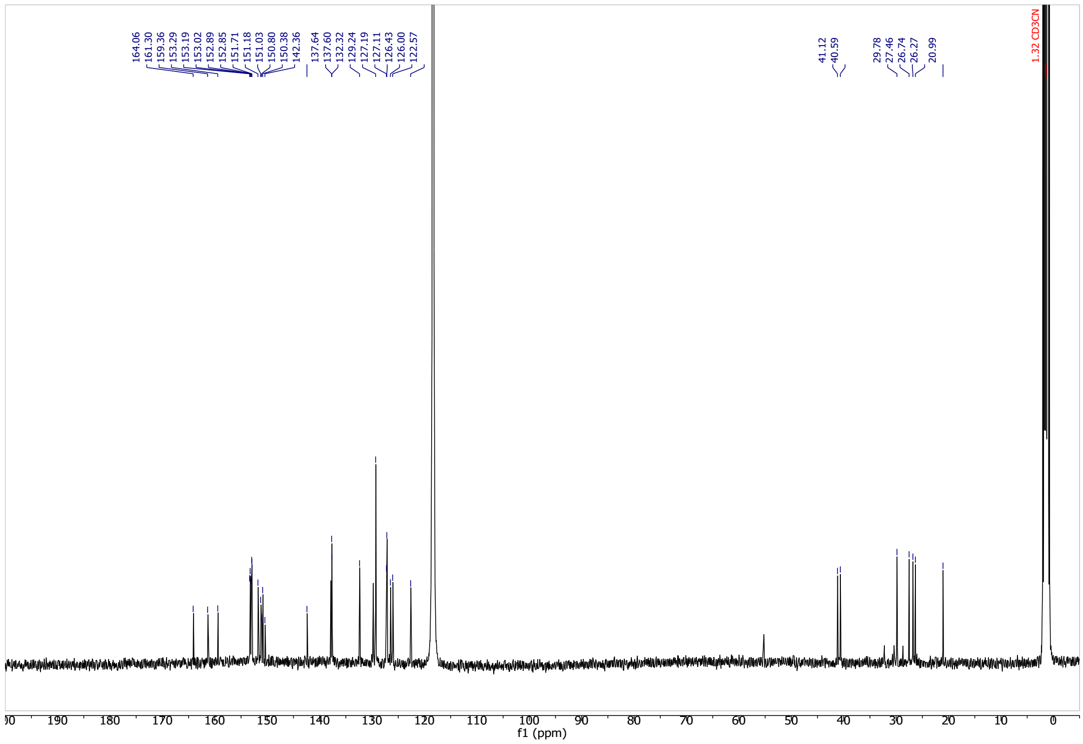

Figure S5. ^{1}H NMR (top) and ^{13}C NMR (bottom) spectrum of complex **Os1** in CD_3CN.

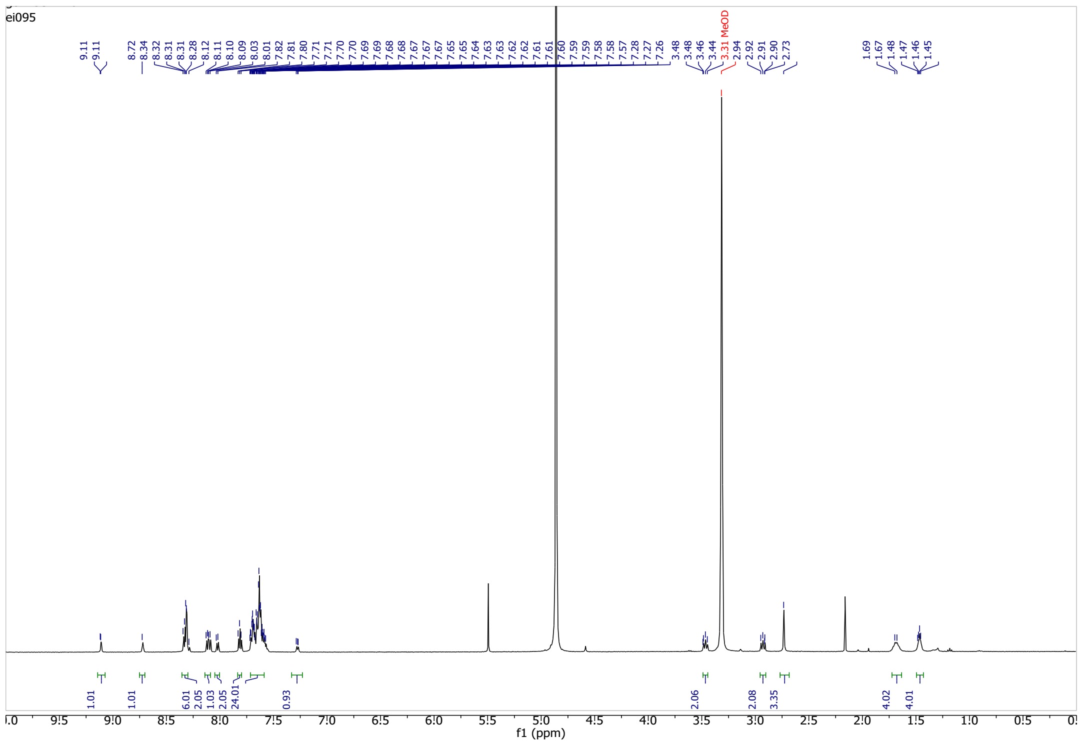

Figure S6. ^{1}H NMR spectrum of complex **Os2** in CD_3OD.

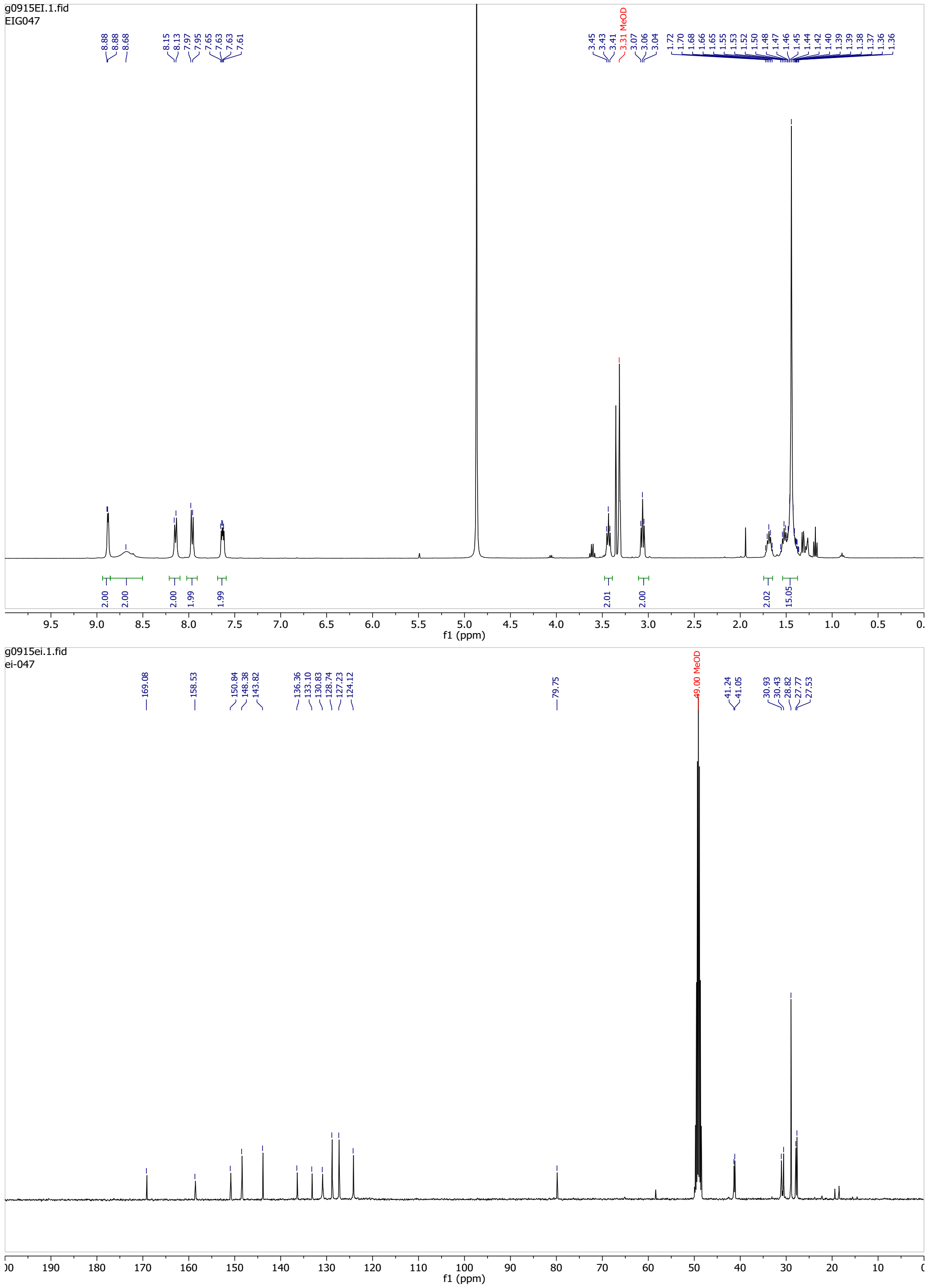

Figure S7. ^{1}H NMR (top) and ^{13}C NMR (bottom) spectra of ligand **Lig2** in CD_3OD.

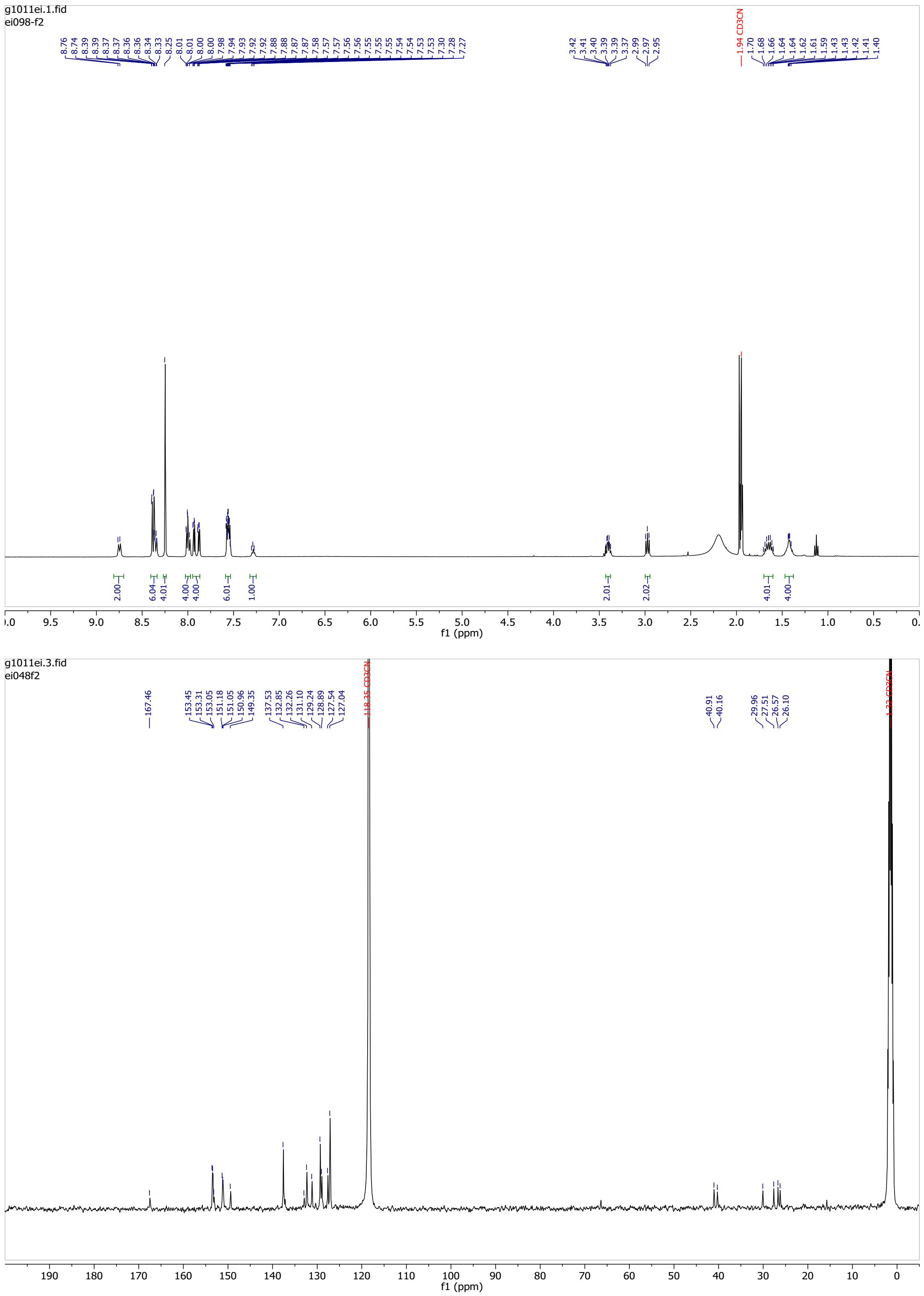

Figure S8. ^{1}H NMR (top) and ^{13}C NMR (bottom) spectra of complex **Os3** in CD_3CN.

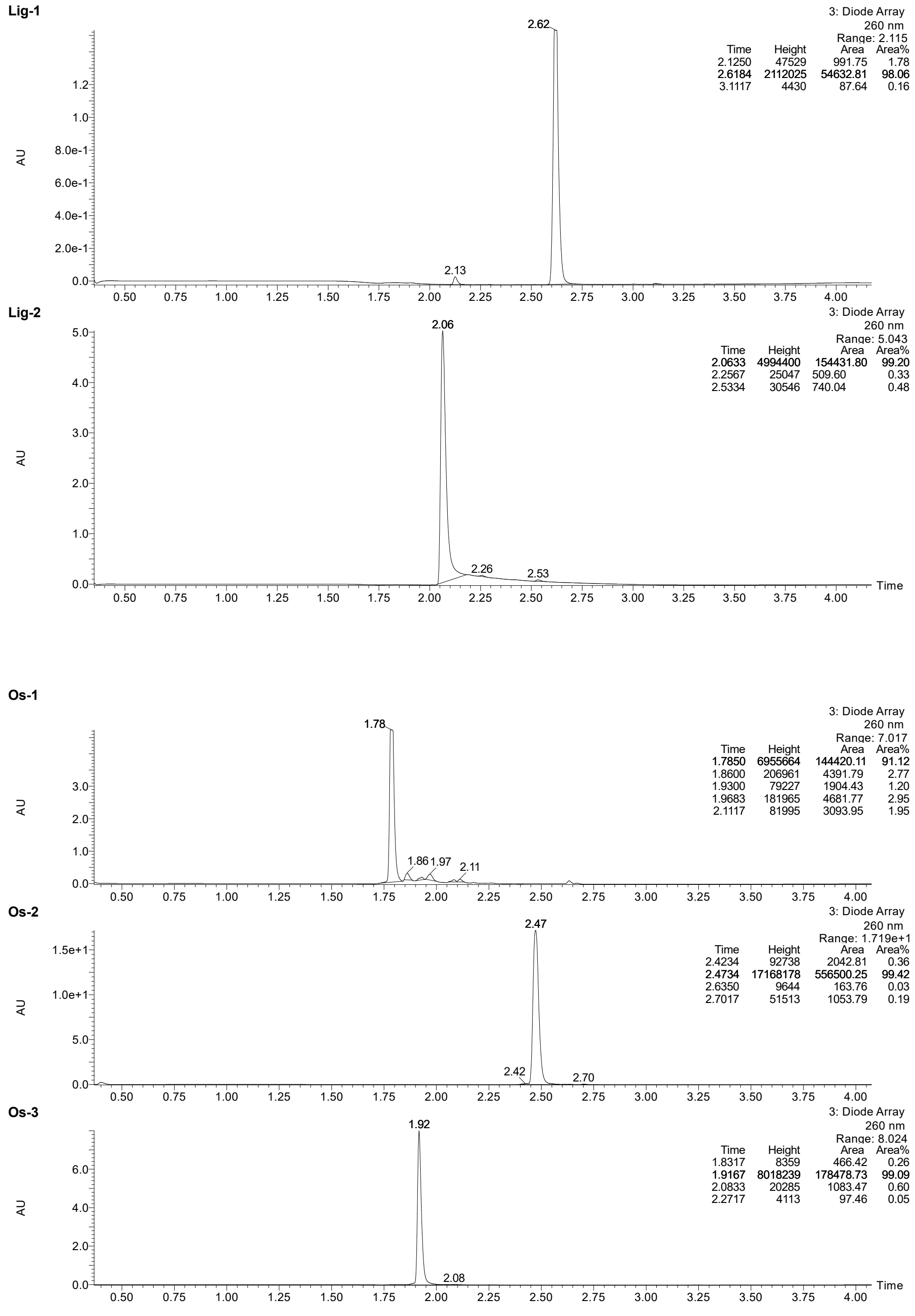

Figure S9. HPLC chromatograms of ligands **Lig1–2** (top) and Os(II) complexes **Os1–3** (bottom), recorded at 260 nm. Integration of the main peaks indicates a purity higher than 90% for all compounds.

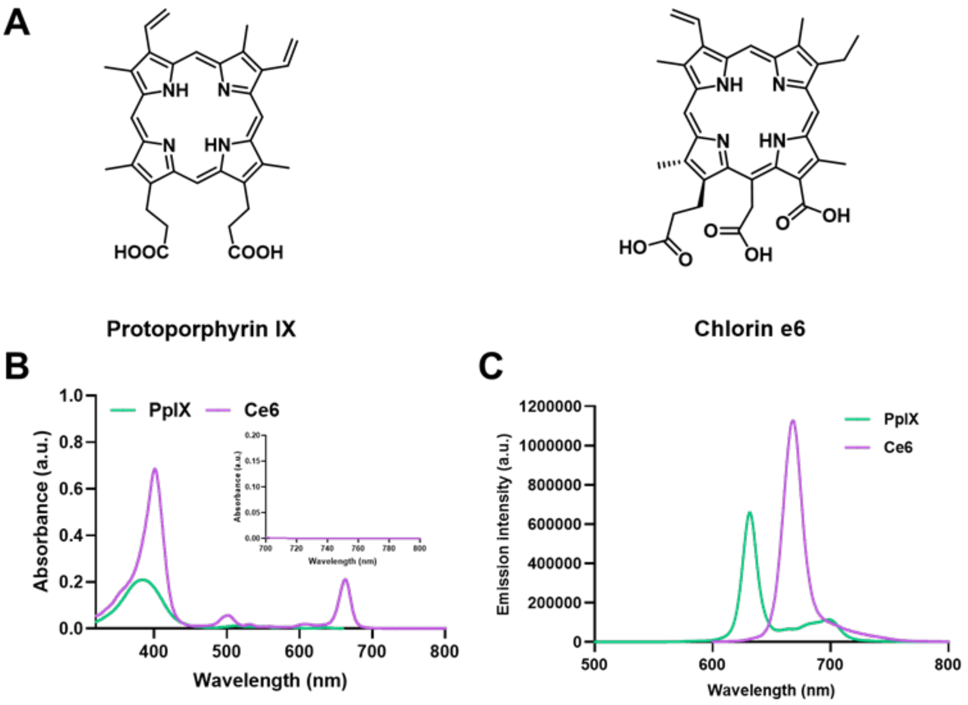

Figure S10. Chemical structures (A), UV/Vis absorption spectrum (B) and luminescence emission spectra (λex = 420 nm) (C) in ethanol of Protoporphyrin IX (**PpIX**) and Chlorin e6 (**Ce6**).

Table S1. Photophysical properties of **Os1-3** excited at 450 nm in acetonitrile, **Ce6** and **PpIX** in ethanol. The solvent was chosen based on its ability to provide optimal solubility for each respective compound.

Compound	Fluorescence quantum yield	Fluorescence lifetime (ns)	Singlet oxygen quantum yield
**Os1**	< 0.01	42.6	0.11
**Os2**	0.01	46.1	0.16
**Os3**	0.01	59.0	0.69
**Ce6**	0.20	4.7	0.65
**PpIX**	0.05	12.1	0.77

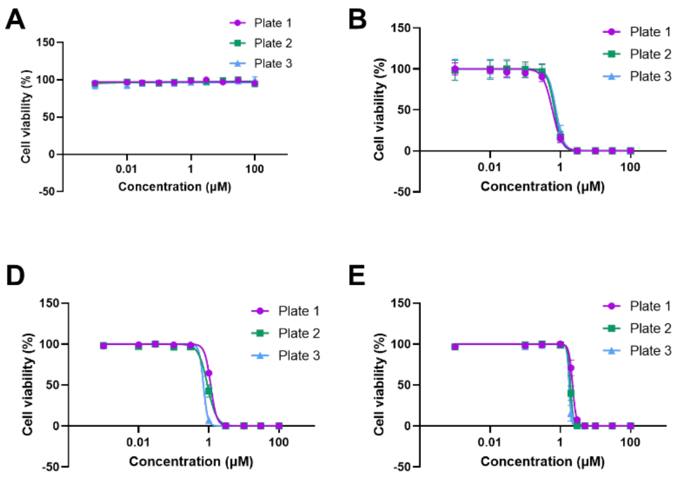

Figure S11. Fluorometric cell viability assay of complex **Os1** (A), **Os2** (B), **Os3** (C), **PpIX** (D) and **Ce6** (E) in normoxia conditions in CT26 cells upon illumination at 620 nm.

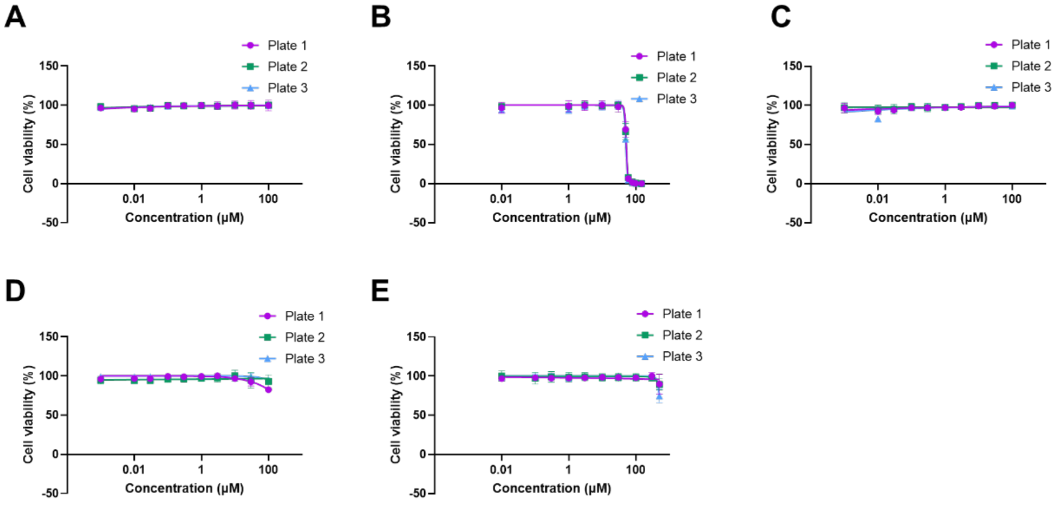

Figure S12. Fluorometric cell viability assay of complex **Os1** (A), **Os2** (B), **Os3** (C), **PpIX** (D) and **Ce6** (E) in normoxia conditions in CT26 cells in the dark.

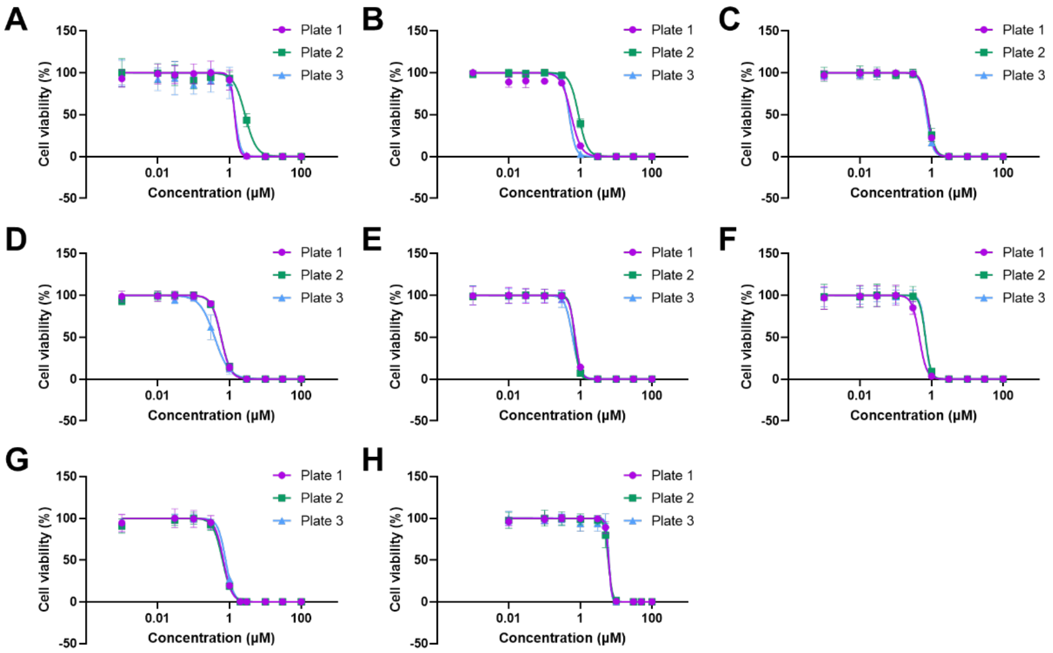

Figure S13. Fluorometric cell viability assay of complex **Os2** in normoxia conditions in CT26 cells upon excitation at 480 nm (A), 510 nm (B), 540 nm (C), 595 nm (D), 645 nm (E), 670 nm (F), 740 nm (G) and 770 nm (H).

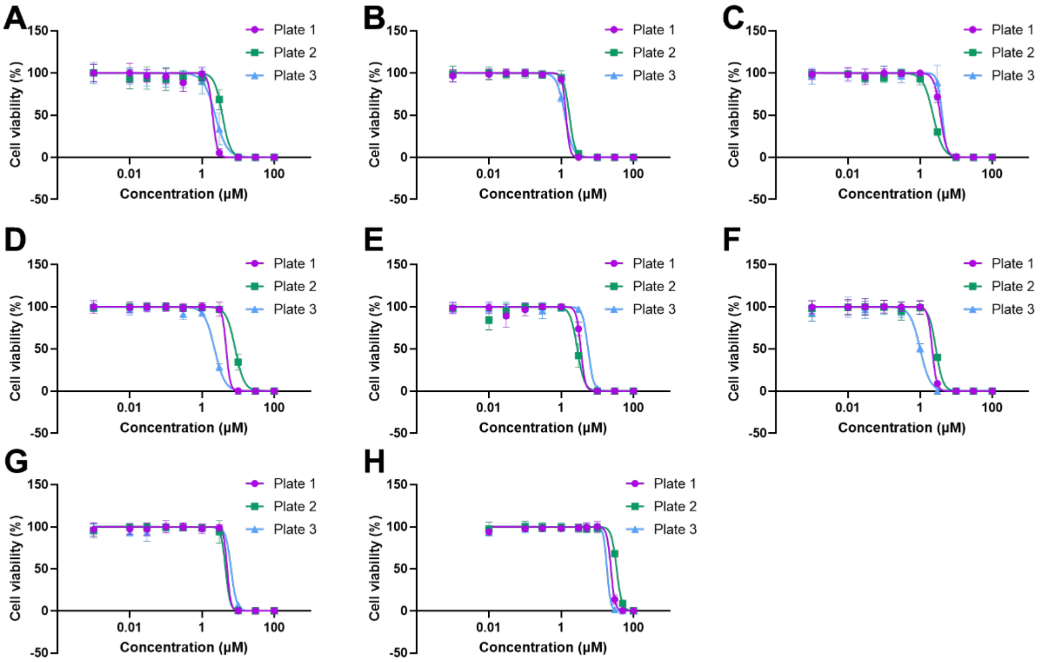

Figure S14. Fluorometric cell viability assay of complex **Ce6** in normoxia conditions in CT26 cells upon excitation at 480 nm (A), 510 nm (B), 540 nm (C), 595 nm (D), 645 nm (E), 670 nm (F), 740 nm (G) and 770 nm (H).

Table S2. IC_{50} values in the dark and upon excitation at 740 nm (1.12 mW/cm^2, 60 min, 12.60 J/cm^2) and 770 nm (0.68 mW/cm^2, 120 min, 15.30 J/cm^2) for **Os2** and **Ce6** on human glioblastoma cells (U-87 MG). Data are expressed as the mean ± SEM of three independent measurements. PI: phototoxicity index, $IC_{50}^{dark}/IC_{50}^{light}$.

U-87 MG	Dark (μM)	740 nm (μM)	PI	770 nm (μM)	PI
**Os2**	20.15 ± 0.09	0.44 ± 0.02	46	3.38 ± 0.60	6
**Ce6**	116.00 ± 6.99	4.15 ± 0.06	28	14.02 ± 3.14	8

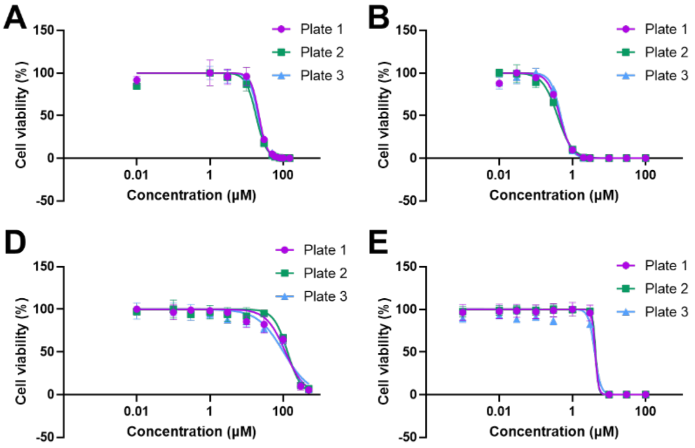

Figure S15. Fluorometric cell viability assay of complex **Os2** and **Ce6** in normoxia conditions in U87-MG cells in the dark (A, D), upon 740 nm excitation (B, E) and 770 nm (C, F) excitation.

Table S3. IC_{50} values in the dark for **Os2** and **Ce6** on mouse fibroblast cells (NIH/3T3) and human fibroblast cells (MRC-5). Data are expressed as the mean ± SEM of three independent measurements.

	NIH/3T3	MRC-5
**Os2**	> 100 μM	> 100 μM
**Ce6**	> 100 μM	> 100 μM

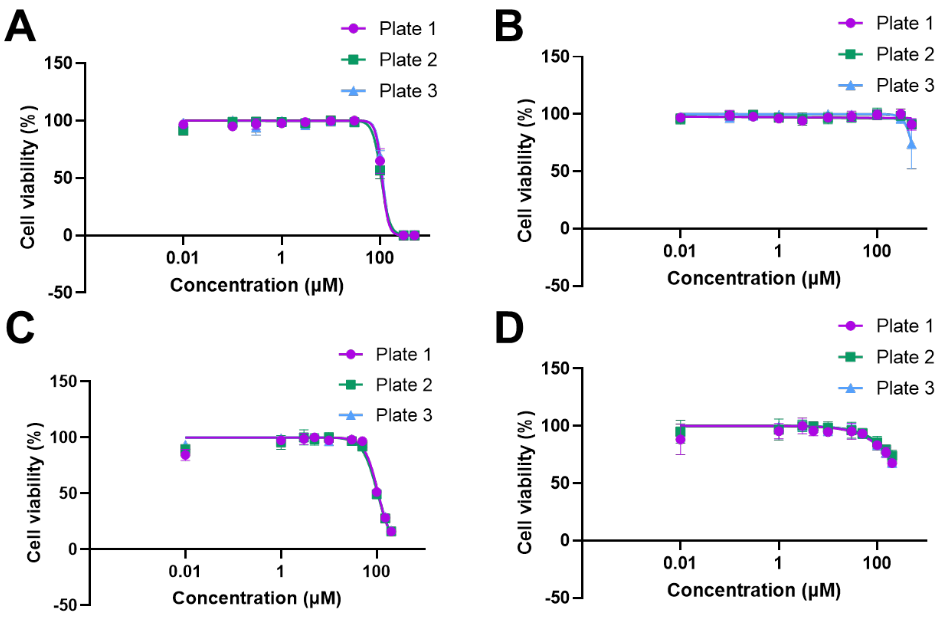

Figure S16. Fluorometric cell viability assay of complex **Os2** and **Ce6** in normoxia conditions in NIH/3T3 cells (A-B) and MRC-5 (C-D) in the dark.

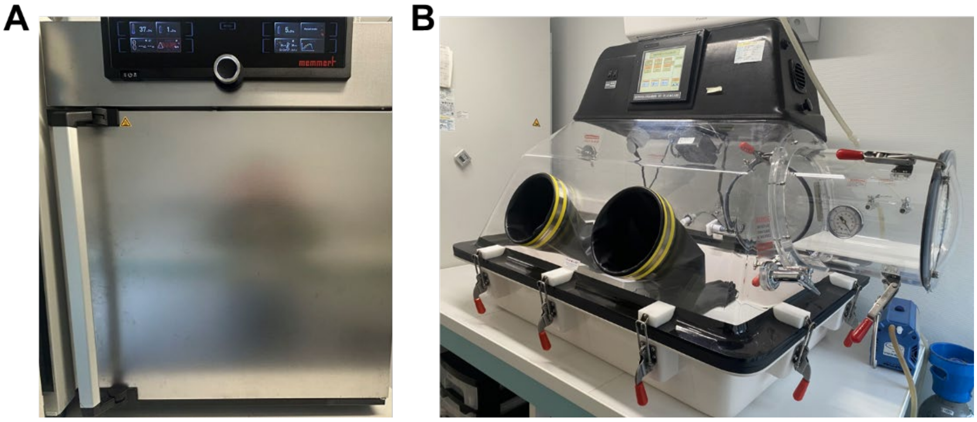

Figure S17. Hypoxia cell incubator (A, Memmert CO_2 incubator) and hypoxia chamber (Plas labs 856-Series hypoxia chamber glove box) containing the irradiation setup (B).

Table S4. IC_{50} values in the dark and upon excitation at 740 nm (1.12 mW/cm^2, 60 min, 12.60 J/cm^2) for **Os2** and **Ce6** on mouse colon adenocarcinoma (CT-26) cells under hypoxia (1% O_2). Data are expressed as the mean ± SEM of three independent measurements. PI: phototoxicity index, $IC_{50}^{dark}/IC_{50}^{light}$.

	Dark (μM)	740 nm (μM)	PI
**Os2**	50.73 ± 3.43	2.16 ± 0.15	46
**Ce6**	> 100	5.63 ± 0.41	28

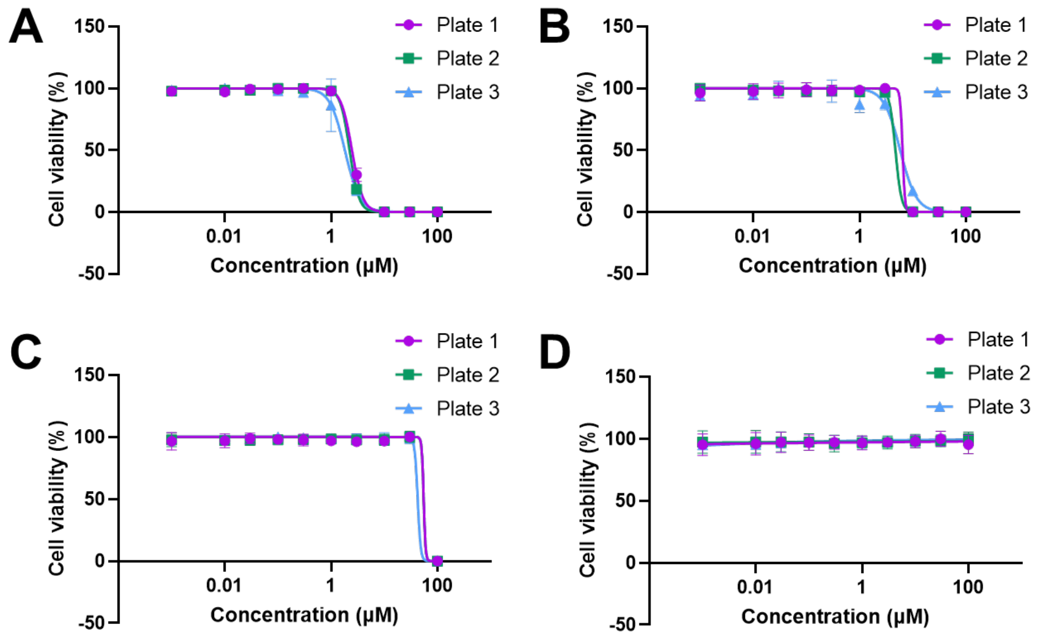

Figure S18. Fluorometric cell viability assay of complex **Os2** and **Ce6** in 1% O_2 hypoxia conditions in CT-26 cells upon 740 nm excitation (A-B) and in the dark (C-D).

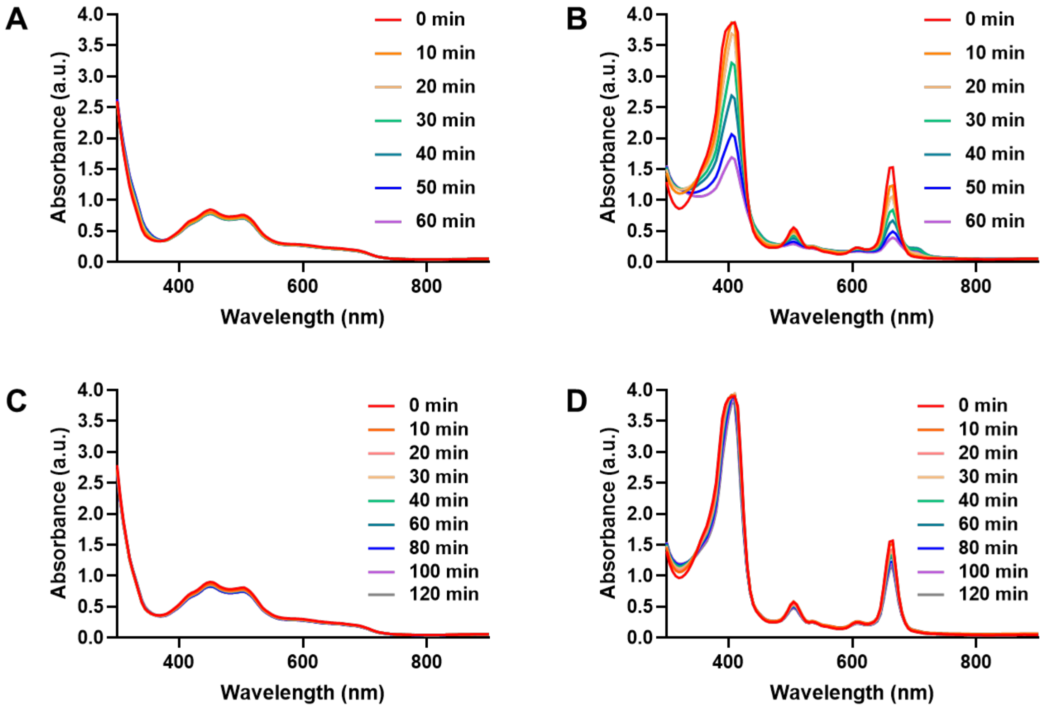

Figure S19. Photostability in PBS solution with 10% FBS. (A-B) Compound **Os2** and **Ce6** under 740 nm excitation (12.60 J/cm^2, 1 h). (C-D) Compound **Os2** and **Ce6** under 770 nm excitation (15.30 J/cm^2, 2 h).

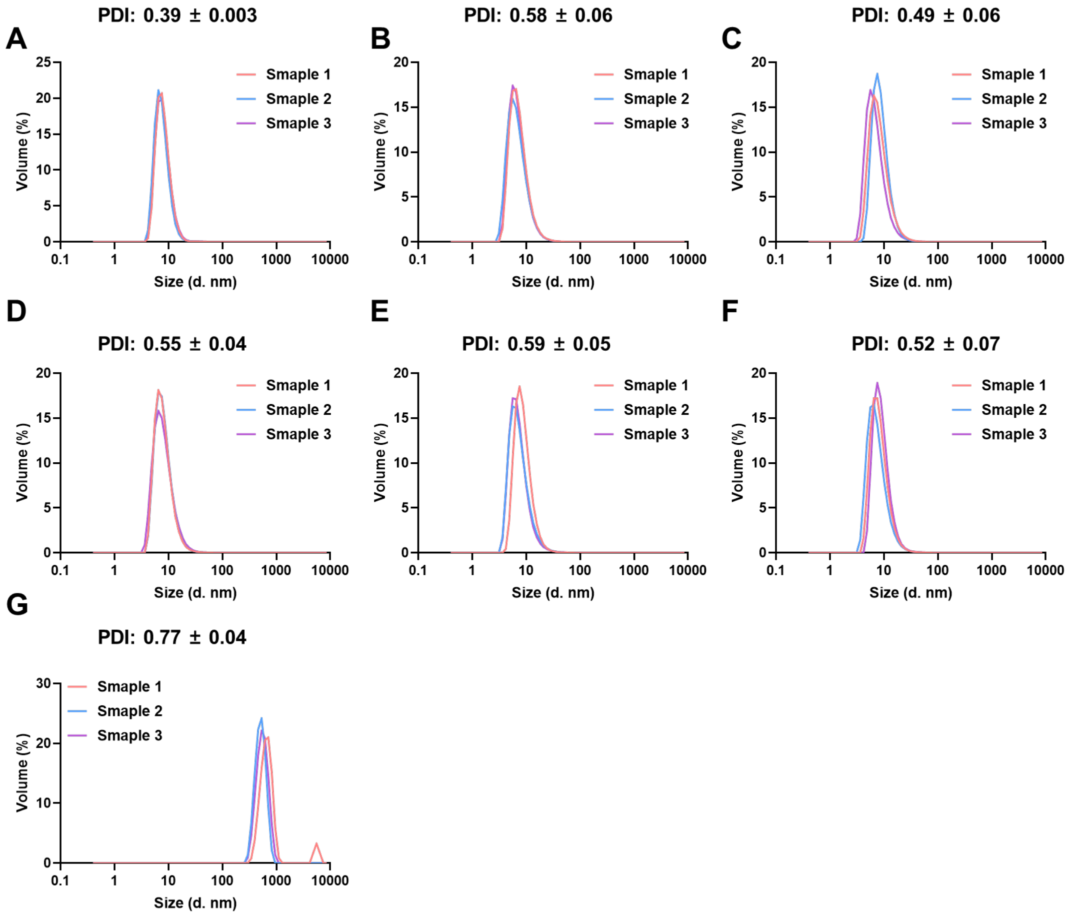

Figure S20. Size distribution of **Os2**. (A) PBS with 10% FBS solution at 0 h. (B-F) **Os2** in PBS with 10% FBS solution at 0 h (B), 4 h (C), 8 h (D), 24 h (E) and 48 h (F). (G) **Os2** in PBS solution at 0 h.

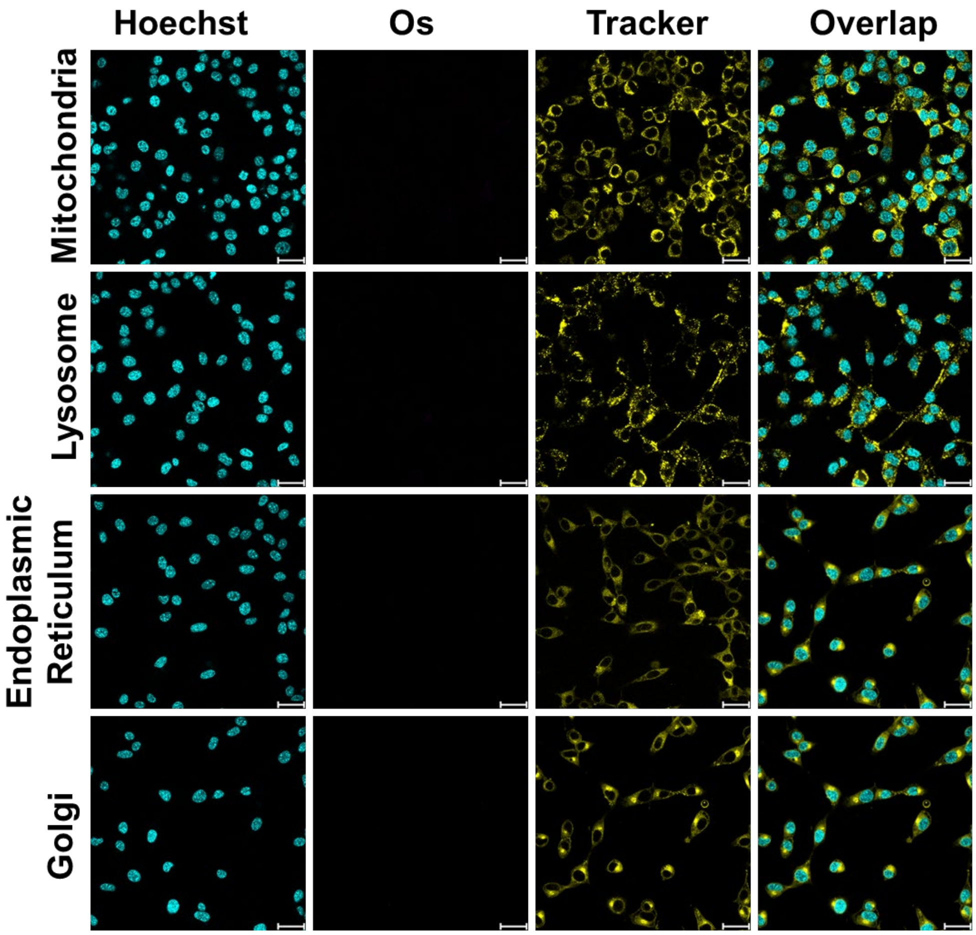

Figure S21. Confocal laser scanning microscopy images of trackers without **Os2**. Scale bar: 30 μm.

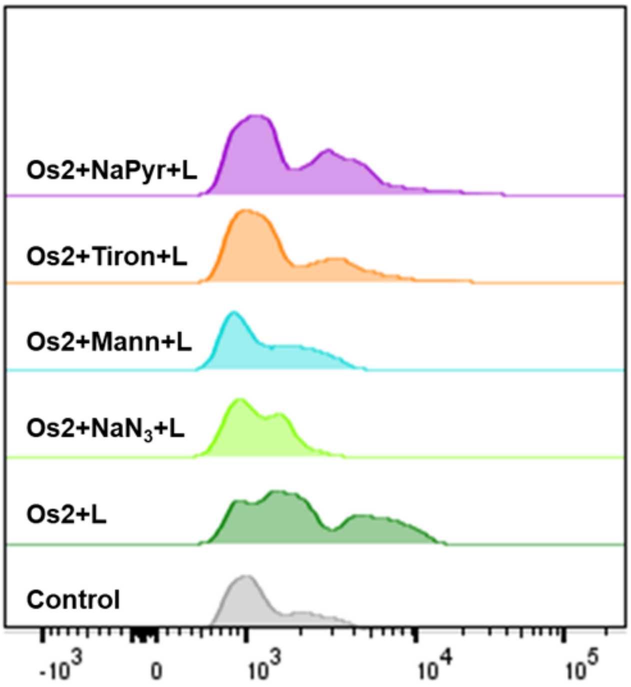

Figure S22. DCF levels in CT26 cells as assessed by flow cytometry. Cells were incubated with 0.76 μM of compound **Os2** in the presence of the indicated ROS scavengers (sodium azide, D-mannitol, sodium 4,5-dihydroxybenzene-1,3-disulfonate, sodium pyruvate) for 1 hour. After irradiation at 740 nm for 1 h (L), they were incubated with 10 μM DCFH-DA for 30 min at room temperature in the dark, collected, and analyzed by flow cytometry. Data was analyzed using overlays in FlowJo v10.

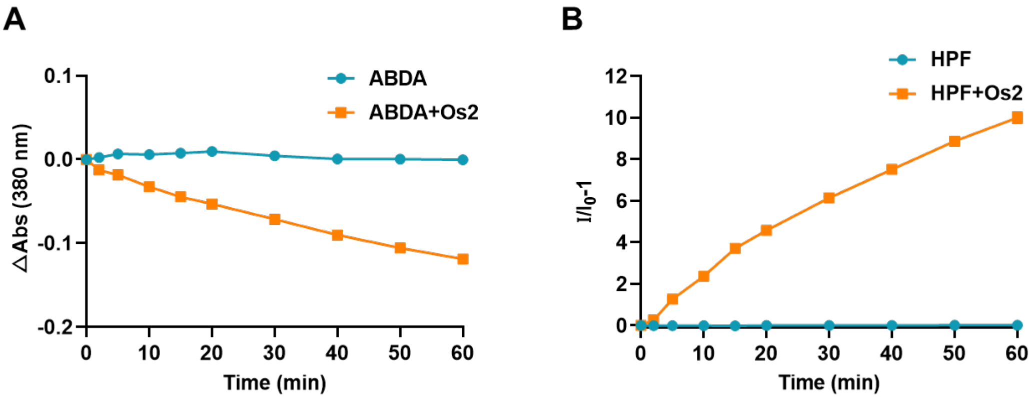

Figure S23. Time-dependent photogeneration of $^1O_2$ and •OH, as monitored by ABDA absorbance at 380 nm (A) and HPF fluorescence at 573 nm (B).

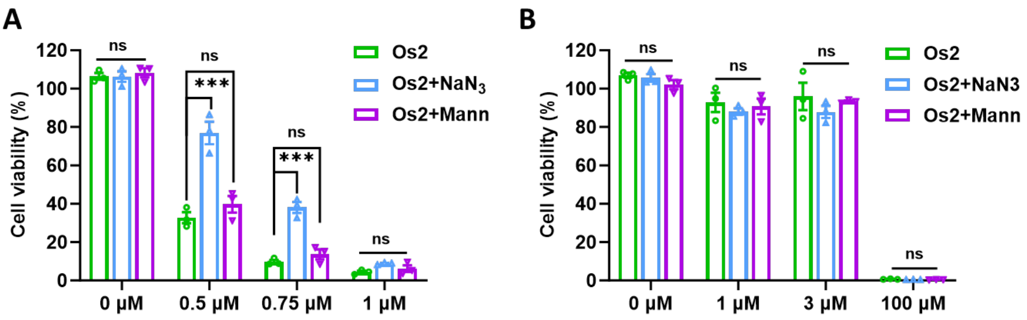

Figure S24. Cell viability of CT26 cells treated with **Os2** under irradiation or in the dark with or without ROS scavengers. All data are shown as mean ± SEM, n = 3. *P < 0.05, **P < 0.01, and ***P < 0.001 (two-way ANOVA followed by Tukey's HSD post-hoc test).

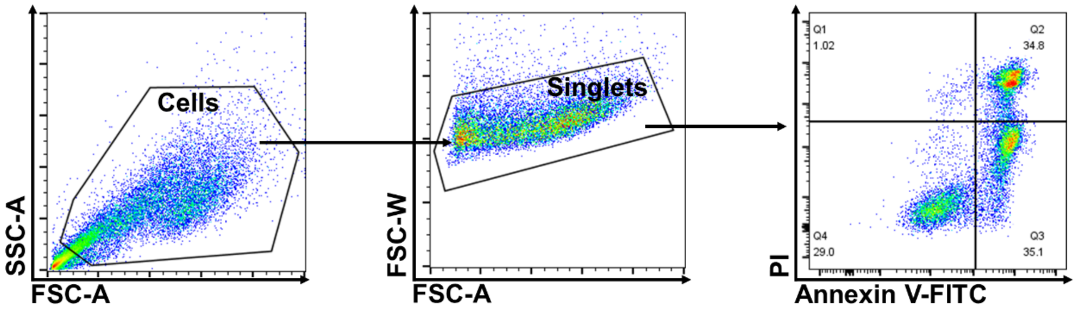

Figure S25. Gating strategies used for Annexin V/PI staining.

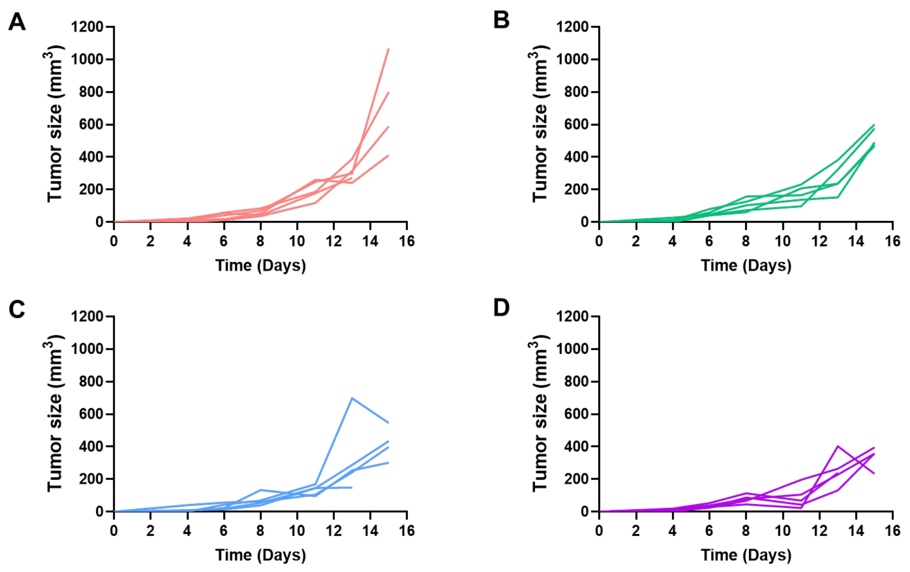

Figure S26. Tumor growth curves of individual mice in the control group (A), **Cisplatin** group (B), **Ce6** group (C) and **Os2** group.

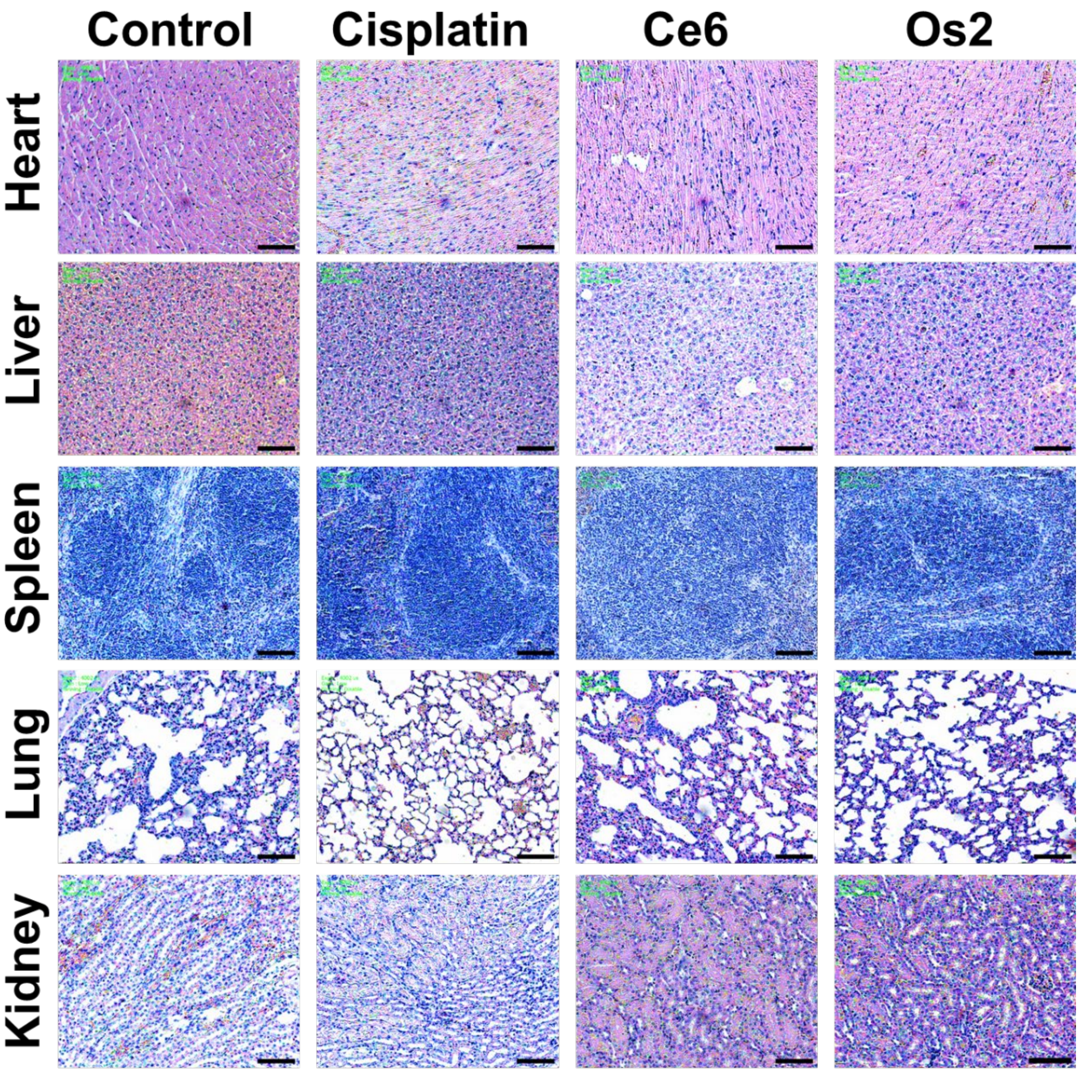

Figure S27. Histopathological images of the main organs, containing heart, liver, spleen, lung, and kidney obtained from mice after different treatment. Scale bar: 200 μm.

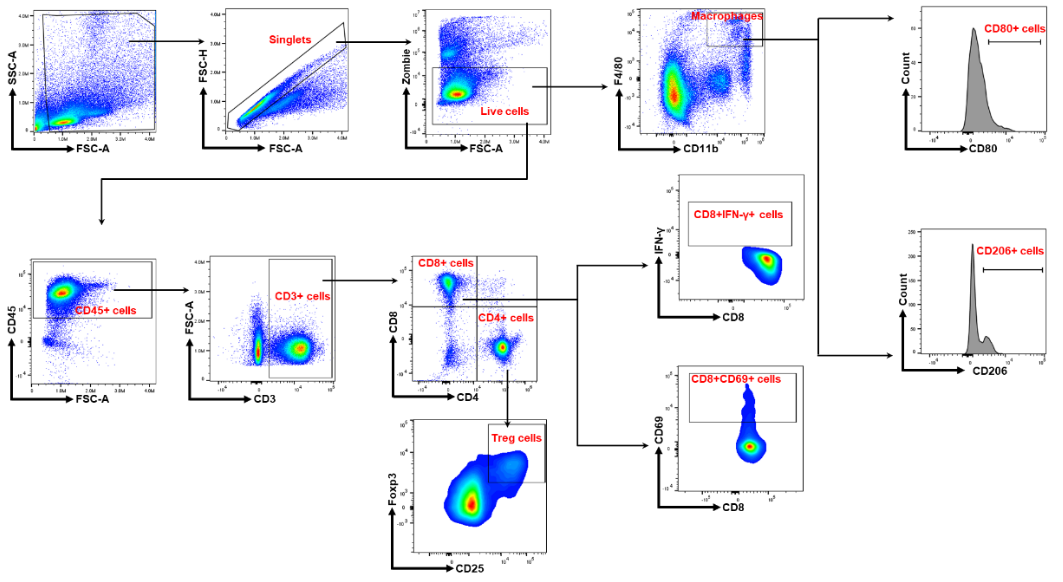

Figure S28. Gating strategies used for immune cells sorting in flow cytometry analysis.

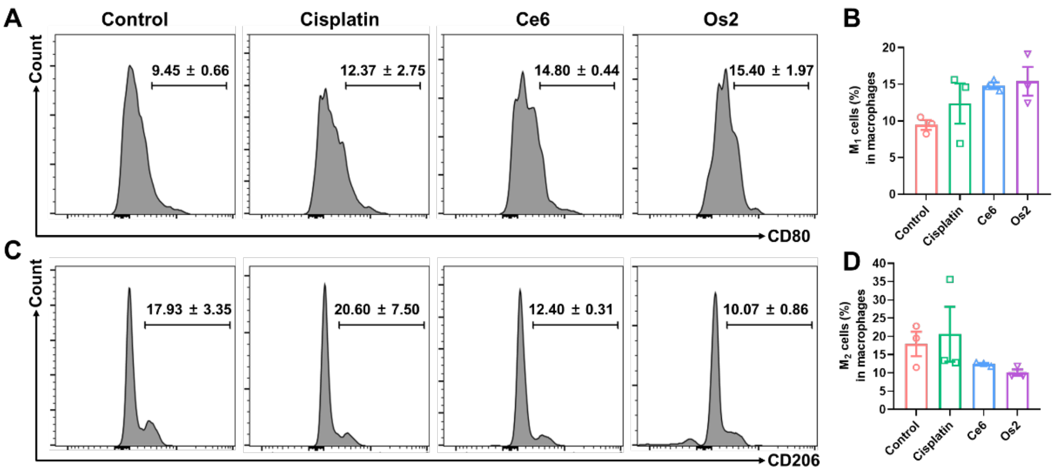

Figure S29. (A-B) Representative flow cytometry analysis of M1-like TAMs (A) and M2-like TAMs (B). (C-D) The quantification of M1-like TAMs (C) and M2-like TAMs (D).

Table S5. IC_{50} values upon excitation at 740 nm (1.12 mW/cm^2, 60 min, 12.60 J/cm^2) for **Os2**, **Ce6**, **PpIX** and **Hypericin** on mouse fibrosarcoma (MCA205) cells. Data are expressed as the mean ± SEM of three independent measurements.

PSs	IC_{50} (μM)
**Os2**	0.47 ± 0.02
**Ce6**	1.77 ± 0.38
**PpIX**	4.41 ± 0.09
**Hypericin**	1.66 ± 0.18

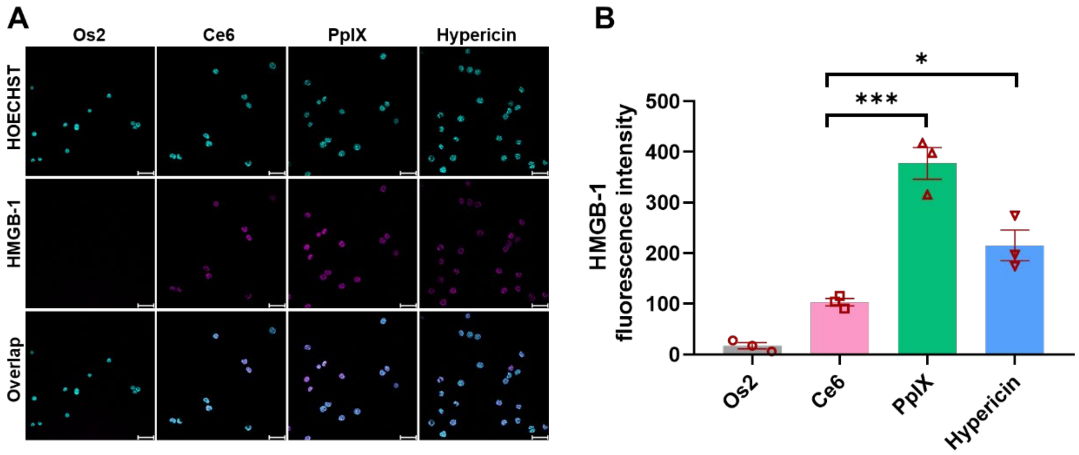

Figure S30. Detection of HMGB-1 release on MCA205 cells. (A) MCA205 cells were treated and irradiated as indicated, then immunostained against HMGB-1 (magenta). Nuclear staining with Hoechst 33342 (cyan). (B) Mean fluorescence intensity of corresponding HMGB-1 fluorescence in A. All data are shown as mean ± SEM, n = 3. *P < 0.05, **P < 0.01, and ***P < 0.001 (one-way ANOVA followed by Tukey's HSD post-hoc test). Scale bar: 30 μm.

References

1. A. J. Simaan, Y. Mekmouche, C. Herrero, P. Moreno, A. Aukauloo, J. A. Delaire, M. Reglier, T. Tron, *Chem. - Eur. J.* **2011**, *17*, 11743-11746. https://doi.org/10.1002/chem.201101282.
2. W. Xu, K. A. Kneas, J. N. Demas, B. A. Degraff, *Anal. Chem.* **1996,** *68*, 2605-2609. https://doi.org/10.1021/ac960083v.
3. E. J. McLaurin, A. B. Greytak, M. G. Bawendi, D. G. Nocera, *J. Am. Chem. Soc.* **2009,** *131*, 12994-3001. https://doi.org/10.1021/ja902712b.
4. A. Mani, T. Feng, A. Gandioso, R. Vinck, A. Notaro, L. Gourdon, P. Burckel, B. Saubamea, O. Blacque, K. Cariou, J. E. Belgaied, H. Chao, G. Gasser, *Angew. Chem., Int. Ed. Engl.* **2023**, *62*, e202218347. https://doi.org/10.1002/anie.202218347.
5. H. Ishida, S. Tobita, Y. Hasegawa, R. Katoh, K. Nozaki, *Coord. Chem. Rev.* **2010,** *254*, 2449-2458. https://doi.org/10.1016/j.ccr.2010.04.006.
6. C. Tanielian, C. Wolff, M. Esch, *J. Phys. Chem* **1996,** *100*, 6555-6560. https://doi.org/10.1021/jp952107s.
